# Identification of 3-[(4-Acetylphenyl)(4-Phenylthiazol-2-Yl)Amino]Propanoic Acid Derivatives as Promising Scaffolds for the Development of Novel Anticancer Candidates Targeting SIRT2 and EGFR

**DOI:** 10.3390/ph18050733

**Published:** 2025-05-16

**Authors:** Božena Golcienė, Povilas Kavaliauskas, Waldo Acevedo, Birutė Sapijanskaitė-Banevič, Birutė Grybaitė, Ramunė Grigalevičiūtė, Rūta Petraitienė, Vidmantas Petraitis, Vytautas Mickevičius

**Affiliations:** 1 Department of Organic Chemistry, Kaunas University of Technology, Radvilėnų Rd. 19, LT-50254 Kaunas, Lithuania; bozena.sovkovaja@ktu.edu (B.G.); povilas.kavaliauskas@som.umaryland.edu (P.K.); birute.sapijanskaite@ktu.lt (B.S.-B.); vytautas.mickevicius@ktu.lt (V.M.); 2 Department of Microbiology and Immunology, University of Maryland School of Medicine, Baltimore, MD 21201, USA; 3 Institute of Infectious Diseases and Pathogenic Microbiology, LT-59116 Prienai, Lithuania; ruta.petraitiene@hmh-cdi.org (R.P.); vidmantas.petraitis@hmh-cdi.org (V.P.); 4 Biological Research Center, Lithuanian University of Health Sciences, LT-44307 Kaunas, Lithuania; ramune.grigaleviciute@lsmuni.lt; 5 Instituto de Química, Facultad de Ciencias, Pontificia Universidad Católica de Valparaíso, Valparaíso 2373223, Chile; waldo.acevedo@pucv.cl; 6 Department of Animal Nutrition, Lithuanian University of Health Sciences, LT-44307 Kaunas, Lithuania; 7 Center for Discovery and Innovation, Hackensack Meridian Health, Nutley, NJ 07110, USA

**Keywords:** aminothiazoles, bisthiazolylphenylmethanes, hydrazones, hydrazides, oximes, anticancer properties, molecular docking

## Abstract

**Background:** A series of novel polysubstituted thiazole derivatives were synthesized, and their antiproliferative properties were evaluated using both 2D and 3D lung cancer models. **Methods:** The compounds were obtained via esterification, oximation, hydrazinolysis, and condensation reactions. **Results:** Structure–activity relationship analysis revealed that the antiproliferative activity was structure-dependent. Notably, oxime derivatives **21** and **22**, along with carbohydrazides **25** and **26**, exhibited low micromolar activity that was significantly greater than that of cisplatin (*p* < 0.005), a standard chemotherapeutic agent. These compounds demonstrated potent, antiproliferative activity against H69 small-cell lung carcinoma cells, as well as anthracycline-resistant H69AR cells. Moreover, compounds **21**, **22**, **25**, and **26** effectively induced cell death in A549 agarose-based 3D spheroids, further supporting their potential therapeutic application. The in silico studies proposed that compound **22** is able to interact with human SIRT2 and EGFR via conserved amino acid residues. **Conclusions:** The ability of these thiazole derivatives to target both drug-sensitive and drug-resistant lung cancer models highlights their promise as scaffolds for further optimization and preclinical development. Future studies will focus on structural modifications to enhance potency, selectivity, and pharmacokinetic properties, paving the way for the development of novel thiazole-based antiproliferative agents.

## 1. Introduction

Cancer remains one of the leading causes of death globally, with lung cancer being one of the most fatal subtypes, accounting for a significant proportion of cancer-related mortality. In 2020 alone, there were an estimated 19.3 million new cancer cases and nearly 10 million cancer-related deaths worldwide, a figure projected to rise to 28.4 million new cases by 2040 due to population aging and environmental factors [1,2]. Despite advances in treatment, including targeted and immune-based therapies, many cancers continue to pose challenges due to drug resistance and tumor heterogeneity.

Various cancers are known to exhibit alterations in key signaling pathways, checkpoint regulators, and growth factor receptor signaling, contributing to tumor initiation, progression, and metastatic processes [3,4,5]. Dysregulation of these pathways often results from genetic mutations, epigenetic modifications, or aberrant protein expression, leading to sustained proliferative signaling, evasion of apoptosis, and enhanced metastatic potential. To address these challenges, recent drug discovery efforts have focused on identifying small molecules capable of modulating multiple oncogenic signaling pathways. In particular, receptor tyrosine kinases (RTKs) such as the epidermal growth factor receptor (EGFR) are frequently mutated or overexpressed in NSCLC, contributing to sustained proliferative signaling and therapy resistance through the activation of the RAS-RAF-MEK-ERK and PI3K-AKT-mTOR pathways [6,7,8]. Moreover, epigenetic regulators such as sirtuin 2 (SIRT2), although not currently targeted by clinically approved anticancer drugs, have emerged as promising targets due to their roles in cell cycle control, metabolic regulation, and stress response modulation [9,10,11]. The development of dual-targeting small-molecule inhibitors that simultaneously modulate pathways such as EGFR and SIRT2 may provide a more robust therapeutic strategy by limiting compensatory mechanisms and overcoming resistance.

**Figure 1:**
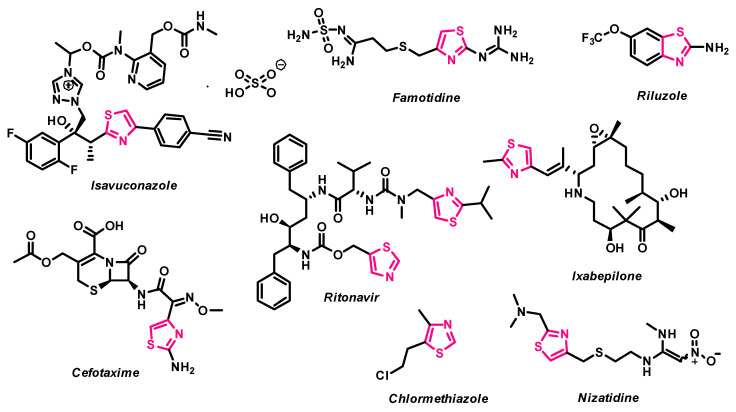
1,3-Thiazole based drug molecules.

Thiazole is a privileged heterocyclic scaffold in medicinal chemistry, with wide-ranging pharmacological applications, including anticancer, antibacterial, antifungal, and anti-inflammatory activities [12,13,14,15,16,17,18,19,20,21,22,23,24,25]. This moiety is found in numerous bioactive compounds, including natural products (e.g., thiamine, epothilones) and synthetic drugs such as Ixabepilone, Ritonavir, and Riluzole, all of which incorporate thiazole-based motifs and demonstrate clinical or investigational relevance in oncology [26,27,28,29,30] (Figure 1). The structural versatility of thiazole derivatives enables interactions with diverse biological targets and facilitates the design of molecules with improved pharmacokinetic and target-specific properties. Several thiazole-based compounds have been shown to exert anticancer effects via mechanisms such as apoptosis induction [31], tubulin polymerization inhibition [32], and topoisomerase interference [33]. Additionally, certain thiazole-containing molecules such as Nivolumab function as immune checkpoint inhibitors, further supporting the utility of this scaffold in cancer immunotherapy [34].

In our previous studies, we reported the synthesis of functionalized thiazole derivatives exhibiting antimicrobial and antioxidant activities [35,36,37,38]. Building upon this work, we now explore the antiproliferative potential of novel thiazole-based small molecules designed to interact with oncogenic targets. In this study, we present the synthesis and in vitro antiproliferative evaluation of a new series of 3-[(4-acetylphenyl)(4-phenylthiazol-2-yl)amino]propanoic acid derivatives. Based on structure–activity insights and molecular docking considerations targeting SIRT2 and EGFR, we introduced specific substitutions to explore the effects of electronic and steric variations on biological activity. The incorporation of bisthiazole derivatives was intended to examine whether expanding the thiazole system could enhance the target interactions. These compounds demonstrate promising structure-dependent anticancer activity in lung adenocarcinoma A549 cells, as well as in both drug-sensitive and multidrug-resistant small cell lung cancer cell lines H69 and H69AR. Notably, in silico analyses revealed that the most potent compound, derivative **22**, exhibits binding affinity toward both EGFR and SIRT2, indicating its potential as a dual-targeting antiproliferative agent for further development.

## 2. Results

### 2.1. Synthesis

**Scheme 1:**
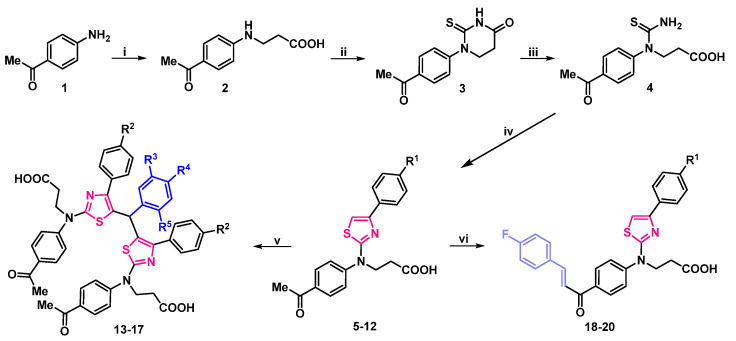
Synthesis of compounds **2**–**20**. (i) 1. acrylic acid, H_2_O, reflux, 15 h; 2. 10% NaOH; 3. AcOH to pH 6; (ii) 1. KSCN, AcOH, reflux 10 h; 2. HCl_conc._, reflux, 20 min; (iii) 1. 5% NaOH; heated until dissolved; 2. AcOH to pH 5; (iv) 1. 4-substituted phenacylbromide, Me_2_CO, reflux, 1–3 h; 2. CH_3_COONa, H_2_O, reflux, 5 min; (v) 1. ArCHO, Me_2_O, HCl_conc._, reflux, 5 h; 2. CH_3_COONa, H_2_O, reflux, 5 min; (vi) 1. KOH 30%, 4-FPhCHO, 60 °C, 10 h; 2. H_2_O, heated until dissolved; 3. AcOH to pH 5–6. **5** R^1^=H; **6** R^1^=Cl; **7** R^1^=NO_2_; **8** R^1^=CN; **9** R^1^=F; **10** R^1^=CF_3_; **11** R^1^=OH; **12** R^1^=OCF_3_; **13** R^2^=R^3^=R^5^=R^5^=H; **14** R^2^=R^3^=R^5^=H, R^4^=F; **15** R^2^=R^3^=R^5^=H, R^4^=Cl; **16** R^2^=R^4^=H, R^3^=R^5^=OCH_3_; **17** R^4^=F, R^2^=Cl, R^3^=R^5^=H; **18** R^1^=H; **19** R^1^=Cl; **20** R^1^=OCF_3_.

In this study, a range of functionalized 2,4-disubstituted-1,3-thiazoles were synthesized in order to evaluate their biological activity. First, compound **2** was resynthesized according to procedure discussed in [39] from a commercially available 4′-aminoacetophenone (**1**). Then, it was refluxed with potassium thiocyanate in acetic acid for 10 h, and subsequently cyclized by heating it with concentrated hydrochloric acid to obtain 1-(4-acetylphenyl)-2-thioxotetrahydropyrimidin-4(1*H*)-one (**3**). Light yellow compound **3** crystals were dissolved in sodium hydroxide solution, and after filtration acidified to pH 5, which yielded 3-[1-(4-acetylphenyl)thioureido]propanioc acid (**4**), which was then used in the synthesis of aminothiazole derivatives according to the *Hanthzsch* reaction (the condensation reaction of thioamides with *α*-halocarbonyl compounds). Refluxing 3-[1-(4-acetilphenyl)thioureido]propanioc acid (**4**) with 4-substituted phenacyl bromides in methanol gave thiazole hydrobromide salts, which dissolved in water, and heated at reflux with sodium acetate transformed into thiazol-2-yl propanoic acid derivatives **5**–**12** in moderate and good yields—46–88%. (Scheme 1).

The structure of the synthesized compounds was characterized by spectral data (IR, NMR spectra, and elemental analysis). The spectra showed good agreement with the assigned molecular structures. Newly formed thiazole ring’s proton signals of compounds **5**–**12** were noted in the aromatic part of ^1^H NMR spectrum at ~7.65 ppm, and 2nd, 4th, and 5th position carbon atoms in ^13^C NMR spectrum were observed at ~167.6, 150.8, and 104.5 ppm, respectively. Additional aromatic signals of *p-*substituted benzene ring were also noted (Supplementary Material, Figures S5–S20).

Condensation reaction of thiazole derivatives **5**, **6** with aromatic aldehydes in a 2:1 molar ratio, in acetone, in the presence of a catalytic amount of hydrochloric acid, afforded bis(thiazol-5-yl)phenylmethanes hydrochlorides, which poured with water, and refluxed with sodium acetate gave bright green bisthiazolylphenylmethanes **13**–**17** (Scheme 1). Compounds **13**–**17** structure was explained by their NMR, IR, and elemental analysis data. A singlet, integrated as one hydrogen atom at ~5.90 ppm in ^1^H NMR spectra and resonance line at ~40.2 ppm in ^13^C NMR spectra of bis(thiazol-5-yl)phenylmethanes **13**–**17** were attributed to the newly formed C–CH–C fragment. An increase in aromatic signals in both spectra was also confirmed (Supplementary Material, Figures S21–S30).

Carbonyl group containing compounds easily participate in various condensation reactions. Therefore, 3-{(4-acetylphenyl)[4-(4-substituted phenyl)thiazol-2-yl]amino}propanoic acids **5**, **6** were condensed with 4′-fluorobenzaldehyde in an aqueous potassium hydroxide solution at 60 °C temperature for 10 h. The resulting potassium salts were filtered off, dissolved in water, and acidified with dilute acetic acid to yield bright orange chalcones **18**–**20**. The lack of methyl group proton signals at ~2.60 ppm, sets of double singlets in 7.73–7.99 ppm interval, attributed to CH=CH group, and abundance of signals in the aromatic part of ^1^H NMR spectra is evidence of formation of compounds **18**–**20**. Theoretically, these compounds may exist as both—*E/Z* isomers. In 1,2-disubstituted alkene fragments, the coupling constants for the alkene hydrogens are always less for the *Z* isomer than for the *E* isomer. Typical values for *J*_H-H_ are 15 Hz for (*E*)-alkenes and 10 Hz for (*Z*)-alkenes. In chalcones **18**–**20** the CH=CH fragments coupling constants are *J* > 15 Hz, which may indicate, that in DMSO-*d*_6_ solution they exist as *E* isomers (Supplementary Material, Figures S31–S36).

**Scheme 2:**
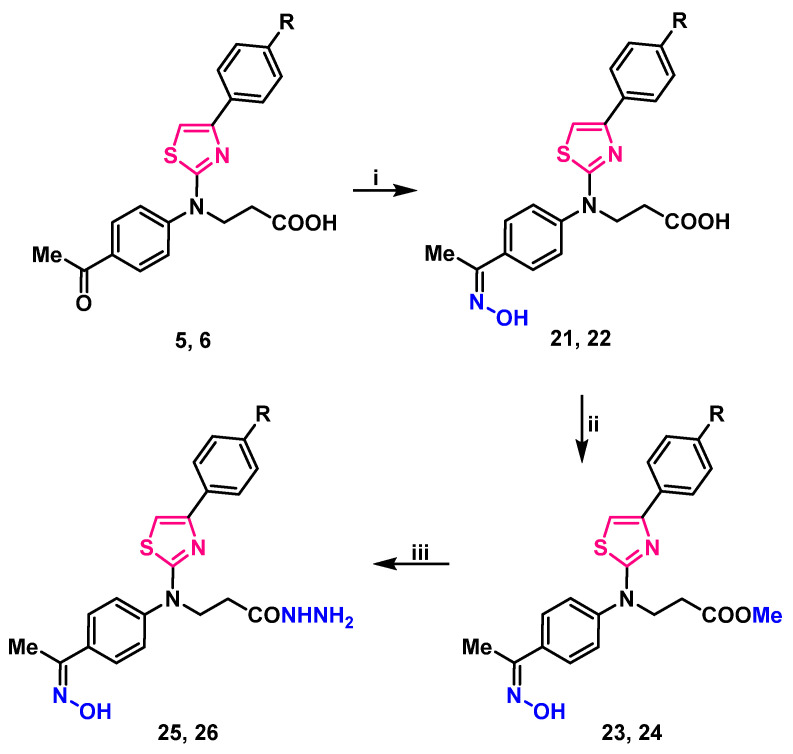
Synthesis and transformation of 3-/[4-(4-substituted phenyl)thiazol-2-yl]{4-[1-(hydroxyimino)ethyl]phenyl}amino/propanoic acids **21**, **22**. (i) HONH_2_·HCl, CH_3_COONa, 2-PrOH, reflux, 1 h; (ii) 1. MeOH, H_2_SO_4_, reflux, 4 h; 2. 5% Na_2_CO_3_ to pH 7; (iii) N_2_H_4_·H_2_O, 2-PrOH, reflux, 3 h. **5**, **21**, **23**, **25** R=H; **6**, **22**, **24**, **26** R=Cl.

Condensation of carbonyl compounds **5**, **6** with hydroxylamine hydrochloride in the presence of sodium acetate resulted in the formation of oximes **21**, **22**. Synthesis and transformation of 3-/[4-(4-substituted phenyl)thiazol-2-yl]{4-[1-(hydroxyimino)ethyl]phenyl}amino/propanoic acid **21**, **22** is depicted in Scheme 2.

Signals in downfield of ^1^H NMR spectra at 11.30 ppm and resonances at 3126, and 3124 cm^−1^ in the IR spectra, were attributed to the N–OH group of compounds **21**, **22** (respectively) (Supplementary Material, Figures S37–S40). These oximes underwent esterification with an excess of methanol under reflux in the presence of a catalytic amount of sulfuric acid, resulting in methyl 3-/[4-(4-substituted)thiazol-2-yl]{4-[1-(hydroxyimino)ethyl]phenyl}amino/propanoates **23**, **24**. Carbohydrazides **25**, **26** were synthesized by hydrazinolysis of methyl esters **23**, **24** with hydrazine hydrate in propan-2-ol under reflux (Scheme 2). Proton signals of NH_2_ group in ^1^H NMR spectra were identified as a multiplet, merged with one of the CH_2_ groups at 4.15–4.36 and 4.14–4.25 ppm, singlet at 9.10, and 9.09 was attributed to NH group proton. N–OH group proton singlet slightly shifted up-field to 11.29 ppm if compared with the esters **23**, **24** resonances, which were at 11.31 ppm. Elemental analysis data is in good compliance with calculated values (Supplementary Material, Figures S41–S48).

**Scheme 3:**
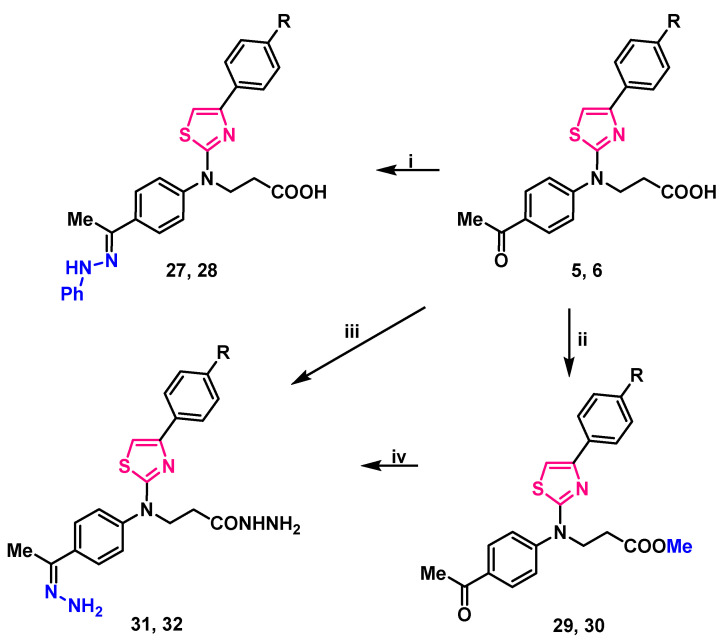
Synthesis of compounds **27**–**32**. (i) PhNHNH_2_, 2-PrOH, reflux 2 h; (ii) 1. MeOH, H_2_SO_4_, reflux, 7 h; 2. 5% Na_2_CO_3_ to pH 7; (iii) N_2_H_4_·H_2_O, toluene, reflux, 12 h; (iv) N_2_H_4_·H_2_O, 1,4-dioxane, reflux, 24 h. **5**, **27**, **29**, **31** R=H; **6**, **28**, **30**, **32** R=Cl.

Stirring 3-(thiazol-2-yl)amino propanoic acid **5**, **6** in propan-2-ol, at mixtures boiling temperature with phenylhydrazine, yielded 3-/[4-(4-substituted phenyl)thiazol-2-yl]{4-[1-(2-phenylhydrazineylidene)ethyl]phenyl}amino/propanoic acids **27**, **28** (Scheme 3).

Analyzing compounds **27**, **28** NMR spectra, signals at 9.35 ppm in ^1^H NMR spectra were assigned to a newly formed =N–NH– group proton, the lack of carbonyl group signal, and the abundance of aromatic carbon atom signals of the third benzene ring in ^13^C NMR spectra was noted (Supplementary Material, Figures S49–S52). Resonances at 3108 ant 3104 cm^−1^ (NH) in compounds **27**, **28** FT-IR spectra (respectively) also support the identified structure of these 3-/{4-[1-(2-phenylhydrazineylidene)ethyl]phenyl}{4-(4-subsituted phenyl)thiazol-2-yl)amino/propanoic acids.

Reaction of thiazole derivatives **5**, **6** with hydrazine monohydrate in toluene, at mixtures boiling temperature for 12 h gave a hydrazone and hydrazide moieties containing compounds **31**, **32** which may also be synthesized by stirring methyl esters **29**, **30** with hydrazine monohydrate in 1,4-dioxane at reflux for twice as long—24 h (Scheme 3). Both methods gave approximately the same yield (52–58%).

### 2.2. Structure-Dependent Antiproliferative Activity of 3-[(4-Acetylphenyl)(4-Phenylthiazol-2-Yl)Amino]Propanoic Acid Derivatives

**Figure 2:**
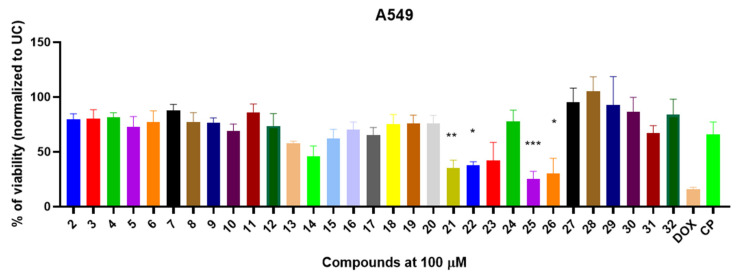
The in vitro antiproliferative activity of compounds **2**–**32** against A549 human lung adenocarcinoma cell lines. The A549 were exposed with compounds or doxorubicin (DOX) and cisplatin (CP) for 24 h and the viability was determined using MTT assay and normalized to untreated control (UC). Data shown are mean ± SD values from three separate experiments for each group. The significance of the data was determined using a one-way ANOVA test. * *p* < 0.05, ** *p* < 0.001, *** *p* < 0.0002.

Following the successful synthesis and characterization of compounds **2**–**32**, their in vitro antiproliferative activity was evaluated using the well-established A549 human lung adenocarcinoma cell model [40,41,42,43]. To identify the most promising antiproliferative candidates, A549 cells were treated with a fixed concentration (100 µM) of each compound, and the cytotoxic effects were compared to those of the standard FDA-approved chemotherapeutic agents doxorubicin (DOX) and cisplatin (CP) (Figure 2).

The initial series of starting compounds, **2**–**4**, and thiazoles **5**–**12**, bearing various aromatic substitutions, exhibited moderate antiproliferative activity, reducing A549 cell viability to 69.2–87.8% (Figure 2). Notably, these compounds did not demonstrate significantly greater antiproliferative activity compared to DOX or CP.

Bisthiazoylphenylmethane derivative **13**, bearing only three Ph substituents, reduced A549 cell viability to 57.6%, while compound **14**, incorporating two Ph and one 4-F-Ph substitution, exhibited moderate antiproliferative activity, resulting in 47.8% viability. Surprisingly, the introduction of Cl-Ph and MeO-Ph substitutions in bisthiazolylmethanes **15**–**17** led to a decrease in antiproliferative activity, with A549 viability ranging from 62.2% to 70.4%. These findings suggest that substituents present at those locations in the molecule are critical for the antiproliferative activity of the synthesized bisthiazolylmethanes (Figure 2). Furthermore, carbonyl-containing derivatives **18**–**20** displayed weak antiproliferative activity, reducing A549 viability to 75.3–76.0% (Figure 1).

The synthesized oxime derivatives **21**, **22** exhibited significantly greater antiproliferative activity against A549 cells than cisplatin (*p* < 0.005). Specifically, compound **21** reduced A549 viability to 35.3%, while compound **22** decreased viability to 37.6%. Both compounds displayed significantly higher activity than cisplatin (*p* = 0.0095 and *p* = 0.0211, respectively), which reduced A549 viability to 65.9%. Esterification of compounds **21**, **22** led to methyl ester derivatives **23**, **24**, which showed diminished antiproliferative activity (41.9% and 77.7%, respectively) (Figure 2). Further modification yielded carbohydrazide derivatives **25**, **26**, which exhibited significantly greater antiproliferative activity (*p* < 0.005) than cisplatin. Compound **25** reduced A549 viability to 25.0% (*p* = 0.002), while compound **26** resulted in 30.5% viability (*p* = 0.003).

The 3-[4-(4-substituted phenyl)thiazol-2-yl]{4-[1-(2-phenylhydrazinylidene)ethyl]phenyl}amino]propanoic acid derivatives **27**, **28**, along with their methyl esters **29**, **30**, as well as hydrazone and hydrazide derivatives **31**, **32**, demonstrated limited antiproliferative activity. Compounds **27**–**30** did not exhibit significant cytotoxicity against A549 cells (viability: 82.4–100%), while hydrazide **31** and hydrazone **32** reduced A549 viability to 67.1% and 84.0%, respectively.

To confirm the in vitro antiproliferative activity of the most promising derivatives **21**, **22**, **25**, and **26**, we performed dose–response experiments (Figure S61) and determined their IC_50_ values (Table 1). All tested compounds demonstrated promising dose-dependent antiproliferative activity (Figures S61 and S62).

The compounds demonstrated low micromolar inhibitory activity, with IC_50_ values ranging from 5.42 to 25.4 µM. Compounds **21** and **22** exhibited the most potent activity, with IC_50_ values of 5.42 and 2.47 µM, respectively. In contrast, compounds **25** and **26** showed notably higher IC_50_ values of 8.05 and 25.4 µM, respectively (Table 1). Furthermore, compounds **21**, **22**, and **25** showed higher antiproliferative activity than cisplatin (CP) (IC_50_ = 11.71 µM) (Table 1).

These findings indicate that 3-[(4-acetylphenyl)(4-phenylthiazol-2-yl)amino]propanoic acid derivatives exhibit structure-dependent antiproliferative activity against A549 cells. Among them, carboxylic acids **21**, **22** and carbohydrazides **25**, **26** with hydroxyimino fragment in the molecules demonstrated the most promising antiproliferative effects, significantly surpassing the cytotoxic activity of cisplatin.

### 2.3. Most Promising 3-[(4-Acetylphenyl)(4-Phenylthiazol-2-Yl)Amino]Propanoic Acid Derivatives Exhibits Antiproliferative Activity Against Drug Sensitive H69 and Resistant H69AR Cells

**Figure 3:**
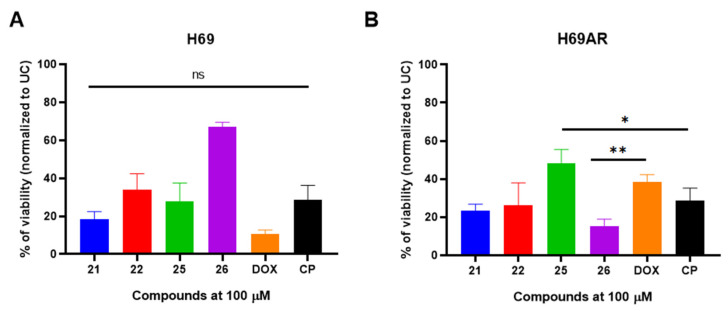
The selected most promising compounds **21**, **22**, **25**, **26** exhibits in vitro antiproliferative activity against susceptible H69 (panel (**A**)) and anthracycline-resistant H69AR (panel (**B**)) human lung carcinoma cells. The cells were exposed with compounds or doxorubicin (DOX) and cisplatin (CP) for 24 h and the viability was determined using MTT assay and normalized to untreated control (UC). Data shown are mean ± SD values from three separate experiments for each group. The significance of the data was determined using a one-way ANOVA test. * *p* < 0.05, ** *p* < 0.001, ns—not significant.

After selecting the most promising compounds (**21**, **22**, **25**, **26**), we hypothesized whether the observed antiproliferative activity was specific to the A549 cell line or if it extended to other lung-derived cancer models. To investigate this, we assessed the cytotoxic effects of these compounds on H69 human lung carcinoma cells. Furthermore, to evaluate their impact on cancer cells with pre-existing multidrug resistance mechanisms, we performed cytotoxicity assays using anthracycline-resistant H69AR cells (Figure 3A,B).

All selected compounds demonstrated significant antiproliferative activity in both H69 and H69AR cells compared to the untreated control (UC) (*p* < 0.05). Oxime derivatives **21** and **22** reduced H69 cell viability to 18.3% and 33.9%, respectively (Figure 3A). Notably, when anthracycline-resistant H69AR cells were exposed to compounds **21** and **22**, a comparable reduction in viability was observed (23.5% and 26.6%, respectively) (Figure 3B).

Carbohydrazide **25** exhibited potent cytotoxic activity against drug-sensitive H69 cells, reducing viability to 27.7%; however, its activity was attenuated in drug-resistant H69AR cells, with viability remaining at 48.4% (Figure 3A,B). Unexpectedly, carbohydrazide **26** displayed relatively weak antiproliferative activity against H69 cells (67.0% viability) but exhibited pronounced cytotoxic effects against drug-resistant H69AR cells, reducing viability to 15.4%. Furthermore, the cytotoxic activity of compound **25** against H69AR cells was significantly greater than that of doxorubicin (*p* = 0.0043).

**Figure 4:**
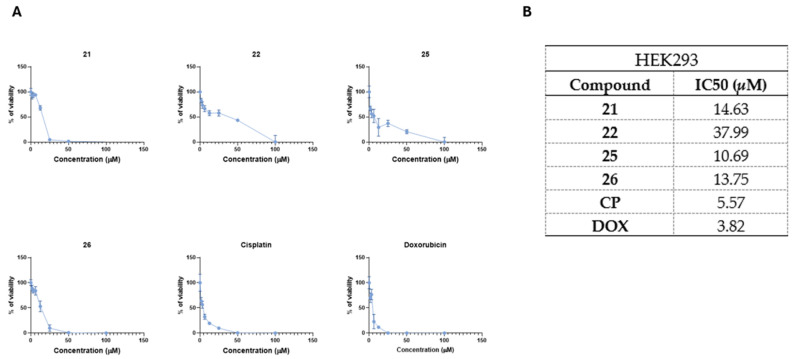
The selected most promising compounds **21**, **22**, **25**, **26** demonstrate favorable cytotoxicity profiles in non-cancerous HEK293 human embryonic kidney cells. Panel (**A**) shows dose–response kinetics of tested compounds in HEK293 cells, while panel (**B**) shows calculated IC_50_ values. The cells were exposed to compounds or doxorubicin (DOX) and cisplatin (CP) for 24 h and the viability was determined using MTT assay. Data shown are mean ± SD values from three separate experiments for each group.

After characterizing the cytotoxic activity of the selected compounds in cancerous cells, we next aimed to evaluate their cytotoxic effects on non-cancerous human cells. To do so, HEK293 human embryonic kidney cells were exposed to compounds **21**, **22**, **25**, and **26**, and cell viability was assessed (Figure 4).

All compounds showed lower antiproliferative activity in non-cancerous HEK293 cells in comparison to CP (IC_50_ = 5.57 µM) or DOX (IC_50_ = 3.82 µM) (Figure 4A,B). Among these compounds, compound **22** showed the lowest cytotoxicity in HEK293 cells (IC_50_ = 37.99 µM), followed by compounds **21** (IC_50_ = 14.63 µM), **26** (IC_50_ = 13.75 µM), and **25** (IC_50_ = 10.69 µM) (Figure 4A,B).

Collectively, these results demonstrate that the antiproliferative activity of compounds **21**, **22**, **25**, and **26** is not cell-line-dependent. Moreover, these compounds exhibit cytotoxic activity against the drug-resistant H69AR cell line while maintaining favorable cytotoxicity profiles in non-cancerous HEK293 cells.

### 2.4. The 3-[(4-Acetylphenyl)(4-Phenylthiazol-2-Yl)Amino]propanoic Acid Derivatives ***21***, ***22***, ***25***, and ***26*** with Hydroxyimino Moiety Induces the Cytotoxic Activity in A549 Derived Cancer Spheroids

After initial screening on 2D monolayer cultures, we transitioned to A549 agarose-based spheroids to better replicate the three-dimensional architecture, cellular interactions, and drug diffusion barriers characteristic of solid tumors. To visualize the A549 spheroid response to treatment with the test compounds, we performed acridine orange/propidium iodide (AO/PI) staining, which enabled the assessment of spheroid architecture and the spatial distribution of live and dead cells. Additionally, to quantitatively evaluate treatment-induced cytotoxicity, we employed an LDH release assay, which provides a reliable measure of cell membrane integrity and treatment-induced cell death.

**Figure 5:**
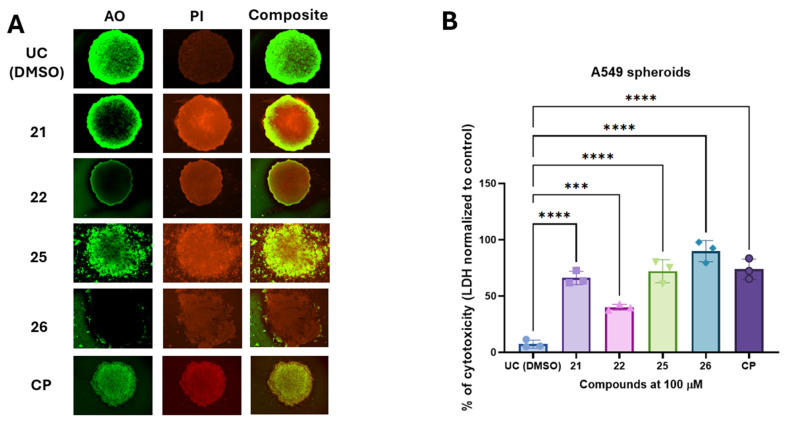
The selected compounds induce cytotoxicity in the A549 agarose-based spheroid model. A549-derived spheroids were exposed to the test compounds or cisplatin (CP) for 24 h. The cytotoxic response induced by treatment was visualized using acridine orange/propidium iodide (AO/PI) staining (panel (**A**)). Cytotoxicity, as measured by lactate dehydrogenase (LDH) release in spheroid media, was quantified using an LDH release assay and normalized to untreated controls (UC) (panel (**B**)). Data are presented as mean ± SD from three experimental replicates for each group. Statistical significance was determined using a one-way ANOVA test. *** *p* < 0.001, **** *p* < 0.0001.

Acridine orange/propidium iodide staining was performed to visualize spheroid morphology and cell viability following treatment with compounds **21**, **22**, **25**, and **26** at 100 µM (Figure 5A). Untreated control (UC) spheroids exhibited a uniform green fluorescence, indicating high viability with minimal PI-positive dead cells. Treatment with compounds **21** and **22** resulted in partial cell death, with spheroids retaining structural integrity but displaying increased PI fluorescence, particularly at the periphery. In contrast, treatment with compounds **25** and **26** led to substantial disruption of spheroid architecture and widespread PI-positive staining, suggesting a higher degree of cytotoxicity in A549 derived tumor spheroids.

To quantify treatment-induced cytotoxicity in A549 derived spheroids, we measured LDH release from spheroids after exposure to the test compounds (Figure 4B). The untreated control group exhibited minimal cytotoxicity (7.2%). In contrast, compounds **21** and **22** induced significant cytotoxic effects, with LDH release reaching 66.7% and 40.6%, respectively. Carbohydrazide **25** exhibited strong cytotoxic activity (72.1%), whereas compound **26** demonstrated the highest cytotoxicity among the tested compounds, with LDH release reaching 89.9%. Statistical analysis confirmed that all tested compounds significantly increased cytotoxicity compared to the untreated control (*p* < 0.005).

Collectively, these findings indicate that compounds **25** and **26** exert the most pronounced cytotoxic effects in the 3D spheroid model, suggesting their potential as promising scaffold for further development of antiproliferative agents targeting lung derived tumors.

### 2.5. Molecular Docking Studies

After successfully demonstrating the in vitro antiproliferative activity of compounds **21**, **22**, **25** and **26** using 2D and 3D cell culture models, we performed some in silico molecular docking studies to identify possible biological targets for the cytotoxic compounds (**21**, **22**, **25** and **26**) in order to obtain some information on their possible mechanism of action.

**Figure 6:**
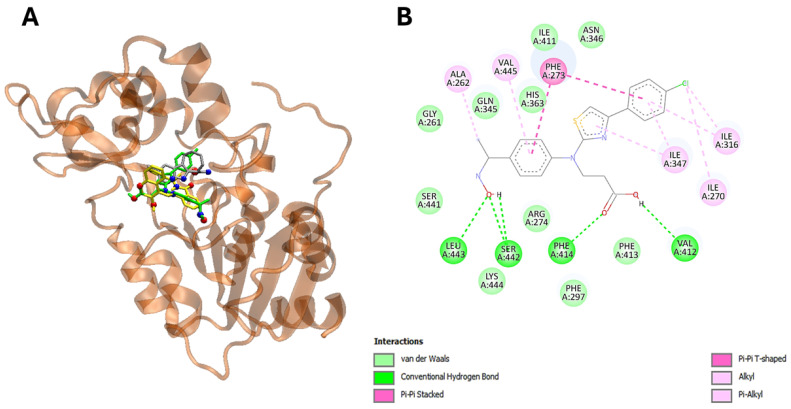
The compound **22** is able to interact with SIRT1 protein in silico. Panel (**A**) demonstrates visualization of the potential binding site of compound **22** (in green), ligand (in silver) and sirtinol (in yellow) into SIRT1 protein. Panel (**B**) shows plotted 2D maps of H-bonds and hydrophobic interactions of compound **22** with SIRT1. residues. Van der Waals, Pi-Pi stacked, alkyl and Pi-alkyl are considered hydrophobic interactions.

**Figure 7:**
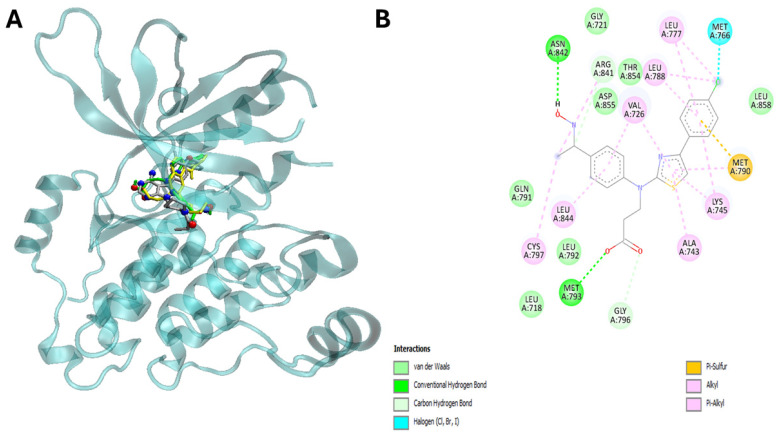
The compound **22** is able to interact with human epidermis growth factor receptor (EGFR). Panel (**A**) shows visualization of the potential binding site of compound (in green), ligand (in red) and erlotinib (in yellow) into EGFR. Panel (**B**) shows plotted 2D maps of H-bonds and hydrophobic interactions of compound **22** with EGFR residues. Van der Waals, Pi-sulfur, alkyl and Pi-alkyl are considered hydrophobic interactions.

For this purpose, we predicted the potential docking sites of the compounds in several cancer-related proteins and calculated their corresponding binding energies (∆G_bin_). To obtain high reliability results, we reduced the search space to a set of cancer-related proteins of known 3D structures by establishing independent searches with the set of compounds, and we used their most stable conformers interacting with the biological targets. Table 2 shows the results of such a screening, and globally indicates that most cytotoxic compounds bind stronger to NAD-dependent sirtuin-1 deacetylase (SIRT1) (Figure 6A,B), with ∆G_bin_ values ranging from −10.2 to −9.5 (average −9.85) kcal/mol, and epidermal growth factor receptor (EGFR) with values ranging from −9.5 to −9.2 (average −9.35) kcal/mol (Figure 7A,B).

Interestingly, the energetic aspects of the interactions produced results favorable to **22** in comparison to ligand and erlotinib with a favorable energy difference of 3.3 and 0.9 kcal/mol for EGFR (Table 3, Figure 6A,B). On the other hand, interactions did not provide a result favorable to **22** in comparison to ligand and sirtinol with an energy difference of −1.5 and −2.0 kcal/mol for SIRT2 (Table 2 and Table 3). Furthermore, in silico analysis shows that compound **22** is able to interact with SIRT2 by hydrogen bonding via Val 412, Phe414, Ser442 and Leu443 (Figure 6A,B, Table 3). On the other hand, compound **22** targets EGFR via hydrogen bonding through Met793 and Asn842 (Table 3, Figure 7A,B).

Importantly, the aromatic ring and thiazole moiety of these compounds play a pivotal role in these interactions, directly contributing to the overlap with the ligands at the catalytic sites of the enzymes (Table 3) (Figure 6A,B and Figure 7A,B).

Collectively, these in silico screening results indicate that compound **22** is able to target both SIRT2 and EGFR via conserved amino acids, thus potentially modulating the SIRT2/EGFR axis in cancerous cells leading to the cell death.

## 3. Discussion

In this study, we describe the synthesis and in vitro antiproliferative activity characterization of novel 3-[(4-acetylphenyl)(4-phenylthiazol-2-yl)amino]propanoic acid derivatives using human lung-derived 2D and 3D preclinical cancer screening models. The compounds **2**–**32** demonstrated promising, structure-dependent antiproliferative activity, with compounds **21**, **22**, **25**, and **26** showing the most promising antiproliferative activity in A549 cells.

The SAR analysis of 3-[(4-acetylphenyl)(4-phenylthiazol-2-yl)amino]propanoic acid derivatives revealed that the presence of an oxime moiety significantly enhances antiproliferative activity. Compounds **21** and **22**, bearing a hydroxyimino (-C=NOH) functional group, exhibited the most potent cytotoxicity against A549 cells, with IC_50_ values of 5.42 µM and 2.47 µM, respectively, surpassing the efficacy of cisplatin. This finding suggests that the oxime functionality plays a critical role in enhancing bioactivity, likely through its electron-withdrawing properties or potential hydrogen bonding interactions with cellular targets. However, esterification of the carboxyl group, as seen in compounds **23** and **24**, resulted in a marked reduction in antiproliferative activity, highlighting the importance of free carboxyl groups for optimal cytotoxic effects. Further modifications leading to carbohydrazide derivatives **25** and **26** maintained significant antiproliferative activity, with compound **25** demonstrating an IC_50_ of 8.05 µM. Interestingly, while oxime derivatives displayed consistent activity across A549, H69, and drug-resistant H69AR cells, carbohydrazide **26** exhibited a selective cytotoxic profile, being more effective against H69AR cells than drug-sensitive H69 cells. This suggests a possible mechanistic distinction between oxime and carbohydrazide derivatives in targeting resistant cancer phenotypes. Moreover, the most active compounds (**21**, **22**, **25**) demonstrated relatively low cytotoxicity against non-cancerous HEK293 cells, indicating a degree of selectivity. The in silico docking studies identified that compound **22** is able to interact with human SIRT2 and EGFR proteins (often found to be mutated or overexpressed in various cancer cell lines) with promising antiproliferative activity. Compound **22** interacts with SIRT2 and EGFR via diverse amino acid derivatives, opening the possibility of developing a novel compound **22**-based, dual acting derivative targeting both SIRT2 and EGFR.

These results suggest that the 3-[(4-acetylphenyl)(4-phenylthiazol-2-yl)amino]propanoic acid derivatives are promising scaffolds for further development in lung cancer therapies, with enhanced efficacy against drug-resistant cancer cells and favorable toxicity profiles in non-cancerous cells. Further studies are needed to better understand the molecular targets, in vivo safety and activity of 3-[(4-acetylphenyl)(4-phenylthiazol-2-yl)amino]propanoic acid scaffold.

## 4. Materials and Methods

### 4.1. Synthesis

Reagents and solvents were purchased from Sigma-Aldrich (St. Louis, MO, USA) and used without further purification. The reaction course and purity of the synthesized compounds were monitored by TLC using aluminum plates pre-coated with Silica gel with F254 nm (Merck KGaA, Darmstadt, Germany). Melting points were determined with a B-540 melting point analyzer (Büchi Corporation, New Castle, DE, USA) and were uncorrected. IR spectra (ν_max_, cm^−1^) were recorded on a Vertex70 FT–IR spectrometer (Bruker, JAV, Billerica, MA, USA) using KBr pellets. NMR spectra were recorded on a Brucker Avance III (400, 101 MHz) spectrometer (Bruker BioSpin AG, Fällanden, Switzerland). Chemical shifts were reported in (δ) ppm relative to tetramethylsilane (TMS), with the residual solvent as internal reference (DMSO-d_6_, δ = 2.50 ppm for ^1^H NMR and δ = 39.5 ppm for ^13^C NMR or Acetone-d_6_, δ = 2.05 ppm for ^1^H NMR). Data are reported as follows: chemical shift, multiplicity, integration, coupling constant [Hz], and assignment. Elemental analyses (C, H, N) were conducted using the Elemental Analyzer CE-440 (Exeter Analytical, Inc., Chelmsford, MA, USA); their results were found to be in good agreement (±0.3%) with the calculated values.

Compound **1** was purchased from Labochema LT, UAB, Vilnius, Lithuania.

Compound **2** was synthesized according to the procedure reported in [39]. The ^1^H and ^13^C-NMR spectra agreed with that given in the study.

### 4.2. General Procedure for the Synthesis of ***3***

A mixture of 3-((4-acetylphenyl)amino)propanoic acid (**2**) (0.01 mol, 2.07 g), potassium thiocyanate (0.03 mol, 2.91 g) and acetic acid (15 mL) was refluxed for 10 h. Then, concentrated hydrochloric acid (6 mL) was added. The reaction mixture was heated at the mixture’s boiling temperature for 20 min more. After the completion of reaction, content was diluted with water (50 mL), and cooled down. The crystalline formed were filtered off, washed with water and crystallized.

*1-(4-Acetylphenyl)-2-thioxotetrahydropyrimidin-4(1H)-one* (**3**): light brown solid, yield 0.81 g (33%); m.p. 265–266 °C (1,4-dioxane); IR (KBr) (ν_max_, cm**^−^**^1^): 1205 (C=S), 1679, 1708 (2C=O), 3123 (NH); 1H NMR (400 MHz, DMSO-d_6_) δ (ppm): 2.60 (s, 3H, CH_3_), 2.83 (t, 2H, *J* = 6.7 Hz, CH_2_), 3.94 (t, 2H, *J* = 6.7 Hz, CH_2_), 7.52 (d, 2H, *J* = 8.0 Hz, H_Ar_), 8.01 (d, 2H, *J* = 8.1 Hz, H_Ar_), 11.37 (s, 1H, NH); ^13^C NMR (101 MHz, DMSO-d_6_) δ (ppm): 26.9 (CH_3_), 30.4 (CH_2_), 48.6 (CH_2_), 127.5, 129.2, 135.6, 149.0, 167.0 (NHC=O), 179.5 (C=S), 197.3 (C=O); Anal. Calcd. for C_12_H_12_N_2_O_2_S: C 58.05; H 4.87; N 11.28%; Found: C 58.35; H 4.82; N 11.35%.

### 4.3. General Procedure for the Synthesis of ***4***

1-(4-Acetylphenyl)-2-thioxotetrahydropyrimidin-4(1*H*)-one (**3**) (0.14 g, 0.56 mmol) was dissolved by heating in sodium hydroxide solution (5 mL, 5%), then cooled down, and filtered off. Filtrate was acidified with acetic acid to pH 5. The resulting precipitate was filtered off, washed with water and dried.

*3-[1-(4-Acetylphenyl)thioureido]propanoic acid* (**4**): light yellow solid, yield 0.15 g (83%); m.p.170–171 °C (propan-2-ol); IR (KBr) (ν_max_, cm**^−^**^1^): 1269 (C=S), 1678, 1714 (2C=O), 3182 (OH), 3343 (NH_2_); ^1^H NMR (400 MHz, Acetone-d_6_) δ (ppm): 2.61 (s, 3H, CH_3_), 2.76 (t, 2H, *J* = 7.6 Hz, CH_2_), 4.39 (t, 2H, *J* = 7.6 Hz, CH_2_), 6.67 (br. s, 2H, NH_2_), 7.49 (d, 2H, *J* = 8.3 Hz, H_Ar_), 8.09 (d, 2H, *J* = 8.3 Hz, H_Ar_); ^13^C NMR (101 MHz, DMSO-d_6_) δ (ppm): 26.9 (CH_3_), 32.2 (CH_2_), 50.1 (CH_2_), 128.3, 129.9, 135.7, 146.7, 172.5 (C=S), 181.8 (COOH), 197.4 (C=O); Anal. Calcd. for C_12_H_14_N_2_O_3_S: C 54.12; H 5.30; N 10.52%; Found: C 54.25; H 5.27; N 10.39%

### 4.4. General Procedure for the Synthesis of ***5**–**12***

3-[1-(4-Acetylphenyl)thioureido]propanoic acid **4** (0.7 g, 2.6 mmol) was dissolved in acetone (5 mL) at boiling temperature, and corresponding 4′-substituted-2-bromoacetophenone (3.2 mmol) was added. The reaction mixture was heated at reflux for 1–3 h, then it was cooled down and the precipitate was filtered off, and washed with acetone. Obtained hydrobromides were poured with water (30 mL), CH_3_COONa (1.2 g) was added, and stirred at reflux for 5 min. Then, the reaction mixture was cooled, the formed crystalline filtered off, dried, and recrystallized to obtain compounds **5**–**12**.

*3-[(4-Acetylphenyl)(4-phenylthiazol-2-yl)amino]propanoic acid* (**5**): yellow solid, yield 0.85 g (88%); m.p. 149–150 °C (propan-2-ol); IR (KBr) (ν_max_, cm**^−^**^1^): 1665, 1719 (2C=O), 3065 (COOH); ^1^H NMR (400 MHz, DMSO-d_6_) δ (ppm): 2.59 (s, 3H, CH_3_), 2.74 (t, 2H, *J* = 7.1 Hz, CH_2_), 4.29 (t, 2H, *J* = 7.1 Hz, CH_2_), 7.29–7.33 (m, 2H, H_Ar_, CH), 7.40–7.43 (m, 2H, H_Ar_), 7.66 (d, 2H, *J* = 8.3 Hz, H_Ar_), 7.88 (d, 2H, *J* = 7.8 Hz, H_Ar_), 8.08 (d, 2H, *J* = 8.4 Hz, H_Ar_), 12.40 (s, 1H, OH); ^13^C NMR (101 MHz, DMSO-d_6_) δ (ppm): 27.2 (CH_3_), 32.8 (CH_2_), 49.2 (NCH_2_), 104.5 (CH=C), 124.8, 126.2, 128.2, 129.1, 130.4, 134.4, 134.8, 149.0 (C_Ar_), 150.8 (CH=C), 167.6 (C=N), 173.0 (COOH), 197.2 (CH_3_C=O); Anal. Calcd. for C_20_H_18_N_2_O_3_S: C 65.56; H 4.95; N 7.65%; Found: C 65.38; H 5.19; N 7.41%.

*3-{(4-Acetylphenyl)[4-(4-chlorophenyl)thiazol-2-yl]amino}propanoic acid* (**6**): bright yellow solid, yield 0.93 g (89%); m.p. 160–161 °C (propan-2-ol); IR (KBr) (ν_max_, cm**^−^**^1^): 1657, 1727 (2C=O), 3091 (COOH); ^1^H NMR (400 MHz, DMSO-d_6_) δ (ppm): 2.59 (s, 3H, CH_3_), 2.70 (t, 2H, *J* = 7.1 Hz, CH_2_), 4.27 (t, 2H, *J* = 7.1 Hz, CH_2_), 7.39 (s, 1H, CH), 7.47 (d, 2H, *J* = 8.2 Hz, H_Ar_), 7.65 (d, 2H, *J* = 8.3 Hz, H_Ar_), 7.89 (d, 2H, *J* = 8.2 Hz, H_Ar_), 8.03 (d, 2H, *J* = 8.3 Hz, H_Ar_); ^13^C NMR (101 MHz, DMSO-d_6_) δ (ppm): 27.2 (CH_3_), 33.0 (CH_2_), 49.3 (NCH_2_), 105.2 (CH=C), 124.9, 127.9, 129.1, 130.4, 132.6, 133.7, 134.6, 148.9 (C_Ar_), 159.5 (CH=C), 167.8 (C=N), 173.1 (COOH), 197.2 (CH_3_C=O); Anal. Calcd. for C_20_H_17_ClN_2_O_3_S: C 59.92; H 4.27; N 6.99%; Found: C 59.93; H 4.43; N 6.99%.

*3-[(4-Acetylphenyl)[4-(4-nitrophenyl)thiazol-2-yl]amino}propanoic acid* (**7**): bright yellow solid, yield 0.91 g (85%); m.p. 179–180 °C (propan-2-ol); IR (KBr) (ν_max_, cm**^−^**^1^): 1318, 1499 (NO_2_), 1671, 1699 (2C=O), 3047 (COOH); ^1^H NMR (400 MHz, DMSO-d_6_) δ (ppm): 2.60 (s, 3H, CH_3_), 2.73 (t, 2H, *J* = 7.0 Hz, CH_2_), 4.30 (t, 2H, *J* = 7.0 Hz, CH_2_), 7.66–7.68 (m, 3H, H_Ar_, CH), 8.05 (d, 2H, *J* = 8.1 Hz, H_Ar_), 8.13 (d, 2H, *J* = 7.6 Hz, H_Ar_), 8.27 (d, 2H, *J* = 7.3 Hz, H_Ar_), 12.38 (br. s, 1H, OH); ^13^C NMR (101 MHz, DMSO-d_6_) δ (ppm): 27.2 (CH_3_), 32.7 (CH_2_), 49.2 (NCH_2_), 109.0 (CH=C), 124.6, 125.4, 127.0, 130.5, 134.9, 140.8, 146.8, 148.7 (C_Ar_), 148.7 (CH=C), 168.2 (C=N), 173.0 (COOH), 197.3 (CH_3_C=O); Anal. Calcd. for C_20_H_17_N_3_O_5_S: C 58.39; H 4.16; N 10.21%; Found: C 58.59; H 4.24; N 10.45%.

*3-{(4-Acetylphenyl)[4-(4-cyanophenyl)thiazol-2-yl]amino}propanoic acid* (**8**): beige solid, yield 0.79 g (78%); m.p. 171–172 °C (propan-2-ol); IR (KBr) (ν_max_, cm**^−^**^1^): 1270 (CN), 1675, 1713 (2C=O), 3060 (COOH); ^1^H NMR (400 MHz, DMSO-d_6_) δ (ppm): 2.60 (s, 3H, CH_3_), 2.73 (t, 2H, *J* = 7.1 Hz, CH_2_), 4.29 (t, 2H, *J* = 7.0 Hz, CH_2_), 7.62 (s, 1H, CH), 7.66 (d, 2H, *J* = 8.3 Hz, H_Ar_), 7.88 (d, 2H, *J* = 8.0 Hz, H_Ar_), 8.05 (t, 4H, *J* = 7.1 Hz, H_Ar_); ^13^C NMR (101 MHz, DMSO-d_6_) δ (ppm): 27.2 (CH_3_), 32.7 (CH_2_), 49.2 (NCH_2_), 108.0 (CH=C), 110.2 (C≡N), 119.5, 125.3, 126.8, 130.5, 133.2, 134.8, 138.9, 148.7 (C_Ar_), 149.0 (CH=C), 168.1 (C=N), 173.0 (COOH), 197.3 (CH_3_C=O); Anal. Calcd. for C_21_H_17_N_3_O_3_S: C 64.44; H 4.38; N 10.73%; Found: C 64.04; H 4.54; N 10.65%.

*3-{(4-Acetylphenyl)[4-(4-fluorophenyl)thiazol-2-yl]amino}propanoic acid* (**9**): beige solid, yield 1.71 g (72%); m.p. 152–153 °C (propan-2-ol); IR (KBr) (ν_max_, cm**^−^**^1^): 1677, 1712 (2C=O), 3069 (COOH); ^1^H NMR (400 MHz, DMSO-d_6_) δ (ppm): 2.59 (s, 3H, CH_3_), 2.73 (t, 2H, *J* = 7.0 Hz, CH_2_), 4.28 (t, 2H, *J* = 7.0 Hz, CH_2_), 7.24 (t, 2H, *J* = 8.8 Hz, H_Ar_), 7.30 (s, 1H, CH), 7.65 (d, 2H, *J* = 8.5 Hz, H_Ar_), 7.92 (dd, 2H, *J* = 8.0, 5.9 Hz, H_Ar_), 8.03 (d, 2H, *J* = 8.5 Hz, H_Ar_), 12.35 (s, 1H, OH); ^13^C NMR (101 MHz, DMSO-d_6_) δ (ppm): 27.1 (CH_3_), 32.8 (CH_2_), 49.1 (NCH_2_), 104.2 (CH=C), 115.9 (d, *J* = 21.5 Hz, C-3,5), 124.9, 128.2 (d, *J* = 8.1 Hz, C-2,6), 130.4, 131.5 (d, *J* = 3.0 Hz, C-1), 134.5, 148.9 (C_Ar_), 149.7 (CH=C), 162.2 (d, *J* = 244.6 Hz, C–F), 167.7 (C=N), 173.0 (COOH), 197.2 (CH_3_C=O); Anal. Calcd. for C_20_H_17_FN_2_O_3_S: C 62.49; H 4.46; N 7.29%; Found: C 62.63; H 4.48; N 7.71%.

*3-/(4-Acetylphenyl){4-[4-(trifluoromethyl)phenyl]thiazol-2-yl}amino/propanoic acid* (**10**): ivory solid, yield 0.50 g (46%); m.p. 163–164 °C (propan-2-ol); IR (KBr) (ν_max_, cm**^−^**^1^): 1677, 1709 (2C=O), 3056 (COOH); ^1^H NMR (400 MHz, DMSO-d_6_) δ (ppm): 2.60 (s, 3H, CH_3_), 2.73 (t, 2H, *J* = 7.0 Hz, CH_2_), 4.29 (t, 2H, *J* = 7.0 Hz, CH_2_), 7.55 (s, 1H, CH), 7.67 (d, 2H, *J* = 8.4 Hz, H_Ar_), 7.77 (d, 2H, *J* = 8.1 Hz, H_Ar_), 8.03–8.10 (m, 4H, H_Ar_), 12.43 (s, 1H, OH); ^13^C NMR (101 MHz, DMSO-d_6_) δ (ppm): 27.2 (CH_3_), 32.8 (CH_2_), 49.2 (NCH_2_), 107.1 (CH=C), 123.5, 125.2, 126.1 (q, *J* = 271.6 Hz, CF_3_), 126.7, 128.2 (d, *J* = 31.6 Hz, C-3,5), 130.5, 134.7, 138.5, 148.8 (C_Ar_), 149.2 (CH=C), 168.0 (C=N), 173.0 (COOH), 197.0 (CH_3_C=O); Anal. Calcd. for C_21_H_17_F_3_N_2_O_3_S: C 58.06; H 3.94; N 6.45%; Found: C 58.29; H 4.16; N 6.65%.

*3-{(4-Acetylphenyl)[4-(4-hydroxyphenyl)thiazol-2-yl]amino}propanoic acid* (**11**): bright yellow solid, yield 0.79 g (80%); m.p. 174–175 °C (propan-2-ol); IR (KBr) (ν_max_, cm**^−^**^1^): 1630, 1727 (2C=O), 3085 (COOH), 3228 (OH); ^1^H NMR (400 MHz, DMSO-d_6_) δ (ppm): 2.59 (s, 3H, CH_3_), 2.72 (t, 2H, *J* = 7.0 Hz, CH_2_), 4.26 (t, 2H, *J* = 7.0 Hz, CH_2_), 6.79 (d, 2H, *J* = 8.3 Hz, H_Ar_), 7.06 (s, 1H, CH), 7.64 (d, 2H, *J* = 8.3 Hz, H_Ar_), 7.69 (d, 2H, *J* = 8.3 Hz, H_Ar_), 8.06 (d, 2H, *J* = 8.3 Hz, H_Ar_), 9.56 (br. s 1H, OH), 12.36 (s, 1H, COOH); ^13^C NMR (101 MHz, DMSO-d_6_) δ (ppm): 27.1 (CH_3_), 32.8 (CH_2_), 49.1 (NCH_2_), 101.8 (CH=C), 115.8, 124.5, 126.2, 127.6, 130.4, 134.2 (C_Ar_), 149.1 (CH=C), 151.1, 157.7 (C_Ar_), 167.3 (C=N), 173.0 (COOH), 197.2 (CH_3_C=O); Anal. Calcd. for C_20_H_18_N_2_O_4_S: C 62.81; H 4.74; N 7.33%; Found: C 62.69; H 4.82; N 7.49%.

*3-/(4-Acetylphenyl){4-[4-(trifluoromethoxy)phenyl]thiazol-2-yl}amino/propanoic acid* (**12**): light yellow solid, yield 0.96 g (82%); m.p. 138–139 °C (propan-2-ol/H_2_O); IR (KBr) (ν_max_, cm**^−^**^1^): 568, 785, 857 (OCF_3_), 1676, 1693 (2C=O), 3004 (COOH); ^1^H NMR (400 MHz, DMSO-d_6_) δ (ppm): 2.59 (s, 3H, CH_3_), 2.73 (t, 2H, *J* = 7.1 Hz, CH_2_), 4.28 (t, 2H, *J* = 7.0 Hz, CH_2_), 7.40–7.42 (m, 3H, CH, H_Ar_), 7.99 (d, 2H, *J* = 8.6 Hz, H_Ar_), 8.04 (d, 4H, *J* = 8.5 Hz, H_Ar_), 12.34 (s, 1H, OH); ^13^C NMR (101 MHz, DMSO-d_6_) δ (ppm): 27.2 (CH_3_), 32.7 (CH_2_), 49.2 (NCH_2_), 105.4 (CH=C), 121.7 (OCF_3_), 121.9, 125.0, 127.9, 130.5, 134.1, 134.6, 148.1, 148.9 (C_Ar_), 149.3 (CH=C), 167.9 (C=N), 173.0 (COOH), 197.2 (CH_3_C=O); Anal. Calcd. for C_21_H_17_F_3_N_2_O_4_S: C 56.00; H 3.80; N 6.22%; Found: C 55.89; H 3.89; N 6.03%.

### 4.5. General Procedure for the Synthesis of ***13**–**17***

[(Thiazol-2-yl)amino]propanoic acid **5** or **6** (5 mmol) was dissolved in acetone (7 mL) at boiling temperature, then corresponding benzaldehyde (2.5 mmol) was added to the mixture along with 2 drops of concentrated HCl. Reaction mixture was heated at reflux for 5 h, then it was cooled down, and the precipitate was filtered off, and washed with acetone. Obtained hydrobromides were poured with water (10 mL), CH_3_COONa (0.5 g) was added, stirred at reflux for 5 min. The formed crystalline was filtered off, dried, and recrystallized to obtain bisphenylthiazoles **13**–**17**.

*3,3′-{[(Phenylmethylene)bis(4-phenylthiazole-2,5-diyl)]bis[(4-acetylphenyl)azanediyl]}dipropionic acid* (**13**): ivory solid, yield 2.4 g (59%); m.p. 198–199 °C (methanol); IR (KBr) (ν_max_, cm**^−^**^1^): 1679, 1725 (4C=O), 3064 (2COOH); ^1^H NMR (400 MHz, DMSO-d_6_) δ (ppm): 2.57 (s, 6H, 2CH_3_), 2.67 (t, 4H, *J* = 7.0 Hz, 2CH_2_), 4.20 (t, 4H, *J* = 6.9 Hz, 2CH_2_), 5.85 (s, 1H, CH), 7.15–7.36 (m, 15H, H_Ar_), 7.60 (d, 4H, *J* = 8.3 Hz, H_Ar_), 7.98 (d, 4H, *J* = 8.3 Hz, H_Ar_), 12.34 (s, 2H, 2OH); ^13^C NMR (101 MHz, DMSO-d_6_) δ (ppm): 27.1 (2CH_3_), 32.7 (2CH_2_), 41.8 (CH), 49.8 (2NCH_2_), 125.3 (2C=C), 125.4, 127.9, 128.4, 128.4, 128.8, 129.4, 130.4, 134.8, 134.8, 143.8, 147.8 (2C–N), 148.7, 152.8 (C_Ar_), 165.7 (2C=N), 172.9 (2COOH), 197.2 (2CH_3_C=O); Anal. Calcd. for C_47_H_40_N_4_O_6_S_2_: C 68.76; H 4.91; N 6.82%; Found: C 68.85; H 5.05; N 6.58%.

*3,3′-/{[(4-Fluorophenyl)methylene]bis(4-phenylthiazole-5,2-diyl)}bis[(4 acetylphenyl)azanediyl]/dipropionic acid* (**14**): pastel green solid, yield 4.11 g (98%); m.p. 192–193 °C (methanol); IR (KBr) (ν_max_, cm**^−^**^1^): 1678, 1725 (4C=O), 3057 (2COOH); ^1^H NMR (400 MHz, DMSO-d_6_) δ (ppm): 2.56 (s, 6H, 2CH_3_), 2.62 (t, 4H, *J* = 6.7 Hz, 2CH_2_), 4.10–4.24 (m, 4H, 2CH_2_), 5.89 (s, 1H, CH), 7.11–7.33 (m, 14H, H_Ar_), 7.63 (d, 4H, *J* = 8.1 Hz, H_Ar_), 7.97 (d, 4H, *J* = 8.1 Hz, H_Ar_); ^13^C NMR (101 MHz, DMSO-d_6_) δ (ppm): 27.1 (2CH_3_), 32.9 (2CH_2_), 41.1 (CH), 49.9 (2NCH_2_), 116.2 (d, *J* = 21.7 Hz, C-3,5), 125.2, 125.3, 128.4 (2C=C), 128.8, 130.0 (d, *J* = 8.12 Hz, C-2,6), 130.4, 134.7 (d, *J* = 4.2 Hz, C-1), 139.9, 147.9 (2C–N), 148.6 (C_Ar_), 161.6 (d, *J* = 244.9 Hz, C–F), 165.7 (2C=N), 173.0 (2COOH), 197.2 (2CH_3_C=O); Anal. Calcd. for C_47_H_39_FN_4_O_6_S_2_: C 67.29; H 4.69; N 6.68%; Found: C 66.95; H 4.32; N 6.86%.

*3,3′-/{[(4-Chlorophenyl)methylene]bis(4-phenylthiazole-5,2-diyl)}bis[(4-acetylphenyl)azanediyl]/dipropionic acid* (**15**): pastel green solid, yield 2.22 g (52%); m.p. 196–197 °C (propan-2-ol); IR (KBr) (ν_max_, cm**^−^**^1^): 1678, 1725 (4C=O), 2920 (2COOH); ^1^H NMR (400 MHz, DMSO-d_6_) δ (ppm): 2.57 (s, 6H, 2CH_3_), 2.66 (t, 4H, *J* = 7.0 Hz, 2CH_2_), 4.20 (t, 4H, *J* = 6.9 Hz, 2CH_2_), 5.85 (s, 1H, CH), 7.20–7.29 (m, 12H, H_Ar_), 7.38 (d, 2H, *J* = 8.2 Hz, H_Ar_), 7.60 (d, 4H, *J* = 8.3 Hz, H_Ar_), 7.99 (d, 4H, *J* = 8.3 Hz, H_Ar_), 12.36 (br. s, 2H, 2OH); ^13^C NMR (101 MHz, DMSO-d_6_) δ (ppm): 27.1 (2CH_3_), 32.8 (2CH_2_), 41.3 (CH), 48.8 (2NCH2), 124.7 (2C=C), 125.3, 128.4, 128.8, 129.4, 129.8, 130.4, 132.4, 134.7, 134.8, 142.7, 148.1, 148.6 (2C–N), 154.0 (C_Ar_), 165.8 (2C=N), 172.9 (2COOH), 197.2 (2CH_3_C=O); Anal. Calcd. for C_47_H_39_ClN_4_O_6_S_2_: C 65.99; H 4.06; N 6.55%; Found: C 65.76; H 4.24; N 6.79%.

*3,3′-/{[(2,5-Dimethoxyphenyl)methylene]bis(4-phenylthiazole-5,2-diyl)}bis[(4-acetylphenyl)azanediyl]/dipropionic acid* (**16**): dark orange solid, yield 2.6 g (59%); m.p. 174–175 °C (propan-2-ol); IR (KBr) (ν_max_, cm**^−^**^1^): 1681, 1710 (4C=O), 2934 (2COOH); ^1^H NMR (400 MHz, DMSO-d_6_) δ (ppm): 2.58 (s, 6H, 2CH_3_), 2.65 (t, 4H, *J* = 7.1 Hz, 2CH_2_), 3.51 (s, 3H, CH_3_), 3.64 (s, 3H, CH_3_), 4.12–4.25 (m, 4H, 2CH_2_), 6.12 (s, 1H, CH), 6.83 (d, 2H, *J* = 8.3 Hz, H_Ar_), 6.95 (d, 1H, *J* = 8.4 Hz, H_Ar_), 7.20–7.27 (m, 10H, H_Ar_), 7.54 (d, 4H, *J* = 8.4 Hz, H_Ar_), 7.99 (d, 4H, *J* = 8.5 Hz, H_Ar_), 12.32 (br. s, 2H, 2OH); ^13^C NMR (101 MHz, DMSO-d_6_) δ (ppm): 27.1 (2CH_3_), 32.7 (2CH_2_), 36.0 (CH), 48.7 (2NCH_2_), 55.7 (PhC4–OCH_3_), 56.8 (PhC_2_–OCH_3_), 112.3, 113.5, 115.2, 125.1, 125.6 (2C=C), 128.2, 128.3, 128.5, 130.4, 133.5, 134.6, 135.0, 147.1, 148.7 (2C–N), 150.6, 153.5 (C_Ar_), 165.0 (2C=N), 172.9 (2COOH), 197.2 (2CH_3_C=O); Anal. Calcd. for C_49_H_44_N_4_O_8_S_2_: C 66.80; H 5.03; N 6.36%; Found: C 67.01; H 5.24; N 6.56%.

*3,3′-/{[(4-Fluorophenyl)methylene]bis[4-(4-chlorophenyl)thiazole-5,2-diyl]}bis](4-acetylphenyl)azanediyl]/dipropionic acid* (**17**): yellow solid, yield 2.67 g (59%); m.p. 193–194 °C (propan-2-ol); IR (KBr) (ν_max_, cm**^−^**^1^): 1679, 1705 (4C=O), 2960 (2COOH); ^1^H NMR (400 MHz, DMSO-d_6_) δ (ppm): 2.57 (s, 6H, 2CH_3_), 2.64 (t, 4H, J = 7.0 Hz, 2CH_2_), 4.17 (t, 4H, J = 6.8 Hz, 2CH_2_), 5.80 (s, 1H, CH), 7.15 (t, 2H, J = 8.4 Hz, H_Ar_), 7.24–7.31 (m, 10H, H_Ar_), 7.58 (d, 4H, J = 8.1 Hz, H_Ar_), 7.98 (d, 4H, J = 8.1 Hz, H_Ar_), 12.00 (br. s, 1H, OH); ^13^C NMR (101 MHz, DMSO-d_6_) δ (ppm): 27.1 (2CH_3_), 32.8 (2CH_2_), 41.0 (CH), 48.9 (2CH_2_), 116.3 (d, J = 20.6 Hz, C-3,5), 125.3 (2C=C), 125.6, 128.8, 130.1, 130.4, 133.2, 133.5, 139.4 (d, J = 3.1 Hz, C-1), 146.7 (C_Ar_), 148.5 (2C–N), 161.7 (d, J = 244.6 Hz, C–F), 165.9 (2C=N), 172.9 (2COOH), 197.2 (2CH_3_C=O); Anal. Calcd. for C_47_H_37_Cl_2_FN_4_O_6_S_2_: C 62.18; H 4.11; N 6.17%; Found: C 62.29; H 4.24; N 6.23%.

### 4.6. General Procedure for the Synthesis of ***18**–**20***

[(Thiazol-2-yl)amino]propanoic acid **5**, **6** or **12** (5 mmol) was dissolved in a 30% KOH solution (6 mL), 4-fluorobenzaldehyde (6 mmol, 0.75 g) was added dropwise. The reaction mixture was heated at 60 °C for 10 h, then it was cooled down and the precipitate was filtered off, and washed with saturated NaCl solution, then it was placed in a beaker, poured with H_2_O (10 mL), heated until dissolved, cooled down, and acidified with diluted (1:2) CH_3_COOH to pH 5. The formed crystalline was filtered off, dried, and recrystallized to obtain compounds **18**–**20**.

*3-/{4-[3-(4-Fluorophenyl)acryloyl]phenyl}(4-phenylthiazol-2-yl)amino/propanoic acid* (**18**): bright orange solid, yield 2.03 g (86%); m.p. 164–165 °C (methanol); IR (KBr) (ν_max_, cm**^−^**^1^): 1732 (C=O), 3268 (COOH); ^1^H NMR (400 MHz, DMSO-d_6_) δ (ppm): *Z/E* 2.77 (t, 2H, *J* = 6.9 Hz, CH_2_), 4.32 (t, 2H, *J* = 6.9 Hz, CH_2_), 7.32 (t, 3H, *J* = 8.2 Hz, H_Ar_), 7.37 (s, 1H, CH=C), 7.42 (t, 2H, *J* = 7.5 Hz, H_Ar_), 7.72 (d, 2H, *J* = 8.3 Hz, H_Ar_), 7.73 (s, 0.42H, CH=CH), 7.76 (s, 0.58H, CH=CH), 7.89 (d, 2H, *J* = 7.6 Hz, H_Ar_), 7.96 (s, 0.46H, CH=CH), 7.99 (s, 0.54H, CH=CH), 8.01 (d, 2H, *J* = 8.7 Hz, H_Ar_), 8.26 (d, 2H, *J* = 7.6 Hz, H_Ar_), 12.37 (s, 1H, OH); ^13^C NMR (101 MHz, DMSO-d_6_) δ (ppm): 32.8 (CH_2_), 49.2 (NCH_2_), 104.7 (CH=C), 116.4 (d, *J* = 21.7 Hz, C-3,5), 122.3 (CH), 124.7, 126.2, 128.2, 129.1, 130.8, 131.8 (d, *J* = 8.6 Hz, C-2,6), 131.9 (d, *J* = 3.0 Hz, C-1), 134.8, 134.9, 143.1 (CH), 149.0 (C_Ar_), 150.8 (CH=C), 163.9 (d, *J* = 248.7 Hz, C–F), 167.5 (C=N), 173.0 (COOH), 188.1 (CHC=O); Anal. Calcd. for C_27_H_21_FN_2_O_3_S: C 68.63; H 4.48; N 5.93%; Found: C 68.13; H 4.51; N 5.99%.

*3-/[4-(4-Chlorophenyl)thiazol-2-yl] {4-[3-(4-fluorophenyl)acryloyl]phenyl}amino/propanoic acid* (**19**): bright yellow solid, yield 2.18 g (86%); m.p. 171–172 °C (methanol); IR (KBr) (ν_max_, cm**^−^**^1^): 1717 (C=O), 2923 (COOH); ^1^H NMR (400 MHz, DMSO-d_6_) δ (ppm): *Z/E* 2.75 (t, 2H, *J* = 7.0 Hz, CH_2_), 4.31 (t, 2H, *J* = 6.9 Hz, CH_2_), 7.32 (t, 2H, *J* = 8.6 Hz, H_Ar_), 7.42 (s, 1H, CH=C), 7.48 (d, 2H, *J* = 8.2 Hz, H_Ar_), 7.71 (d, 2H, *J* = 8.3 Hz, H_Ar_), 7.76 (s, 0.39H, CH=CH), 7.76 (s, 0.61H, CH=CH), 7.91 (d, 2H, *J* = 8.1 Hz, H_Ar_), 7.96 (s, 0.62H, CH=CH), 7.99 (s, 0.38H, CH=CH), 8.01 d, 2H, *J* = 7.8 Hz, H_Ar_), 8.26 (d, 2H, *J* = 8.2 Hz, H_Ar_), 12.36 (s, 1H, OH); ^13^C NMR (101 MHz, DMSO-d_6_) δ (ppm): 32.8 (CH_2_), 49.6 (NCH_2_), 105.3 (CH=C), 116.4 (d, *J* = 21.8 Hz, C-3,5), 122.3 (CH), 124.9, 127.9, 129.1, 130.8, 131.8 (d, *J* = 8.4 Hz, C-2,6), 131.9 (d, *J* = 3.0 Hz, C-1), 132.6, 133.7, 135.2, 143.2 (CH), 148.9 (C_Ar_), 149.5 (CH=C), 162.7 (d, *J* = 249.8 Hz, C–F), 167.8 (C=N), 173.0 (COOH), 188.2 (CHC=O); Anal. Calcd. for C_27_H_20_ClFN_2_O_3_S: C 63.97; H 3.98; N 5.53%; Found: C 63.79; H 4.02; N 5.79%.

*3-/{4-[3-(4-Fluorophenyl)acryloyl]phenyl}{4-[4-(trifluoromethoxy)phenyl]thiazol-2-yl}amino/propanoic acid* (**20**): yellow solid, yield 2.28 g (82%); m.p. 171–172 °C (methanol); IR (KBr) (ν_max_, cm**^−^**^1^): 1719 (C=O), 2889 (COOH); ^1^H NMR (400 MHz, DMSO-d_6_) δ (ppm): *Z/E* 2.76 (t, 2H, *J* = 7.0 Hz, CH_2_), 4.31 (t, 2H, *J* = 6.5 Hz, CH_2_), 7.31 (t, 2H, *J* = 8.6 Hz, H_Ar_), 7.29–7.43 (m, 3H, CH=C, H_Ar_), 7.71 (d, 2H, *J* = 8.6 Hz, H_Ar_), 7.76 (s, 0.38H, CH=CH), 7.79 (s, 0.62H, CH=CH), 7.95 (s, 0.68H, CH=CH), 7.97–8.03 (m, 4H, H_Ar_+ 0.32H, CH=CH), 8.26 (d, 2H, *J* = 8.4 Hz, H_Ar_), 12.43 (s, 1H, OH); ^13^C NMR (101 MHz, DMSO-d_6_) δ (ppm): 33.9 (CH_2_), 49.3 (NCH_2_), 105.6 (CH=C), 116.4 (d, *J* = 21.8 Hz, C-3,5), 119.3, 121.8 (OCF_3_), 122.8 (CH), 124.9, 128.0, 130.8 (CH), 131.8 (d, *J* = 8.6 Hz, C-2,6), 131.9 (d, *J* = 3.2 Hz, C-1), 134.1, 135.2, 143.2, 148.9 (C_Ar_), 149.4 (CH=C), 163.9 (d, *J* = 248.9 Hz, C–F), 167.8 (C=N), 173.2 (COOH), 188.1 (CHC=O); Anal. Calcd. for C_28_H_20_F_4_N_2_O_4_S: C 60.43; H 3.62; N 5.03%; Found: C 60.13; H 3.68; N 5.16%.

### 4.7. General Procedure for the Synthesis of Oximes ***21**, **22***

A mixture of corresponding thiazole derivative **5** or **6** (1 mmol), hydroxylamine hydrochloride (4 mmol, 0.28 g), and sodium acetate (4 mmol, 0.33 g) were heated at reflux in propan-2-ol (6 mL) for 1 h. Then, the solvent was evaporated under reduced pressure, the residue was poured with water, and the obtained crystals were filtered off, washed with water, and recrystallized.

*3-/{4-[1-(Hydroxyimino)ethyl]phenyl}(4-phenylthiazol-2-yl)amino/propanoic acid* (**21**): yellowish solid, yield 0.37 g (97%); m.p. 195–196 °C (propan-2-ol); IR (KBr) (ν_max_, cm**^−^**^1^): 1600 (C=N), 1699 (C=O), 3126 (N–OH), 3226 (COOH); ^1^H NMR (400 MHz, DMSO-d_6_) δ (ppm): 2.18 (s, 3H, CH_3_), 2.71 (t, 2H, *J* = 7.1 Hz, CH_2_), 4.22 (t, 2H, *J* = 7.1 Hz, CH_2_), 7.20 (s, 1H, CH), 7.30 (t, 1H, *J* = 7.3 Hz, H_Ar_), 7.41 (t, 2H, *J* = 7.5 Hz, H_Ar_), 7.50 (d, 2H, *J* = 8.4 Hz, H_Ar_), 7.76 (d, 2H, *J* = 8.4 Hz, H_Ar_), 7.86 (d, 2H, *J* = 7.6 Hz, H_Ar_), 11.30 (s, 1H, OH), 12.31 (br. s, 1H, OH); ^13^C NMR (101 MHz, DMSO-d_6_) δ (ppm): 11.9 (CH_3_), 32.8 (CH_2_), 49.1 (NCH_2_), 103.5 (CH=C), 126.1, 126.7, 127.5, 128.1, 129.1, 135.0, 136.0, 145.2 (C_Ar_), 150.8 (CH=C), 152.8 (C=NOH), 167.8 (C=N), 173.1 (C=O); Anal. Calcd. for C_20_H_19_N_3_O_3_S: C 62.98; H 5.02; N 11.02%; Found: C 62.76; H 5.03; N 11.26%.

*3-/[4-(4-Chlorophenyl)thiazol-2-yl]{4-[1-(hydroxyimino)ethyl]phenyl}amino/propanoic acid* (**22**): white solid, yield 0.41 g (98%); m.p. 198–199 °C (propan-2-ol); IR (KBr) (ν_max_, cm**^−^**^1^): 1698 (C=O), 3124 (N–OH), 3234 (COOH); ^1^H NMR (400 MHz, DMSO-d_6_) δ (ppm): 2.16 (s, 3H, CH_3_), 2.70 (t, 2H, *J* = 7.1 Hz, CH_2_), 4.22 (t, 2H, *J* = 7.1 Hz, CH_2_), 7.26 (s, 1H, CH), 7.47 (d, 2H, *J* = 8.8 Hz, H_Ar_), 7.49 (d, 2H, *J* = 8.8 Hz, H_Ar_), 7.76 (d, 2H, *J* = 8.1 Hz, H_Ar_), 7.89 (d, 2H, *J* = 8.1 Hz, H_Ar_), 11.30 (s, 1H, OH), 12.31 (br. s, 1H, OH); ^13^C NMR (101 MHz, DMSO-d_6_) δ (ppm): 11.9 (CH_3_), 32.8 (CH_2_), 49.0 (NCH_2_), 104.2 (CH=C), 126.8, 127.6, 127.8, 129.1, 132.4, 133.9, 136.1, 145.1 (C_Ar_), 149.6 (CH=C), 152.8 (C=NOH), 169.0 (C=N), 173.1 (C=O); Anal. Calcd. for C_20_H_18_ClN_3_O_3_S: C 57.76; H 4.36; N 10.10%; Found: C 57.89; H 4.47; N 10.45%.

### 4.8. General Procedure for the Synthesis of Esters ***23**, **24***

To a solution of the corresponding carboxylic acid **21**, **22** (1 mmol) in methanol (10 mL), concentrated sulfuric acid (1 mL) was added dropwise and the mixture was heated at reflux for 4 h. Then, the solvent was evaporated under reduced pressure, and the residue neutralized with 5% sodium carbonate solution to pH 7. The obtained solid was filtered off, washed with plenty of water, and recrystallized from isopropyl alcohol to give the title compounds **23**, **24**.

*Methyl 3-/{4-[1-(hydroxyimino)ethyl]phenyl}(4-phenylthiazol-2-yl)amino/propanoate* (**23**): light beige solid, yield 0.32 g (81%); m.p. 146–147 °C (propan-2-ol); IR (KBr) (ν_max_, cm**^−^**^1^): 1720 (C=O), 3121 (N–OH), 3213 (COOH); ^1^H NMR (400 MHz, DMSO-d_6_) δ (ppm): 2.18 (s, 3H, CH_3_), 2.79 (t, 2H, *J* = 6.8 Hz, CH_2_), 3.52 (s, 3H, OCH_3_) 4.26 (t, 2H, *J* = 6.8 Hz, CH_2_), 7.19 (s, 1H, CH), 7.30 (t, 2H, *J* = 7.3 Hz, H_Ar_), 7.41 (t, 1H, *J* = 7.5 Hz, H_Ar_), 7.49 (d, 2H, *J* = 8.4 Hz, H_Ar_), 7.76 (d, 2H, *J* = 8.4 Hz, H_Ar_), 7.87 (d, 2H, *J* = 8.4 Hz, H_Ar_), 11.31 (s, 1H, OH); ^13^C NMR (101 MHz, DMSO-d_6_) δ (ppm): 11.9 (CH_3_), 32.7 (CH_2_), 49.1 (NCH_2_), 51.9 (CH_3_), 103.5 (CH=C), 126.1, 126.7, 127.6, 128.1, 129.0, 135.0, 136.1, 145.1 (C_Ar_), 150.8 (CH=C), 152.8 (C=NOH), 168.8 (C=N), 172.0 (C=O); Anal. Calcd. for C_21_H_21_N_3_O_3_S: C 63.78; H 5.35; N 10.63%; Found: C 63.47; H 5.44; N 10.93%.

*Methyl 3-/[4-(4-chlorophenyl)thiazol-2-yl]{4-[1-(hydroxyimino)ethyl]phenyl}amino/propanoate* (**24**): yellow solid, yield 0.4 g (95%); m.p. 162–163 °C (propan-2-ol); IR (KBr) (ν_max_, cm**^−^**^1^): 1733 (C=O), 3130 (N–OH), 3225 (COOH); ^1^H NMR (400 MHz, DMSO-d_6_) δ (ppm): 2.18 (s, 3H, CH_3_), 2.78 (t, 2H, *J* = 6.8 Hz, CH_2_), 3.51 (s, 3H, OCH_3_) 4.25 (t, 2H, *J* = 6.8 Hz, CH_2_), 7.26 (s, 1H, CH), 7.45–7.49 (m, 4H, H_Ar_), 7.76 (d, 2H, *J* = 8.5 Hz, H_Ar_), 7.89 (d, 2H, *J* = 8.5 Hz, H_Ar_), 11.31 (s, 1H, OH); ^13^C NMR (101 MHz, DMSO-d_6_) δ (ppm): 11.9 (CH_3_), 32.7 (CH_2_), 49.0 (NCH_2_), 51.9 (CH_3_), 104.3 (CH=C), 126.8, 127.6, 127.8, 129.1, 132.5, 133.8, 136.2, 145.0 (C_Ar_), 149.5 (CH=C), 152.7 (C=NOH), 169.0 (C=N), 172.0 (C=O); Anal. Calcd. for C_21_H_20_ClN_3_O_3_S: C 58.67; H 4.69; N 9.77%; Found: C 58.86; H 4.79; N 9.95%.

### 4.9. General Procedure for the Preparation of Hydrazides ***25**, **26***

A mixture of the corresponding methyl ester **23**, **24** (5 mmol), hydrazine monohydrate (2.5 mmol, 1.25 g, 1.21 mL) and propan-2-ol (6 mL) was heated at reflux for 3 h. After completion of the reaction (TLC), the mixture was cooled to room temperature, the formed precipitate filtered off, washed with isopropyl alcohol, and recrystallized to give the title compound **25**, **26**.

*3-/{4-[1-(Hydroxyimino)ethyl]phenyl}(4-phenylthiazol-2-yl)amino/propanehydrazide* (**25**): yellow solid, yield 1.28 g (65%); m.p. 169–170 °C (propan-2-ol); IR (KBr) (ν_max_, cm**^−^**^1^): 3124 (N–OH), 3285 (NHNH_2_); ^1^H NMR (400 MHz, DMSO-d_6_) δ (ppm): 2.18 (s, 3H, CH_3_), 2.54 (d, 2H, *J* = 7.2 Hz, CH_2_), 4.15–4.36 (m, 4H, CH_2_, NH_2_), 7.15 (s, 1H, CH), 7.30 (t, 1H, *J* = 7.43 Hz, H_Ar_), 7.41 (t, 2H, *J* = 7.5 Hz, H_Ar_), 7.49 (d, 2H, *J* = 8.5 Hz, H_Ar_), 7.75 (d, 2H, *J* = 8.5 Hz, H_Ar_), 7.89 (d, 2H, *J* = 7.4 Hz, H_Ar_), 9.10 (s, 1H, NH), 11.29 (s, 1H, OH); ^13^C NMR (101 MHz, DMSO-d_6_) δ (ppm): 11.9 (CH_3_), 32.4 (CH_2_), 49.8 (NCH_2_), 103.4 (CH=C), 126.2, 126.5, 127.5, 128.0, 129.0, 135.1, 135.8, 145.3 (C_Ar_), 150.9 (CH=C), 152.8 (C=NOH), 168.7 (C=N), 169.8 (C=O); Anal. Calcd. for C_20_H_21_N_5_O_2_S: C 60.74; H 5.35; N 17.71%; Found: C 60.62; H 5.49; N 17.71%.

*3-/[4-(4-Chlorophenyl)thiazol-2-yl]{4-[1-(hydroxyimino)ethyl]phenyl}amino/propanehydrazide* (**26**): light yellow solid, yield 1.59 g (74%); m.p. 147–148 °C (propan-2-ol); IR (KBr) (ν_max_, cm**^−^**^1^): 3126 (N–OH), 3296 (NHNH_2_); ^1^H NMR (400 MHz, DMSO-d_6_) δ (ppm): 2.18 (s, 3H, CH_3_), 2.53 (s, 2H, CH_2_), 4.14–4.25 (m, 4H, CH_2_, NH_2_), 7.26 (s, 1H, CH), 7.45–7.49 (m, 4H, H_Ar_), 7.75 (d, 2H, *J* = 8.02 Hz, H_Ar_), 7.90 (d, 2H, *J* = 8.1 Hz, H_Ar_), 9.09 (s, 1H, NH), 11.29 (s, 1H, OH); ^13^C NMR (101 MHz, DMSO-d_6_) δ (ppm): 11.9 (CH_3_), 32.4 (CH_2_), 49.9 (NCH_2_), 104.2 (CH=C), 126.7, 127.5, 127.9, 129.0, 132.5, 134.0, 135.9, 145.2 (C_Ar_), 149.6 (CH=C), 152.7 (C=NOH), 168.9 (C=N), 169.8 (C=O); Anal. Calcd. for C_20_H_20_ClN_5_O_2_S: C 55.88; H 4.69; N 16.29%; Found: C 55.92; H 4.36; N 16.28%.

### 4.10. General Procedure for the Preparation of Compounds ***27**, **28***

Compound **5** or **6** (7 mmol) was dissolved in propan-2-ol, phenylhydrazine (10.5 mmol, 1.14 g, 1 mL) was added dropwise, and the mixture was stirred at boiling temperature for 2 h. Then, it was cooled down, formed crystals were filtered off, washed with propan-2-ol, and dried.

*3-/{4-[1-(2-Phenylhydrazineylidene)ethyl]phenyl}(4-phenylthiazol-2-yl)amino/propanoic acid* (**27**): dark orange solid, yield 2.74 g (86%); m.p. 84–85 °C (methanol); IR (KBr) (ν_max_, cm**^−^**^1^): 1599 (C=N), 1708 (C=O), 2923 (COOH), 3108 (NH); ^1^H NMR (400 MHz, DMSO-d_6_) δ (ppm): 2.29 (s, 3H, CH_3_), 2.73 (t, 2H, *J* = 7.1 Hz, CH_2_), 4.24 (t, 2H, *J* = 7.0 Hz, CH_2_), 6.77 (t, 1H, *J* = 6.5 Hz, H_Ar_), 7.18 (s, 1H, CH), 7.19–7.34 (m, 5H, H_Ar_), 7.41 (t, 2H, *J* = 7.4 Hz, H_Ar_), 7.48 (d, 2H, *J* = 8.0 Hz, H_Ar_), 7.85–7.93 (m, 4H, H_Ar_), 9.35 (s, 1H, NH), 12.32 (s, 1H, OH); ^13^C NMR (101 MHz, DMSO-d_6_) δ (ppm): 13.2 (CH_3_), 32.9 (CH_2_), 49.0 (NCH_2_), 103.4 (CH=C), 113.3, 119.5, 126.2, 126.8, 127.1, 128.0, 129.0, 129.4, 135.1, 138.5, 140.1, 144.1 (C_Ar_), 146.4 (CH=C), 150.8 (C=NNH), 169.1 (C=N), 173.1 (C=O); Anal. Calcd. for C_26_H_24_N_4_O_2_S: C 68.40; H 5.30; N 12.27%; Found: C 68.36; H 5.29; N 12.87%.

*3-/[4-(4-Chlorophenyl)thiazol-2-yl]{4-[1-(2-phenylhydrazineylidene)ethyl]phenyl}amino/propanoic acid* (**28**): pink gold solid, yield 2.47 g (72%); m.p. 149–150 °C (propan-2-ol); IR (KBr) (ν_max_, cm**^−^**^1^): 1601 (C=N), 1699 (C=O), 2928 (COOH), 3104 (NH); ^1^H NMR (400 MHz, DMSO-d_6_) δ (ppm): 2.28 (s, 3H, CH_3_), 2.71 (t, 2H, *J* = 7.0 Hz, CH_2_), 4.23 (t, 2H, *J* = 6.9 Hz, CH_2_), 6.77 (t, 1H, *J* = 6.6 Hz, H_Ar_), 7.21–7.26 (m, 5H, H_Ar_, CH), 7.40–7.52 (m, 4H, H_Ar_), 7.89 (d, 4H, *J* = 7.7 Hz, H_Ar_), 9.35 (s, 1H, NH), 12.31 (s, 1H, OH); ^13^C NMR (101 MHz, DMSO-d_6_) δ (ppm): 13.2 (CH_3_), 32.8 (CH_2_), 49.0 (NCH_2_), 104.1 (CH=C), 113.3, 119.5, 126.9, 127.1, 127.8, 129.1, 129.4, 132.4, 133.9, 138.6, 140.1, 144.0 (C_Ar_), 146.4 (CH=C), 149.6 (C=NNH), 169.3 (C=N), 173.1 (C=O); Anal. Calcd. for C_26_H_23_ClN_4_O_2_S: C 63.60; H 4.72; N 11.41%; Found: C 63.45; H 4.75; N 11.11%.

### 4.11. General Procedure for the Preparation of Compounds ***29**, **30***

To a solution of the corresponding propanoic acid **5**, **6** (2 mmol) in methanol (50 mL), concentrated sulfuric acid (1 mL) was added dropwise and the mixture was heated at reflux for 7 h. Then, the solvent was evaporated under reduced pressure, and the residue neutralized with 5% sodium carbonate solution to pH 7. The obtained solid was filtered off, washed with plenty of water, and recrystallized from isopropyl alcohol to give the title compounds **29**, **30**.

*Methyl 3-[(4-acetylphenyl)(4-phenylthiazol-2-yl)amino]propanoate* (**29**): light yellow solid, yield 0.7 g (92%); m.p. 72–73 °C (propan-2-ol); IR (KBr) (ν_max_, cm**^−^**^1^): 1680, 1733 (2C=O); ^1^H NMR (400 MHz, DMSO-d6) δ (ppm): 2.60 (s, 3H, CH_3_), 2.82 (t, 2H, J = 6.5 Hz, CH_2_), 3.52 (s, 3H, OCH_3_), 4.33 (t, 2H, *J* = 6.5 Hz, CH_2_), 7.28–7.36 (m, 2H, H_Ar_, CH), 7.42 (t, 2H, *J* = 7.3 Hz, H_Ar_), 7.64 (d, 2H, *J* = 8.0 Hz, H_Ar_), 7.88 (d, 2H, *J* = 7.4 Hz, H_Ar_), 8.04 (d, 2H, *J* = 8.0 Hz, H_Ar_); ^13^C NMR (101 MHz, DMSO-d_6_) δ (ppm): 27.2 (CH_3_), 32.7 (CH_2_), 49.1 (NCH_2_), 51.9 (CH_3_), 104.5 (CH=C), 125.0, 126.2, 128.2, 129.1, 130.5, 134.6, 134.8, 148.9 (C_Ar_), 150.8 (CH=C), 167.6 (C=N), 171.9 (COOH), 197.2 (CH_3_C=O); Anal. Calcd. for C_21_H_20_N_2_O_3_S: C 66.30; H 5.30; N 7.36%; Found: C 66.12; H 5.36; N 7.56%.

*Methyl 3-{(4-acetylphenyl)[4-(4-chlorophenyl)thiazol-2-yl]amino}propanoate* (**30**): light brown solid, yield 0.78 g (95%); m.p. 115–116 °C (propan-2-ol); IR (KBr) (ν_max_, cm**^−^**^1^): 1671, 1724 (2C=O); ^1^H NMR (400 MHz, DMSO-d_6_) δ (ppm): 2.60 (s, 3H, CH_3_), 2.81 (t, 2H, *J* = 6.8 Hz, CH_2_), 3.51 (s, 3H, OCH_3_), 4.32 (t, 2H, *J* = 6.8 Hz, CH_2_), 7.38 (s, 1H, CH), 7.47 (d, 2H, *J* = 8.2 Hz, H_Ar_), 7.64 (d, 2H, *J* = 8.2 Hz, H_Ar_), 7.89 (d, 2H, *J* = 8.2 Hz, H_Ar_), 8.04 (d, 2H, *J* = 8.2 Hz, H_Ar_); ^13^C NMR (101 MHz, DMSO-d_6_) δ (ppm): 27.2 (CH_3_), 32.6 (CH_2_), 49.1 (NCH_2_), 51.9 (CH_3_), 105.2 (CH=C), 125.2, 127.9, 129.1, 130.5, 132.6, 133.7, 134.7, 148.8 (C_Ar_), 149.5 (CH=C), 167.8 (C=N), 171.9 (COOH), 197.2 (CH_3_C=O); Anal. Calcd. for C_21_H_19_ClN_2_O_3_S: C 60.79; H 4.62; N 6.75%; Found: C 60.80; H 4.60; N 6.95%.

### 4.12. General Procedures for the Preparation of Compounds ***31**, **32***

**A**: Compound **5** or **6** (5 mmol) was dissolved in toluene (20 mL), and hydrazine monohydrate (0.02 mol, 1 g, 0.97 mL) was added dropwise. The reaction mixture was heated at reflux for 12 h. Then, it was cooled down, formed crystals were filtered off, washed with water, and dried.

**B**: Compound **29** or **30** (5 mmol) was dissolved in 1,4-dioxane (20 mL) and hydrazine monohydrate (0.03 mol, 1.5 g, 1.45 mL) was added dropwise. The reaction mixture was heated at reflux for 24 h. Then, it was cooled down, formed crystals were filtered off, washed with 1,4-dioxane, and dried.

Melting point, ^1^H and ^13^C NMR spectra data of compounds **31**, **32** synthesized according to method **A** comply with those synthesized by method **B**.

*3-{[4-(1-Hydrazineylideneethyl)phenyl](4-phenylthiazol-2-yl)amino}propanehydrazide* (**31**): off-white solid, yield 1.03 g (52 (**A**), 56 (**B**)%); m.p. 106–107 °C (methanol); IR (KBr) (ν_max_, cm**^−^**^1^): 1511 (C=N), 1626 (C=O), 3040 (NH), 3241, 3304 (2NH_2_); ^1^H NMR (400 MHz, DMSO-d_6_) δ (ppm): 2.05 (s, 3H, CH_3_), 2.53–2.56 (m, 2H, CH_2_), 4.19–4.35 (m, 4H, CH_2_, NH_2_), 6.47 (s, 2H, NH_2_), 7.15 (s, 1H, CH), 7.28–7.31 (m, 1H, H_Ar_), 7.39–7.40 (m, 4H, H_Ar_), 7.71 (d, 2H, *J* = 8.2 Hz, H_Ar_), 7.88 (d, 2H, *J* = 7.7 Hz, H_Ar_), 9.09 (s, 1H, CONH); ^13^C NMR (101 MHz, DMSO-d_6_) δ (ppm): 11.8 (CH_3_), 32.4 (CH_2_), 49.7 (NCH_2_), 103.2 (CH=C), 126.2, 126.6, 126.7, 128.0, 129.0, 135.1, 139.1, 141.6 (C_Ar_), 143.8 (CH=C), 150.8 (C=N), 169.1 (C=NNH_2_), 169.9 (C=O); Anal. Calcd. for C_20_H_22_N_6_OS: C 60.89; H 5.62; N 21.30%; Found: C 60.66; H 5.32; N 21.16%.

*3-{[4-(4-Chlorophenyl)thiazol-2-yl][4-(1-hydrazineylideneethyl)phenyl]amino}propanehydrazide* (**32**): white solid, yield 1.2 g (56 (**A**), 59 (**B**)%); m.p. 128–129 °C (propan-2-ol); IR (KBr) (ν_max_, cm**^−^**^1^): 1509 (C=N), 1702 (C=O), 3043 (NH), 3114, 3233 (2NH_2_); ^1^H NMR (400 MHz, DMSO-d_6_) δ (ppm): 2.05 (s, 3H, CH_3_), 2.52 (s, 2H, CH_2_), 4.08–4.25 (m, 4H, NH, CH_2_), 6.48 (s, 2H, NH_2_), 7.22 (s, 1H, CH), 7.39 (d, 2H, *J* = 8.3 Hz, H_Ar_), 7.46 (d, 2H, *J* = 8.3 Hz, H_Ar_), 7.71 (d, 2H, *J* = 8.3 Hz, H_Ar_), 7.90 (d, 2H, *J* = 8.3 Hz, H_Ar_); 9.09 (s, 1H, NH); ^13^C NMR (101 MHz, DMSO-d_6_) δ (ppm): 11.8 (CH_3_), 32.4 (CH_2_), 49.7 (NCH_2_), 104.0 (CH=C), 126.6, 126.8, 127.9, 129.0, 132.4, 134.0, 139.2, 141.5 (C_Ar_), 143.6 (CH=C), 149.6 (C=NNH_2_), 169.3 (C=N), 169.8 (C=O); Anal. Calcd. for C_20_H_21_ClN_6_OS: C 56.00; H 4.93; N 19.59%; Found: C 55.89; H 4.67; N 19.36%.

### 4.13. Preparation of the Test Compounds and Screening Library

The test compounds **2**–**32** were dissolved in hybridoma-grade dimethyl sulfoxide (Millipore, Sigma, Burlington, MA, USA) to prepare stock solutions at concentrations of 10–25 mg/mL. Cisplatin and doxorubicin hydrochloride were dissolved in DMSO and (MedChemExpress, Monmouth Junction, NJ, USA). The dissolved compounds were then manually dispensed into deep 96-well plates, sealed, and stored at −80 °C until the day of the experiment. For the in vitro antiproliferative activity screening, the compounds were thawed at room temperature, protected from light, and the aliquots were diluted in complete cell culture media to achieve a final concentration of 100 µM and used for the in vitro assays.

### 4.14. Cell Lines and Culture Conditions

The A549 non-small cell human lung carcinoma cells (ATCC CCL-185), H69 (HTB-19), H69AR (CRL-11351) were obtained from the American Type Culture Collection (Rockville, MD, USA). HEK293 cells were kindly provided by Dr. Iliev lab at Jill Roberts Institute for Inflammatory Bowel Disease, Weill Cornel Medicine of Cornel University (New York, NY, USA). All cells were cultivated in Dulbecco’s Modified Eagle Medium/Nutrient Mixture F-12 (DMEM/F-12) (Gibco, Waltham, MA, USA), 10% fetal bovine serum (FBS) (Gibco, Waltham, MA, USA), 100 U/mL penicillin, and 100 μg/mL streptomycin (P/S) (Gibco, Waltham, MA, USA). Culturing conditions were maintained at 37 °C with a humidified atmosphere containing 5% CO_2_. The culture medium was refreshed every 2–3 days, and cells were passaged upon reaching 70–80% confluence.

### 4.15. MTT Based Cell Viability Assay

The in vitro inhibitory effects of the compounds were assessed using the MTT assay [40,41,42,43,44]. Briefly, cells were plated in 96-well plates at a density of 1 × 10^4^ cells per well. After allowing the cells to adhere overnight at 37 °C in 5% CO_2_, they were treated with compounds at concentration of 100 µM in triplicate. Following a 20 h incubation, MTT reagent was added, and the cells were further incubated for 4 h. The resulting formazan was solubilized in anhydrous DMSO, and absorbance was measured at 570 nm using a microplate reader. Cell viability was calculated using the following formula: ([AE − AB]/[AC − AB]) × 100%, where AE, AC, and AB represent the absorbance values of experimental samples, untreated controls, and blank wells, respectively. Data analysis was performed using GraphPad Prism or QuickCalcs (2.0 version).

### 4.16. Generation of A549 3D Spheroids

A549 tumor spheroids were generated using an agarose-based technique with modifications [45,46,47]. A549 cells were plated at a density of 2.5 × 10^5^ cells/well in 96-well plates pre-coated with 50 µL of 1% agarose in Dulbecco’s Phosphate-Buffered Saline (DPBS). The plates were incubated for 48 h to allow spheroid formation. After 48 h, the spheroids were treated with 100 µM of compounds dissolved in DMEM/F12 supplemented with 10% FBS and 0.25% DMSO for 24 h.

### 4.17. Acridine Orange/Propidium Iodide (AO/PI) Staining of A549 Spheroids

After treatment with compounds, spheroids were incubated with 5 µg/mL acridine orange and 5 µg/mL propidium iodide for 30 min at 37 °C in a humidified incubator. Following incubation, the spheroids were washed twice with DPBS to remove excess stain. The stained spheroids were then visualized using the EVOS cell imaging system (Thermo Fisher Scientific, Waltham, MA, USA). The fluorescent images were captured, and the viability of the spheroids was assessed based on the differential staining patterns, with live cells showing green fluorescence (acridine orange) and dead cells displaying red fluorescence (propidium iodide).

### 4.18. Compound-Induced Cytotoxicity Evaluation in A549 Spheroids Using LDH Assay

A549 tumor spheroids were generated in 96-well plates pre-coated with 50 µL of 1% agarose in Dulbecco’s Phosphate-Buffered Saline (DPBS), as described previously. After 48 h of spheroid formation, the spheroids were treated with compounds dissolved in DMEM/F12 supplemented with 10% FBS and 0.25% DMSO. After a 24 h incubation, spheroid-conditioned media were collected by centrifuging the plates at 1000× *g* for 2 min to remove the spheroids. Then, 50 µL of the supernatant was transferred to a new 96-well plate and mixed with LDH assay reagent (Pierce LDH Cytotoxicity Assay Kit, Thermo Fisher Scientific, Waltham, MA, USA) according to the manufacturer’s instructions. A commercial maximum LDH control was used to normalize the results. The percentage of LDH release was calculated based on the absorbance readings, using the formula: (sample absorbance − blank absorbance)/(maximum control absorbance − blank absorbance) × 100%.

### 4.19. IC_50_ Determination

The IC_50_ values, defined as the concentration of compound required to reduce cell viability by 50%, were determined using a dose–response curve. The data were fitted to a nonlinear regression model (GraphPad Prism version 9.0, GraphPad Software, San Diego, CA, USA) to calculate the IC_50_ values for each compound tested in triplicate.

### 4.20. The Protein Target Preparation

Upon comprehensive literature review, biological targets overexpressed in lung cancer cells were found, such as cyclooxygenase-2 (COX-2), kinases (MAPK1, ERK2, MEK1, TPK, CK4), mesenchymal–epithelial transition factor (c-MET), membrane receptors (FGFR, VEGFR-2, NR3A1), among others. Complementarily, we carried out a search for potential targets on the “SwissTargetPrediction” online platform using the structure of compound **22** [48,49,50,51,52,53,54,55,56,57,58,59,60,61,62,63,64]. The crystal structure of 18 selected proteins (Table S1) were retrieved from the Protein Data Bank [48].

### 4.21. Ligand Preparation

The 3D structure of the candidate compounds **21**, **22**, **25** and **26** were built using GaussView 5.0 and geometrically optimized using Avogadro [53]. These structures were visually checked to correct some structural errors. Three-dimensional structure of ligands was extracted from crystal 4ISI and 3RHK, whereas structure of inhibitor drugs (sirtinol and erlotinib) was taken PubChem database.

### 4.22. Docking of Ligand–Protein Interaction

The compounds were docked into proteins to identify their potential binding site. Both ligand and protein were prepared using AutoDock Tools version 1.5.7 according to the AutoDock Vina High Throughput screening standard method. Gasteiger partial charges were assigned to the atoms of the ligand. The AutoTors option was used to define the rotatable bonds in the ligand. The visual inspection of the results was performed using the Molecular Graphics Laboratory Tools package (2.1 version). We selected a grid size enough to cover each receptor. Finally, graphical analysis was performed using VMD [55] and Discovery Studio [55].

### 4.23. Statistical Analysis

The data are expressed as mean ± SD from three independent experiments, unless otherwise stated. Statistical significance was determined using a one-way ANOVA test in GraphPad Prism software. A *p* < 0.05 was considered statistically significant.

## 5. Conclusions

In this study, a series of novel functionalized thiazole derivatives were synthesized from 3-[1-(4-acetylphenyl)thioureido]propanoic acid and α-halocarbonyl compounds. The transformations of the synthesized aminothiazoles were investigated, revealing that the methine group of the functionalized thiazole ring is sufficiently reactive to undergo condensation reactions with aromatic aldehydes, leading to the formation of functionalized bis(thiazol-5-yl)phenylmethanes. By leveraging the functional properties of the acetyl and carboxyl groups, additional derivatives containing chalcone, ester, hydroxyimine, hydrazine, and hydrazone moieties were synthesized.

The synthesized compounds exhibited structure-dependent antiproliferative activity in A549 human lung adenocarcinoma cell models. Furthermore, the most promising derivatives—**21**, **22**, **25**, and **26**—demonstrated activity in both drug-sensitive H69 and multidrug-resistant H69AR small-cell lung cancer models, suggesting that the 3-[1-(4-acetylphenyl)thioureido]propanoic acid scaffold may be further explored for targeting drug-resistant cancer cells. Notably, the most active compounds also exhibited efficacy in a 3D A549 spheroid model.

Further studies are required to better elucidate the in vivo safety, pharmacological properties, and molecular targets of the 3-[1-(4-acetylphenyl)thioureido]propanoic acid scaffold.

## Figures and Tables

**Table 1:** The half-maximal inhibitory concentration (IC_50_) values of the most promising selected compounds in A549 human lung adenocarcinoma cells.

Compound	R	IC_50_ (µM)
**21**	H	5.42
**22**	Cl	2.47
**25**	H	8.05
**26**	Cl	25.40
CP	-	11.71
DOX	-	3.02

**Table 2:** Predicted binding free energy values (∆G_bin_ kcal/mol) of synthesized cytotoxic hybrids with selected proteins overexpressed in cancer cells.

Compounds	Target Proteins									
	COX-2	FGFR-2	EGFR	HER2	c-MET	ERK2	MEK1	TPK	PD2	SIRT1
**21**	−8.2	−9.0	−9.4	−8.9	−8.6	−8.1	−8.1	−7.9	−9.7	−9.5
**22**	−8.5	−8.9	−9.5	−8.6	−8.5	−7.6	−8.2	−8.2	−9.1	−9.7
**25**	−8.6	−9.5	−9.3	−8.7	−8.5	−8.0	−8.6	−8.4	−9.5	−10.0
**26**	−8.9	−8.3	−9.2	−8.9	−8.5	−8.5	−8.0	−8.4	−9.3	−10.2
**P aver.**	**−** **8.55**	**−** **8.93**	**−** **9.35**	**−** **8.78**	**−** **8.53**	**−** **8.05**	**−** **8.23**	**−** **8.23**	**−9.40**	**−** **9.85**

Proteins with their respective (PDB) entries: COX-2: Cyclooxygenase 2 (3LN2), FGFR-2: Fibroblast growth factor receptor 2 (1GJO); EGFR: Epidermal growth factor receptor (5GTY); HER2: Epidermal growth factor receptor 2 (7JXH); c-MET: Mesenchymal–epithelial transition factor (3RHK); ERK2: Extra-cellular signal-regulated kinase 2 (2OJG); MEK1: MAPK/ERK kinase (4AN3); TPK: Tyrosine-protein kinase (4EHZ); PD2: Prostaglandin D2 receptor (6D26) SIRT1: NAD-dependent sirtuin-1 deacetylase (4ISI). P avge.: Protein average. Mean of the ∆G_bin_ values for the interactions of each protein with all the hybrids; the two proteins with the highest global compounds affinity are highlighted on red color.

**Table 3:** Binding site contacts of compound **22**, ligands, and drugs into SIRT1 and EGFR.

Compound	ΔG*_bin_* (kcal/mol)	H Bonds and Hydrophobic Contacts in the Binding Site
		SIRT1
**22**	−9.7	Gly261, Ala262, Ile270, Phe273, Arg274, Phe297, Ile316, Gln345, Asn346, Ile347, His363, Ile411, **Val412**, Phe413, **Phe414**, Ser441, **Ser442**, **Leu443**, Lys444, Val445
**Ligand 1 ^[a]^**	−11.2	Ala262, Ser265, Ile270, Pro271, Phe273, Ile279, Phe297, Ile316, Gln345, Asn346, **Ile347**, **Asp348**, His363, Ile411, Val412, Phe413
**Sirtinol ^[b]^**	−11.7	Gly261, Ala262, Phe273, Arg274, Tyr280, Gln294, Phe297, Gln345, Asn346, Ile347, His363, Ile411, Val412, Phe413, Phe414, Val445
		**EGFR**
**22**	−9.5	Leu718, Gly721, Val726, Ala743, Lys745, Met766, Leu777, Leu788, Met790, Gln791, Leu792, **Met793**, Gly796, Cys797, Arg841, **Asn842**, Leu844, Thr854, Asp855, Leu858
**Ligand 2 ^[a]^**	−6.2	Leu718, Gly719, Ser720, Val726, Ala743, Ile744, Lys745, Cys775, Arg776, Leu777, Leu788, Ile789, Met790, Gln791, Leu792, **Met793**, Gly796, Cys797, Asp800, Arg841, Leu844, Thr854, Asp855, Phe856, Leu858
**Erlotinib ^[b]^**	−8.6	Leu718, Gly721, Val726, Ala743, Lys745, Met766, Cys775, Arg776, Leu777, Leu788, Met790, Leu792, Met793, Gly796, **Arg841**, Asn842, Leu844, Thr854, Asp855, Phe856, Leu858

[a] Ligands 1 and 2 correspond to (6*S*)-2-chloro-5,6,7,8,9,10-hexahydrocyclohepta[b]indole-6-carboxamide and 1-[(3*R*)-3-[4-azanyl-3-[3-chloranyl-4-[(6-methylpyridin-2-yl)methoxy]phenyl]pyrazolo [3,4-d]pyrimidin-1-yl]piperidin-1-yl]prop-2-en-1-one, respectively. Three-dimensional structures of ligands 1 and 2 were extracted from the Protein Data Bank using PDB IDs 4I5I, and 5GTY, respectively. [b] Drug compounds that act as inhibitors of biological targets. Words colored in blue, green, and red correspond to amino acids shared by **22** and ligands; **22** and drugs; and **22**, ligands, and drugs, respectively. Bolded names correspond to amino acids involved in H bonds.

## Data Availability

The data presented in this study are available on request from the corresponding author due to new results being obtained in this research field.

## Supplementary Materials

The following supporting information can be downloaded at https://www.mdpi.com/article/10.3390/ph18050733/s1, Figure S1. ^1^H NMR spectrum of compound **3**. Figure S2. ^13^C NMR spectrum of compound **3**. Figure S3. ^1^H NMR spectrum of compound **4**. Figure S4. ^13^C NMR spectrum of compound **4**. Figure S5. ^1^H NMR spectrum of compound **5**. Figure S6. ^13^C NMR spectrum of compound **5**. Figure S7. ^1^H NMR spectrum of compound **7**. Figure S8.^13^C NMR spectrum of compound **7**. Figure S9. ^1^H NMR spectrum of compound **7**. Figure S10. ^13^C NMR spectrum of compound **7**. Figure S11. ^1^H NMR spectrum of compound **8**. Figure S12. ^13^C NMR spectrum of compound **8**. Figure S13. ^1^H NMR spectrum of compound **9**. Figure S14. ^13^C NMR spectrum of compound **9**. Figure S15. ^1^H NMR spectrum of compound **10**. Figure S16. ^13^C NMR spectrum of compound **10**. Figure S17. ^1^H NMR spectrum of compound **11**. Figure S18. ^13^C NMR spectrum of compound **11**. Figure S19. ^1^H NMR spectrum of compound **12**. Figure S20. ^13^C NMR spectrum of compound **12**. Figure S21. ^1^H NMR spectrum of compound **13**. Figure S22. ^13^C NMR spectrum of compound **13**. Figure S23. ^1^H NMR spectrum of compound **14**. Figure S24. ^13^C NMR spectrum of compound **14**. Figure S25. ^1^H NMR spectrum of compound **15**. Figure S26. ^13^C NMR spectrum of compound **15**. Figure S27. ^1^H NMR spectrum of compound **16**. Figure S28. ^13^C NMR spectrum of compound **16**. Figure S29. ^1^H NMR spectrum of compound **17**. Figure S30. ^13^C NMR spectrum of compound **17**. Figure S31. ^1^H NMR spectrum of compound **18**. Figure S32. ^13^C NMR spectrum of compound **18**. Figure S33. ^1^H NMR spectrum of compound **19**. Figure S34. ^13^C NMR spectrum of compound **19**. Figure S35. ^1^H NMR spectrum of compound **20**. Figure S36. ^13^C NMR spectrum of compound **20**. Figure S37.^1^H NMR spectrum of compound **21**. Figure S38. ^13^C NMR spectrum of compound **21**. Figure S39. ^1^H NMR spectrum of compound **22**. Figure S40. ^13^C NMR spectrum of compound **22**. Figure S41. ^1^H NMR spectrum of compound **23**. Figure S42. ^13^C NMR spectrum of compound **23**. Figure S43. ^1^H NMR spectrum of compound **24**. Figure S44. ^13^C NMR spectrum of compound **24**. Figure S45. ^1^H NMR spectrum of compound **25**. Figure S46. ^13^C NMR spectrum of compound **25**. Figure S47. ^1^H NMR spectrum of compound **26**. Figure S48. ^13^C NMR spectrum of compound **26**. Figure S49. ^1^H NMR spectrum of compound **27**. Figure S50. ^13^C NMR spectrum of compound **27**. Figure S51. ^1^H NMR spectrum of compound **28**. Figure S52. ^13^C NMR spectrum of compound **28**. Figure S53. ^1^H NMR spectrum of compound **29**. Figure S54. ^13^C NMR spectrum of compound **29**. Figure S55. ^1^H NMR spectrum of compound **30**. Figure S56. ^13^C NMR spectrum of compound **30**. Figure S57. ^1^H NMR spectrum of compound **31**. Figure S58. ^13^C NMR spectrum of compound **31**. Figure S59. ^1^H NMR spectrum of compound **32**. Figure S60. ^13^C NMR spectrum of compound **32**. Figure S61. The dose–response kinetics of selected compounds **21**, **22** and **25**, **26**, the most promising candidates, in A549 human lung carcinoma cells. The A549 cells were exposed to increasing concentrations of the compounds for 24 h, and viability was measured using the MTT assay. Data shown are mean ± SD values from three independent experiments for each group. Figure S62. The dose–response kinetics and IC_50_ values of cisplatin and doxorubicin (DOX) in A549 cells. The cells were exposed to cisplatin (CP) and doxorubicin (DOX) for 24 h and the viability was determined using MTT assay. Table S1. Predicted binding free energy values (∆G_bin_ kcal/mol) of synthesized cytotoxic hybrids with selected proteins overexpressed in cancer cells.
