# Supplementary material for: Identification of 3-[(4-Acetylphenyl)(4-Phenylthiazol-2-Yl)Amino]Propanoic Acid Derivatives as Promising Scaffolds for the Development of Novel Anticancer Candidates Targeting SIRT2 and EGFR
Source: Pharmaceuticals (Basel). 2025 May 16;18(5):733. doi: 10.3390/ph18050733 (PMC12115147; doi:10.3390/ph18050733)
Supplement: Supplementary file 1 [file pharmaceuticals-18-00733-s001.zip › pharmaceuticals-3584671-supplementary.pdf]

Supplementary Materials

Identification of 3-[(4-Acetylphenyl)(4-Phenylthiazol-2-Yl)Amino]Propanoic Acid Derivatives as Promising Scaffolds for the Development of Novel Anticancer Candidates Targeting SIRT2 and EGFR

NMR Spectra (compounds 3–32, all*in DMSO-d_6, Figures S1–S60, *compound 4 ^{1}H NMR in Acetone- d_6, Figure S3)

1-(4-Aacetylphenyl)-2-thioxotetrahydropyrimidin-4(1H)-one (**3**)

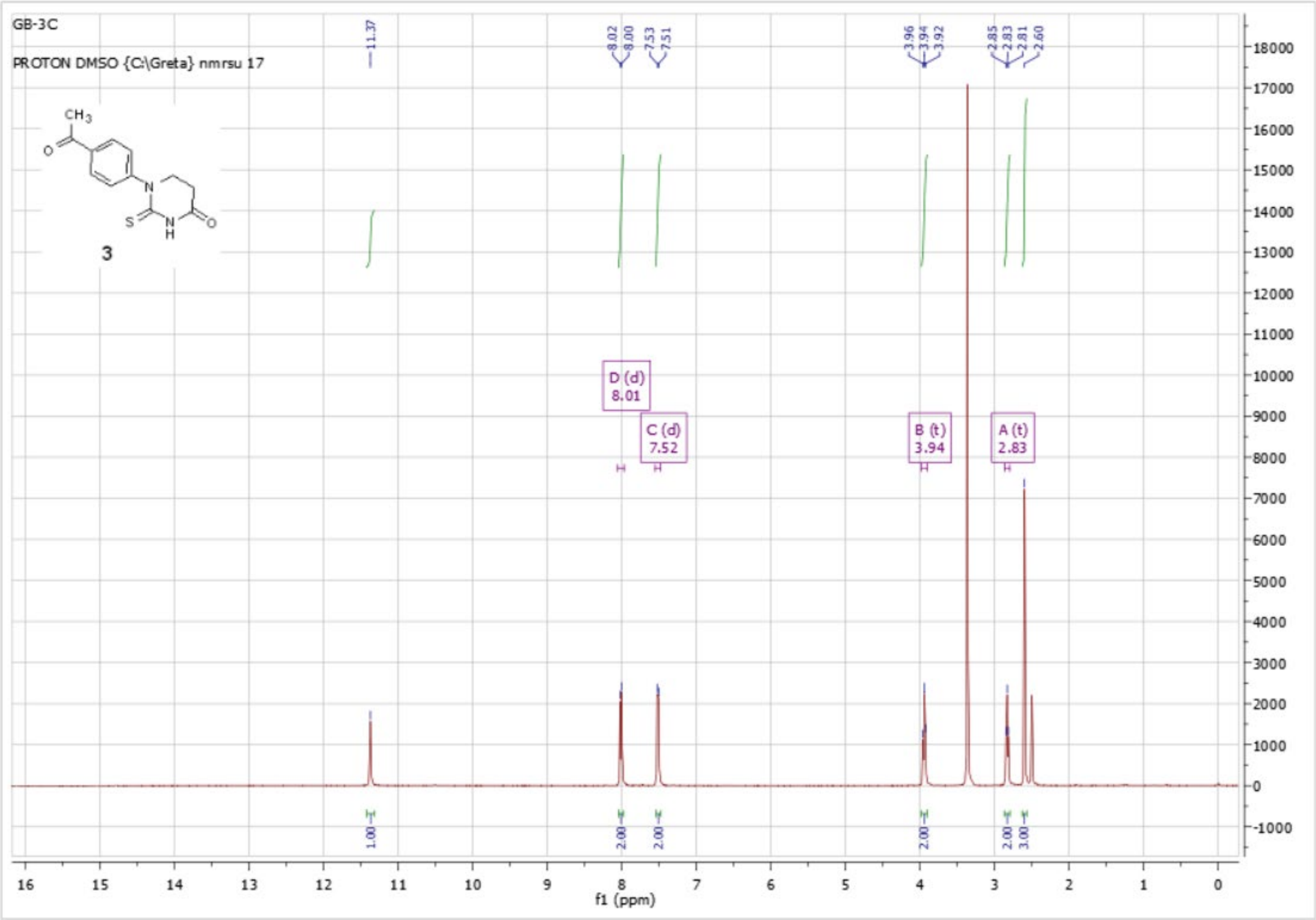

Figure S1. ^{1}H NMR spectrum of compound **3.**

1-(4-Acetylphenyl)-2-thioxotetrahydropyrimidin-4(1H)-one (**3**)

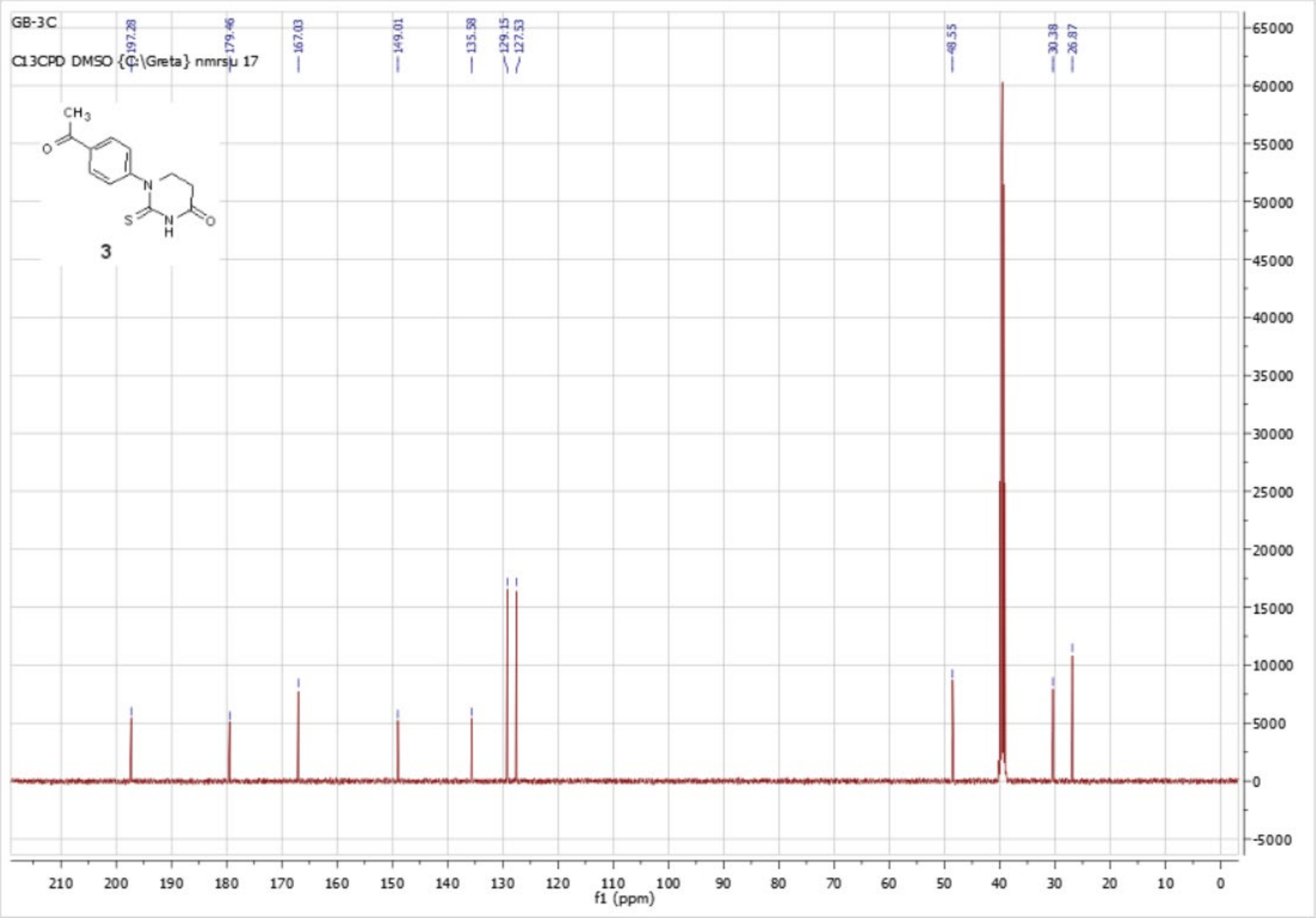

Figure S2. ^{13}C NMR spectrum of compound **3**.

3-(1-(4-Acetylphenyl)thioureido)propanoic acid (**4**)

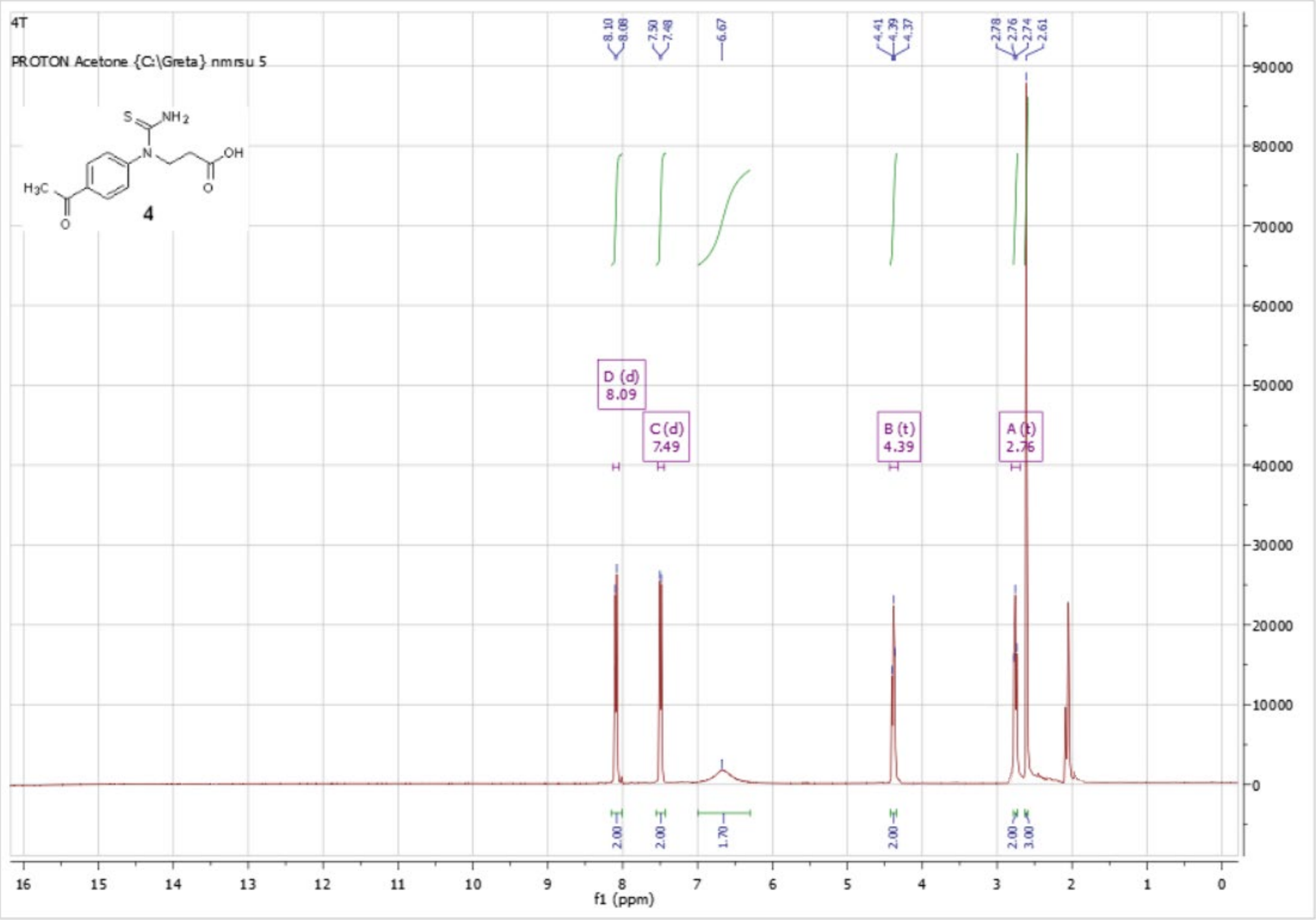

Figure S3. ^{1}H NMR spectrum of compound **4**.

3-(1-(4-Acetylphenyl)thioureido)propanoic acid (**4**)

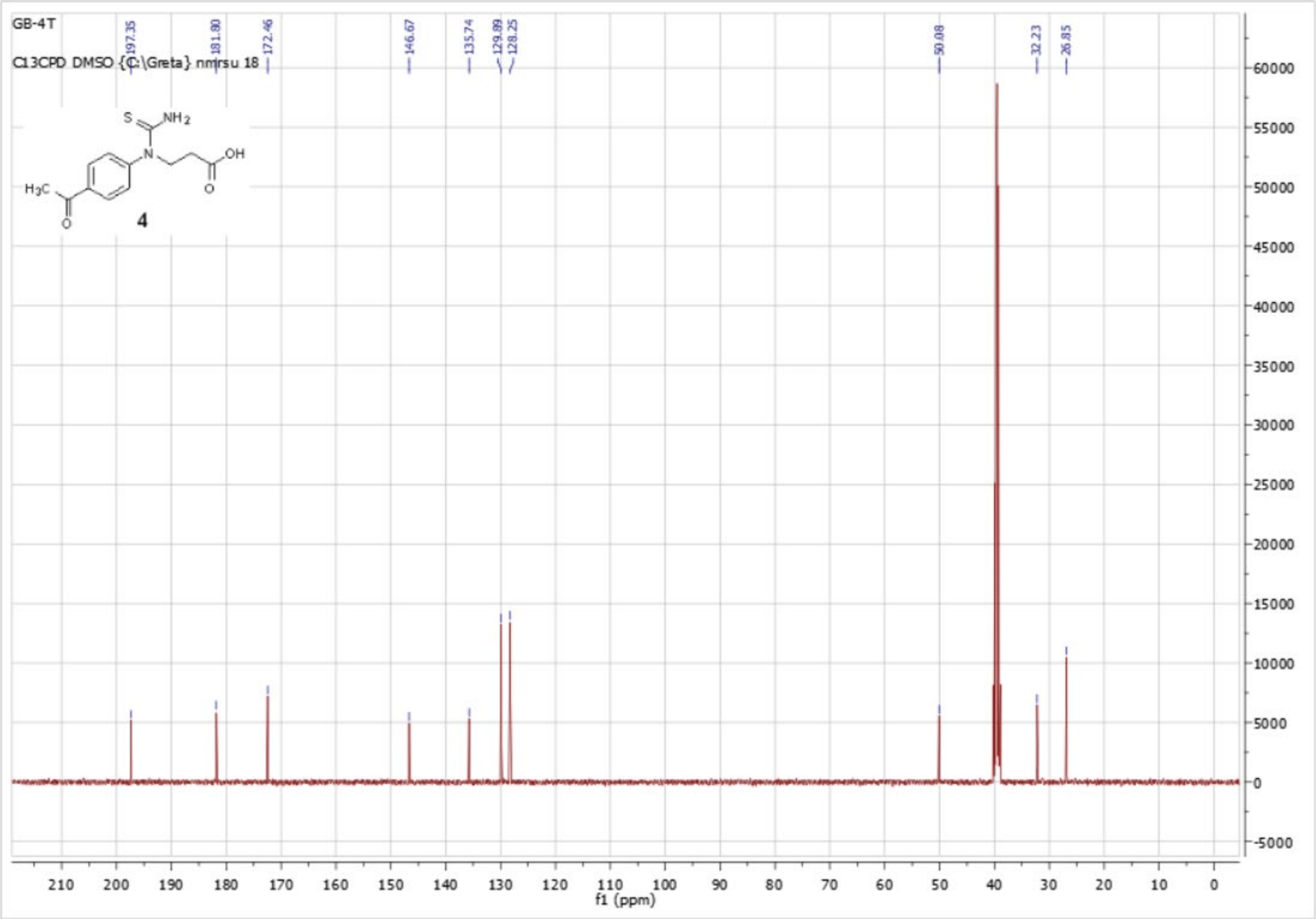

Figure S4. ^{13}C NMR spectrum of compound **4**.

*3-[(4-Acetylphenyl)(4-phenylthiazol-2-yl)amino]propanoic acid (**5**)*

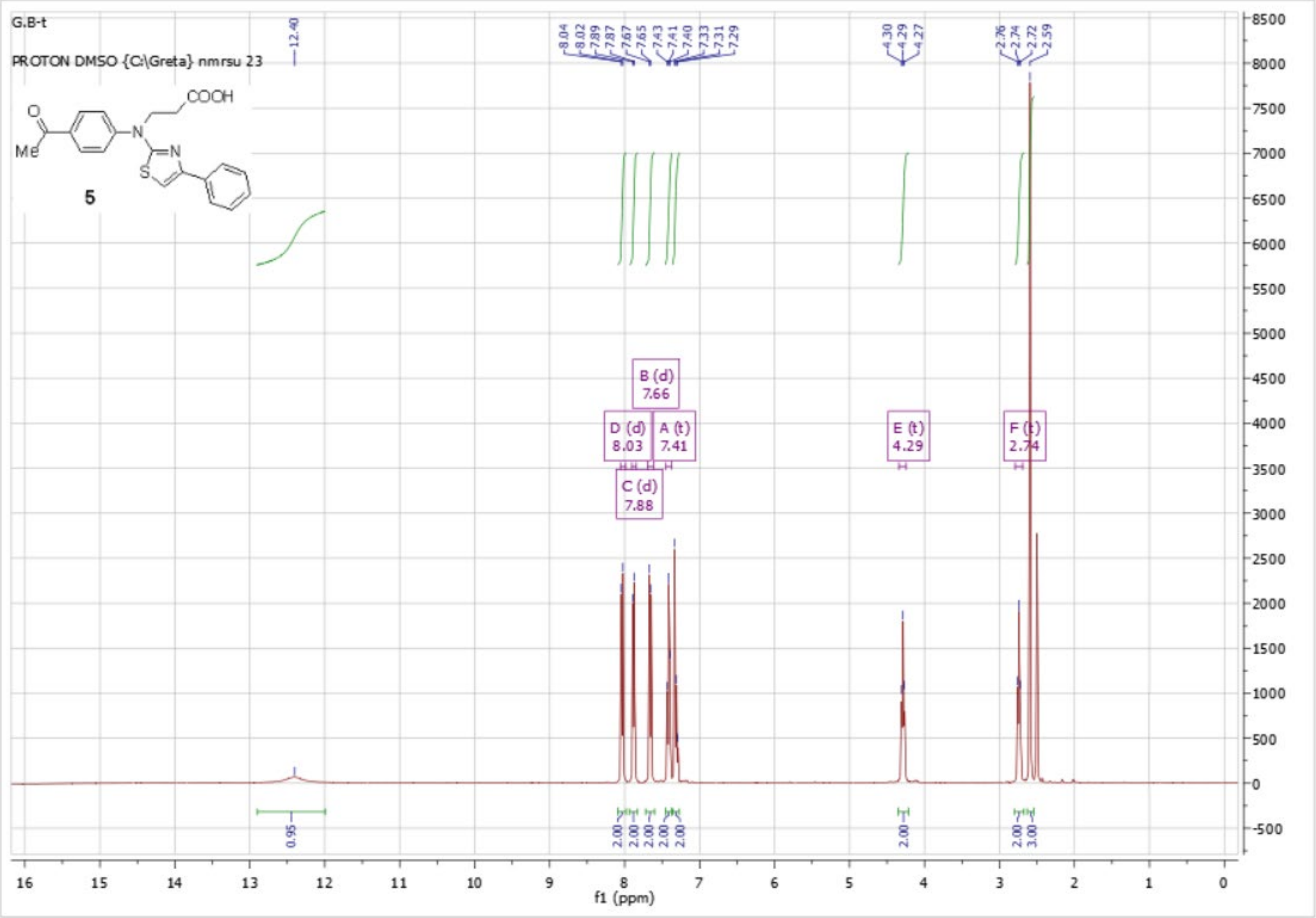

Figure S5. ^{1}H NMR spectrum of compound **5.**

*3-[(4-Acetylphenyl)(4-phenylthiazol-2-yl)amino]propanoic acid (**5**)*

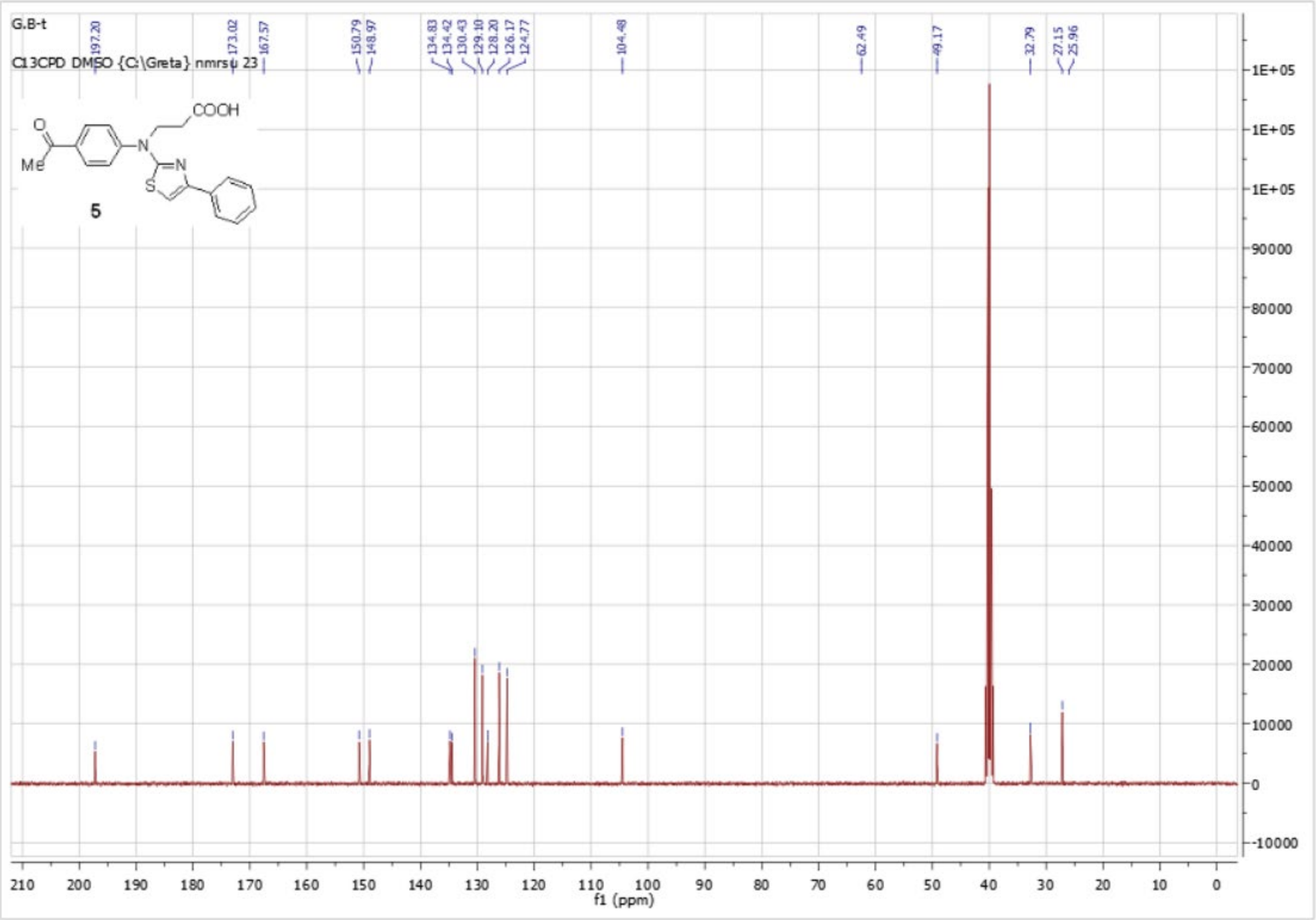

Figure S6. ^{13}C NMR spectrum of compound **5.**

*3-{(4-Acetylphenyl)[4-(4-chlorophenyl)thiazol-2-yl]amino}propanoic acid (**6**)*

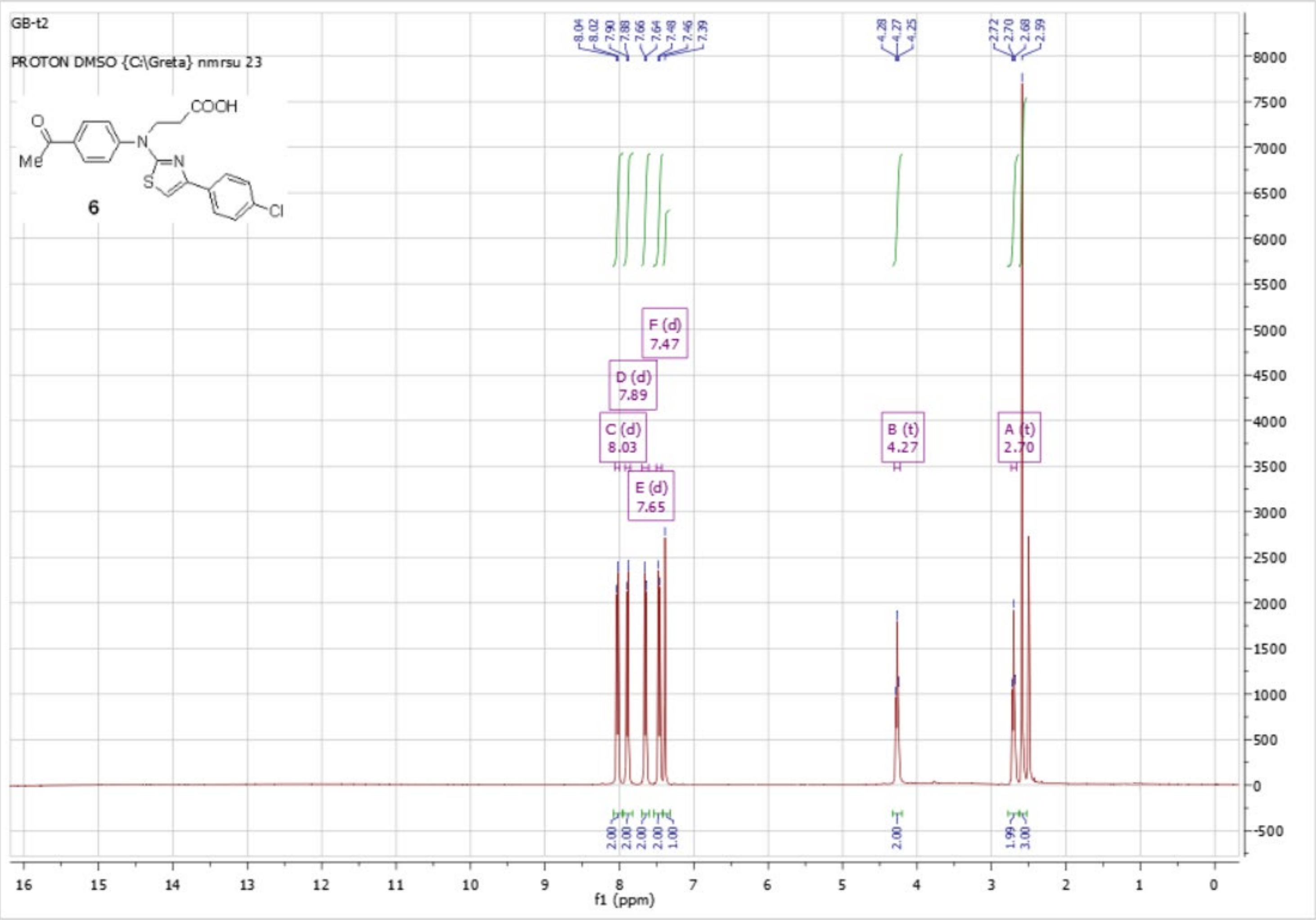

Figure S7. ^{1}H NMR spectrum of compound **6.**

*3-{(4-Acetylphenyl)[4-(4-chlorophenyl)thiazol-2-yl]amino}propanoic acid (**6**)*

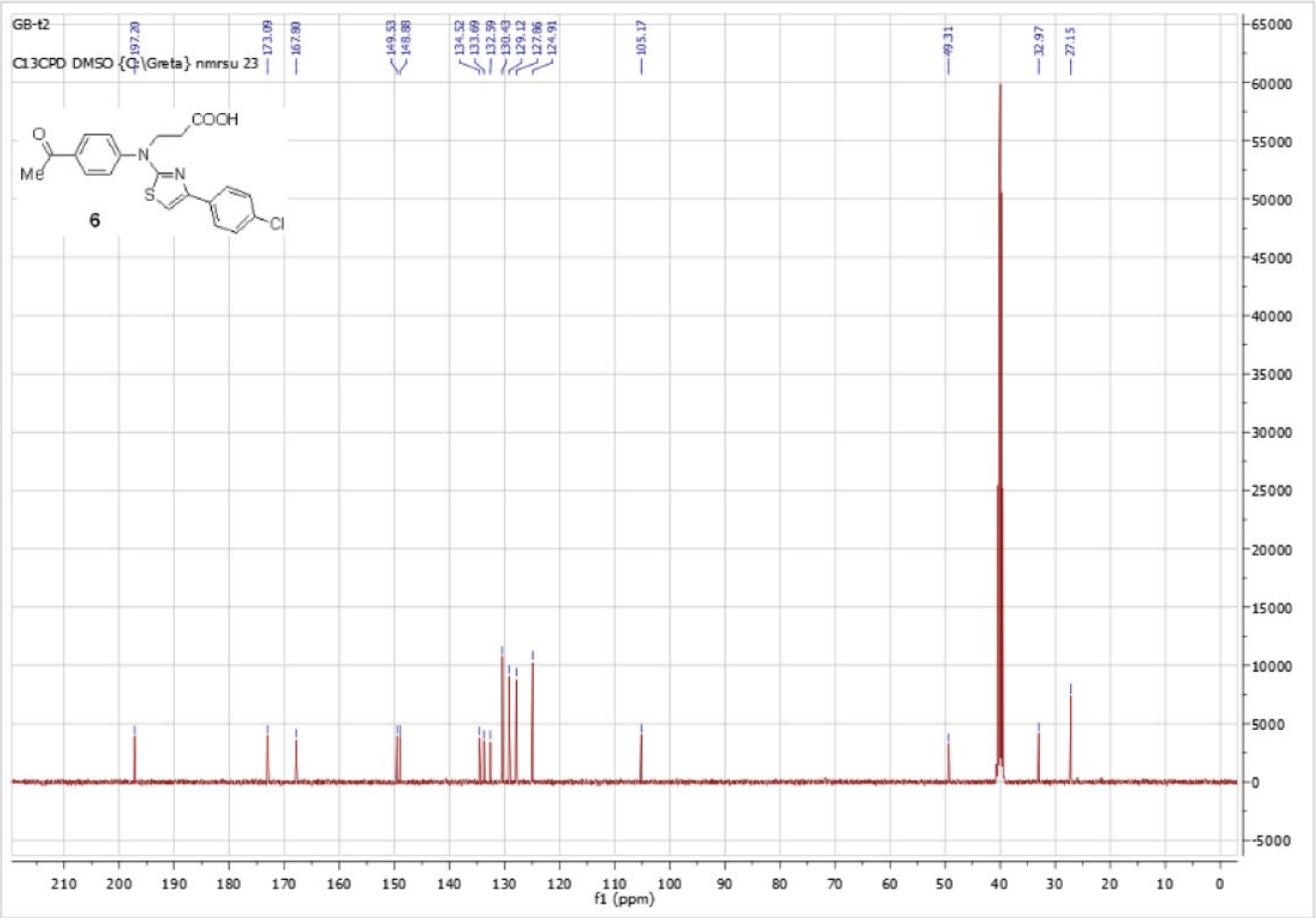

Figure S8.^{13}C NMR spectrum of compound **6.**

*3-[(4-Acetylphenyl)[4-(4-nitrophenyl)thiazol-2-yl]amino}propanoic acid (**7**)*

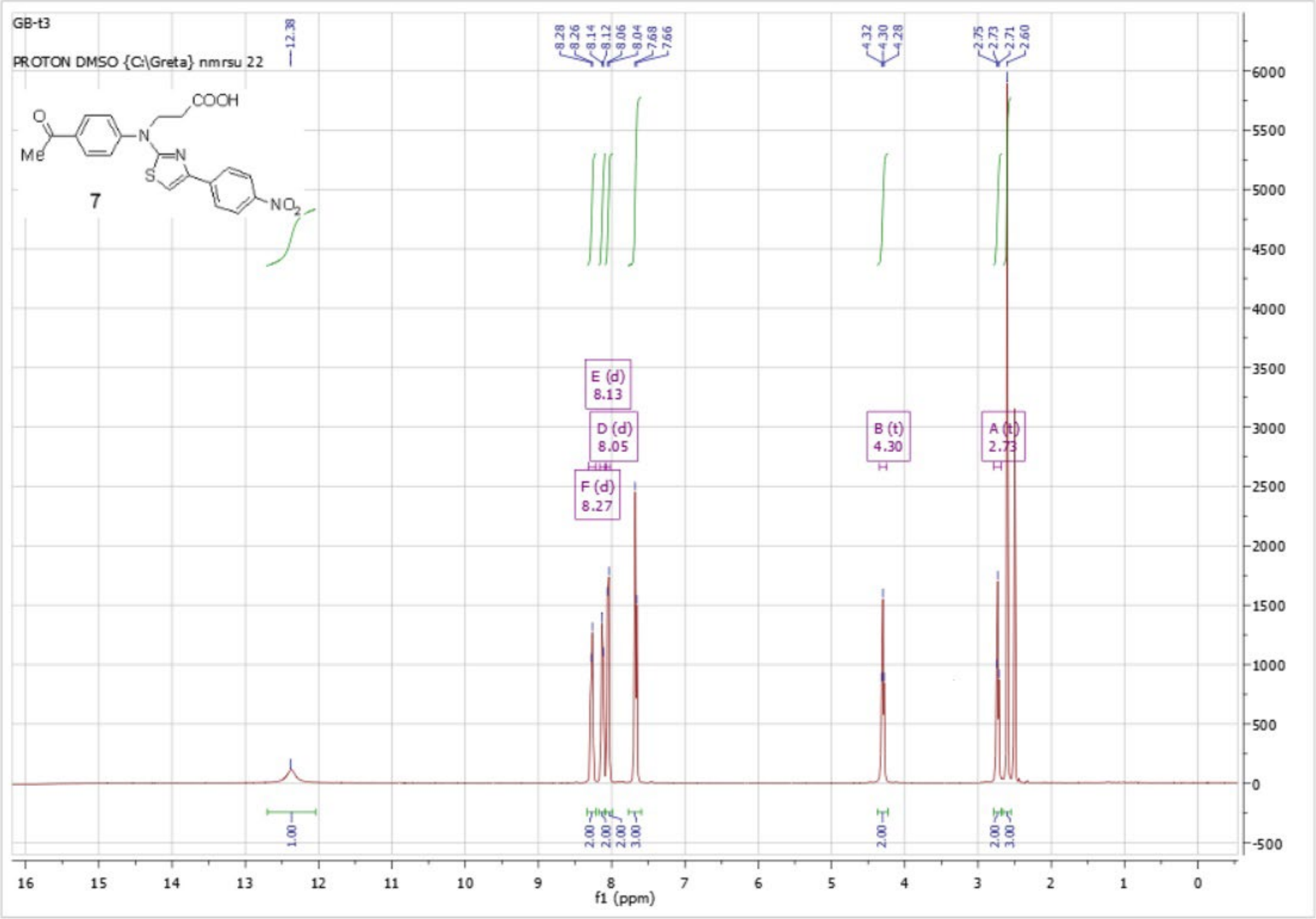

Figure S9. ^{1}H NMR spectrum of compound **7**.

*3-[(4-Acetylphenyl)[4-(4-nitrophenyl)thiazol-2-yl]amino}propanoic acid (**7**)*

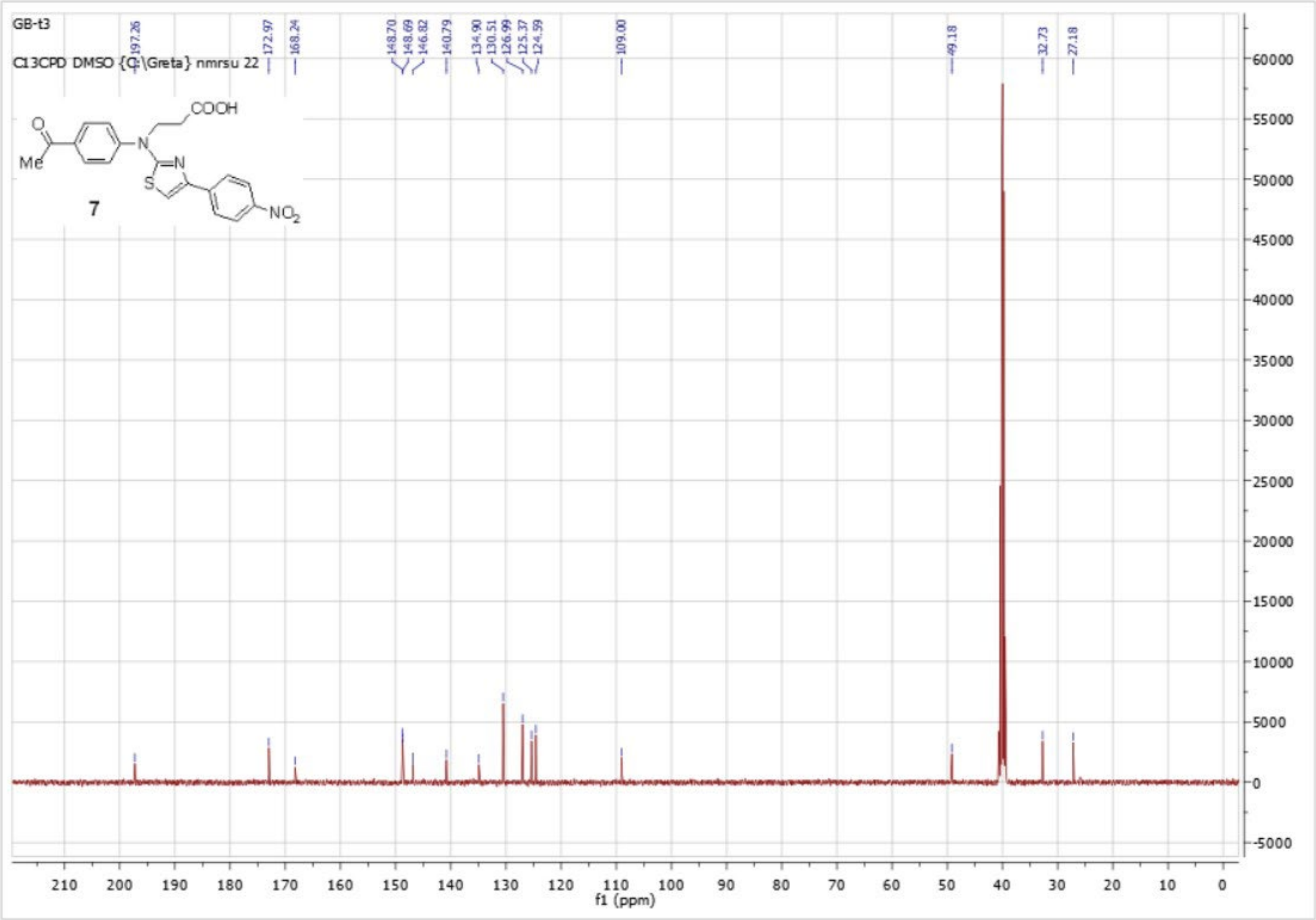

Figure S10. ^{13}C NMR spectrum of compound **7**.

3-{(4-Acetylphenyl)[4-(4-cyanophenyl)thiazol-2-yl]amino}propanoic acid (8)

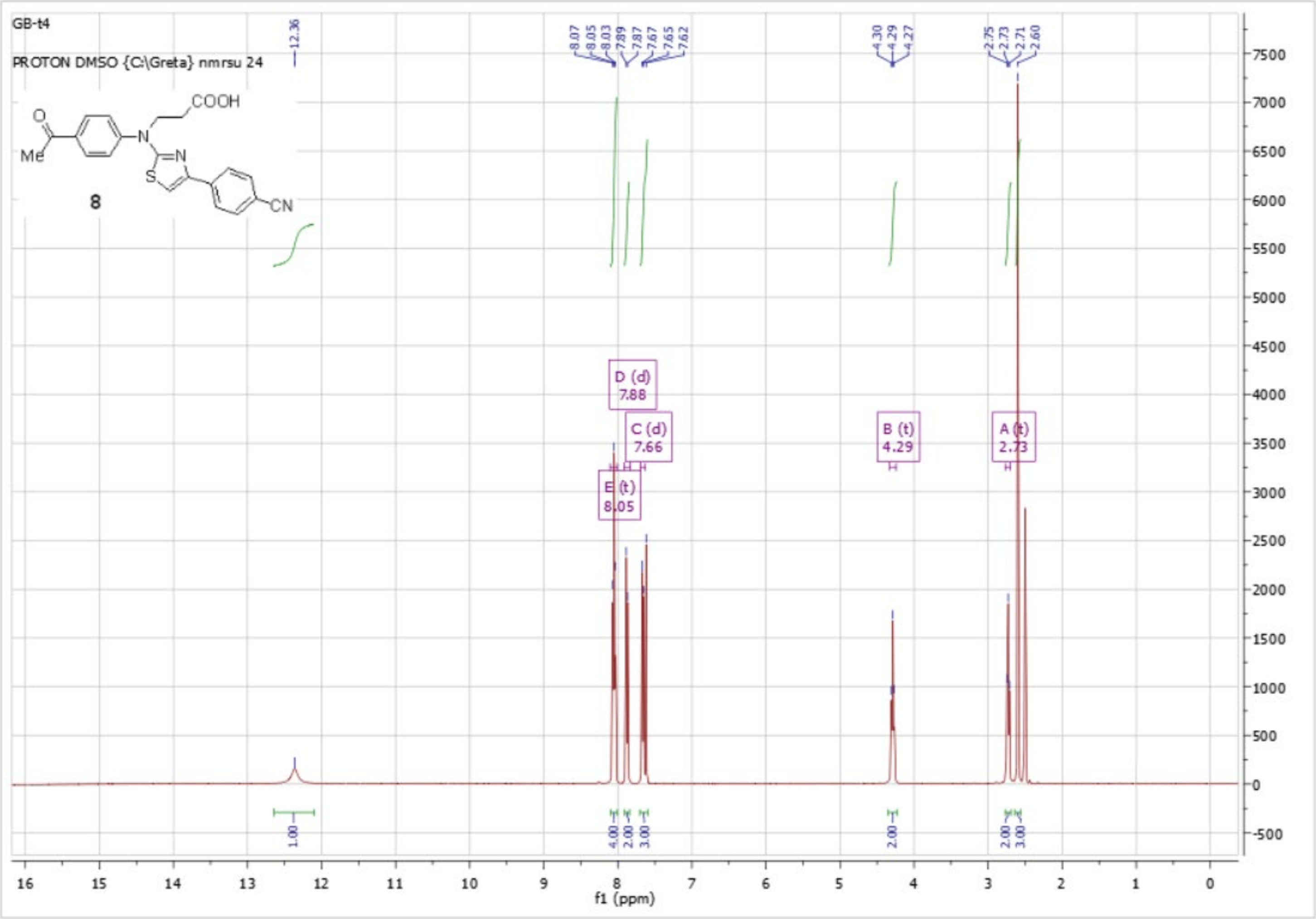

Figure S11. ^{1}H NMR spectrum of compound **8**.

*3-{(4-Acetylphenyl)[4-(4-cyanophenyl)thiazol-2-yl]amino}propanoic acid (**8**)*

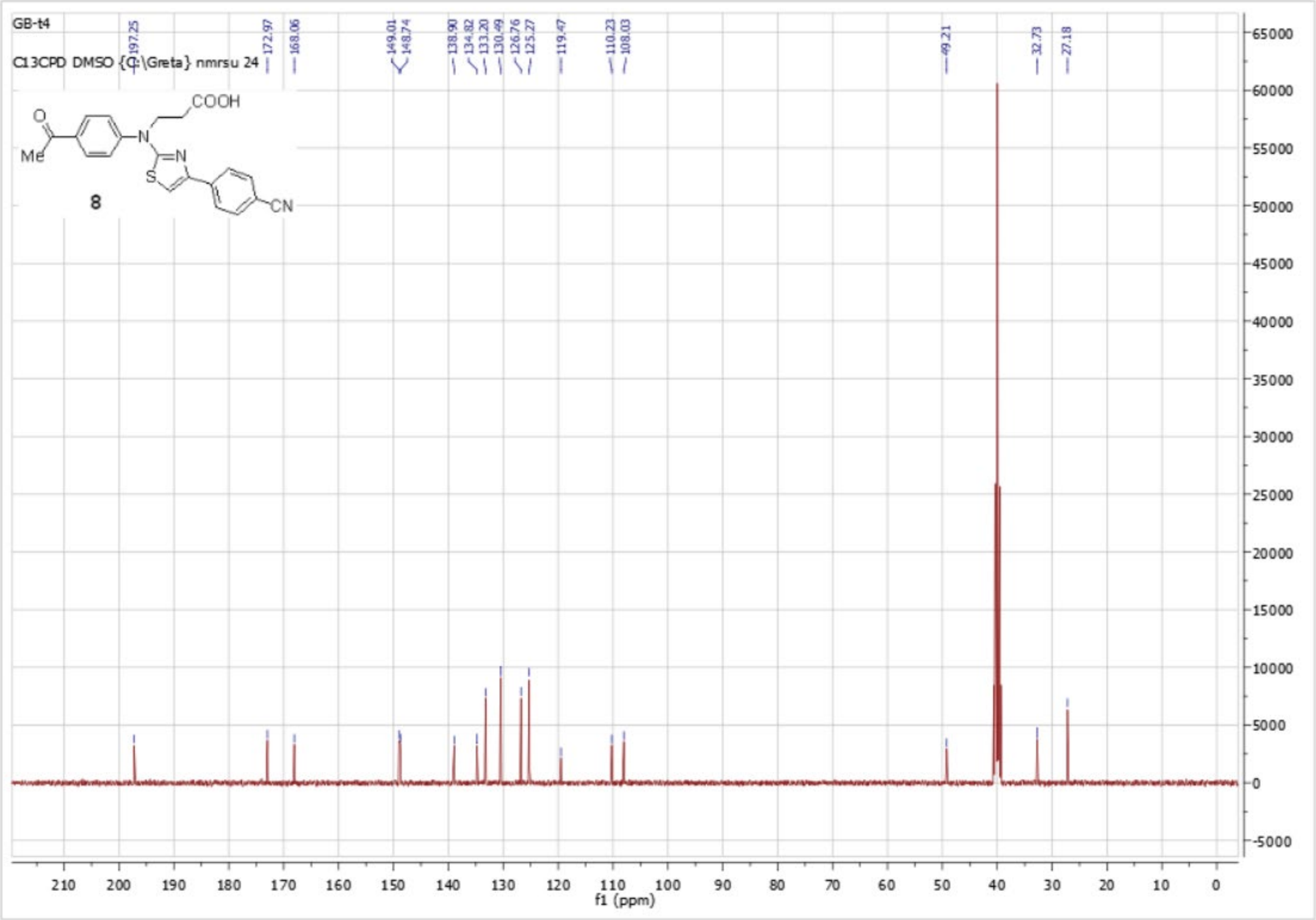

Figure S12. ^{13}C NMR spectrum of compound **8**.

*3-{(4-Acetylphenyl)[4-(4-fluorophenyl)thiazol-2-yl]amino}propanoic acid (**9**)*

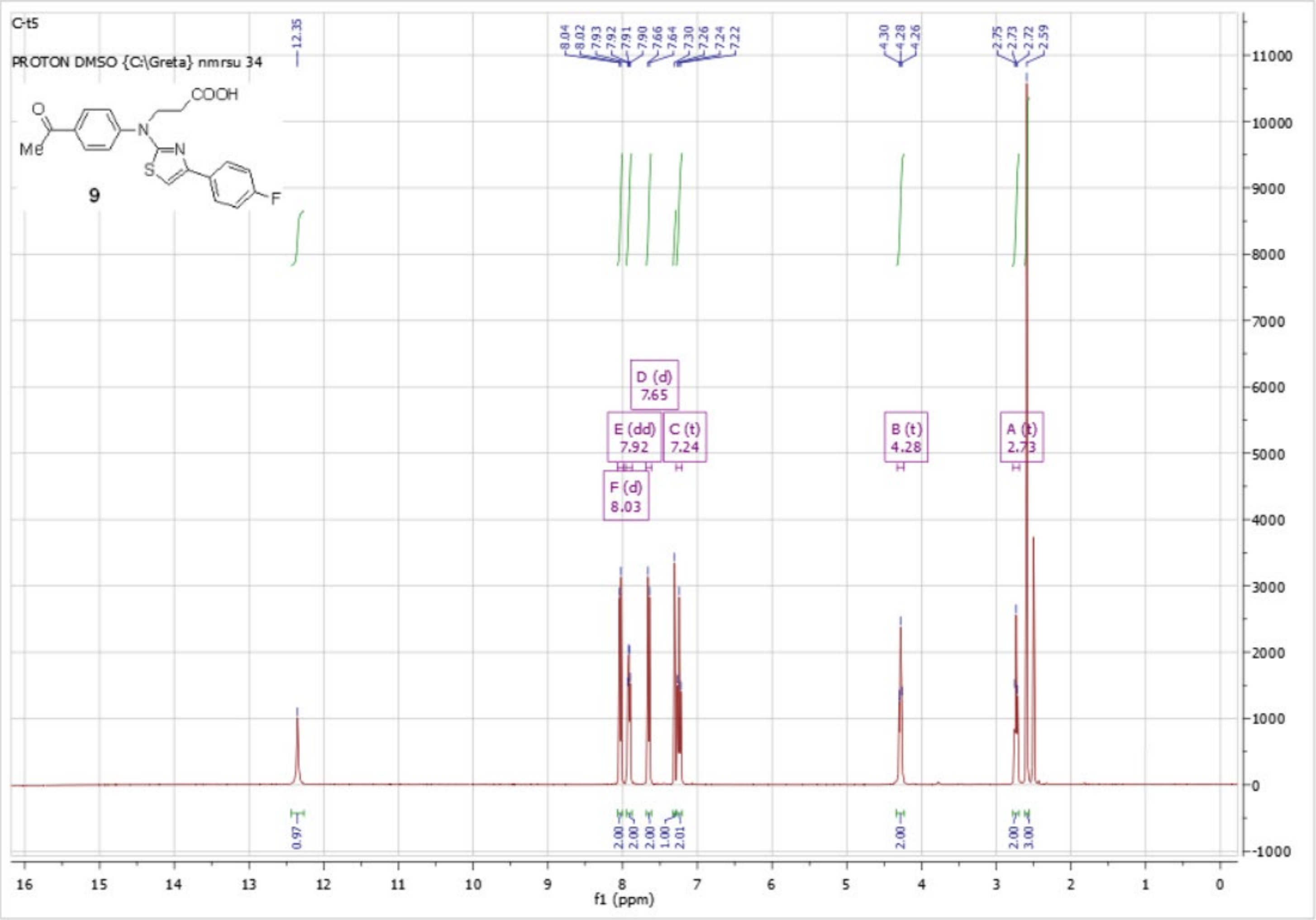

Figure S13. ^{1}H NMR spectrum of compound **9**.

*3-{(4-Acetylphenyl)[4-(4-fluorophenyl)thiazol-2-yl]amino}propanoic acid (**9**)*

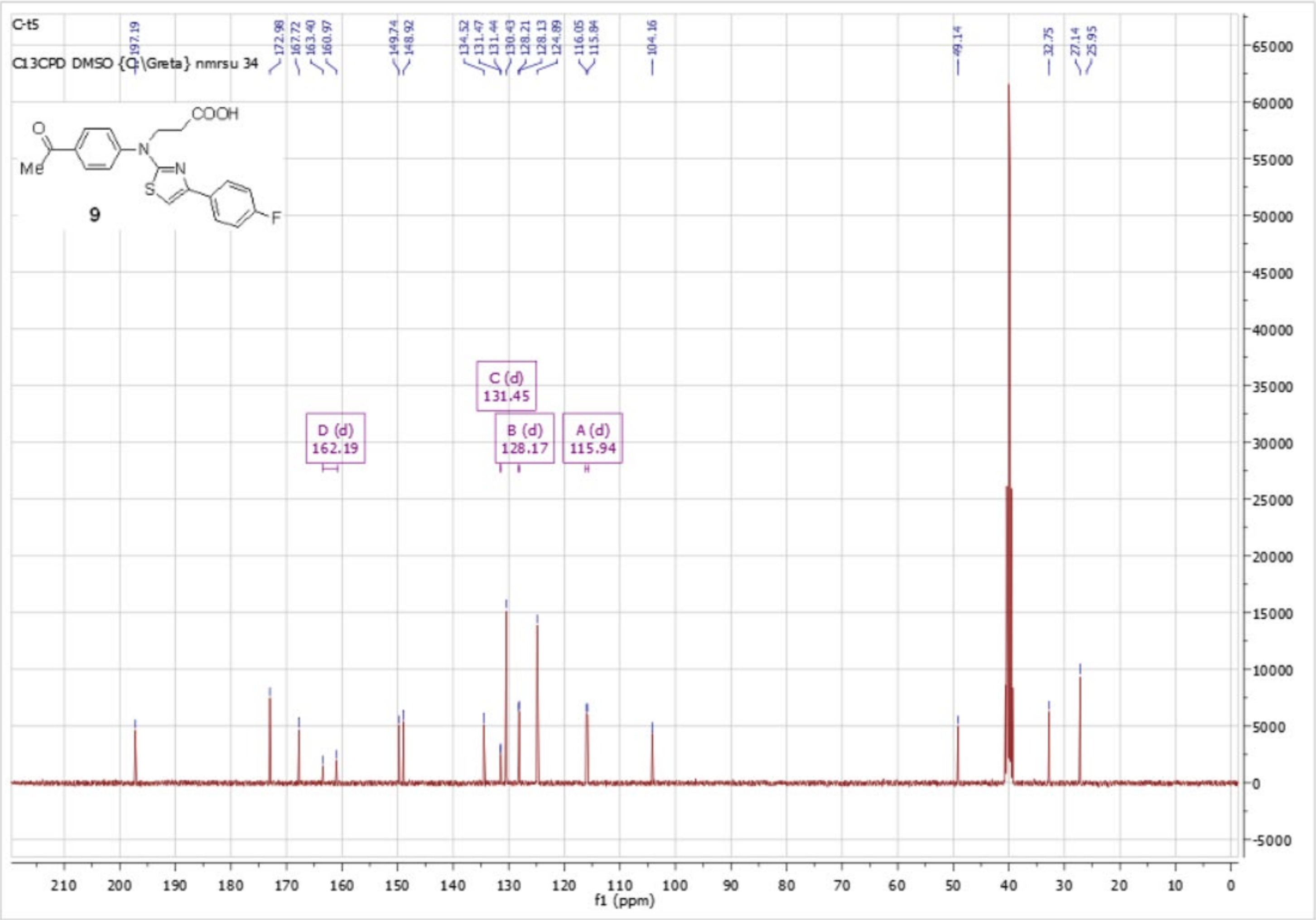

Figure S14. ^{13}C NMR spectrum of compound **9**.

*3-/(4-Acetylphenyl){4-[4-(trifluoromethyl)phenyl]thiazol-2-yl}amino/propanoic acid (**10**)*

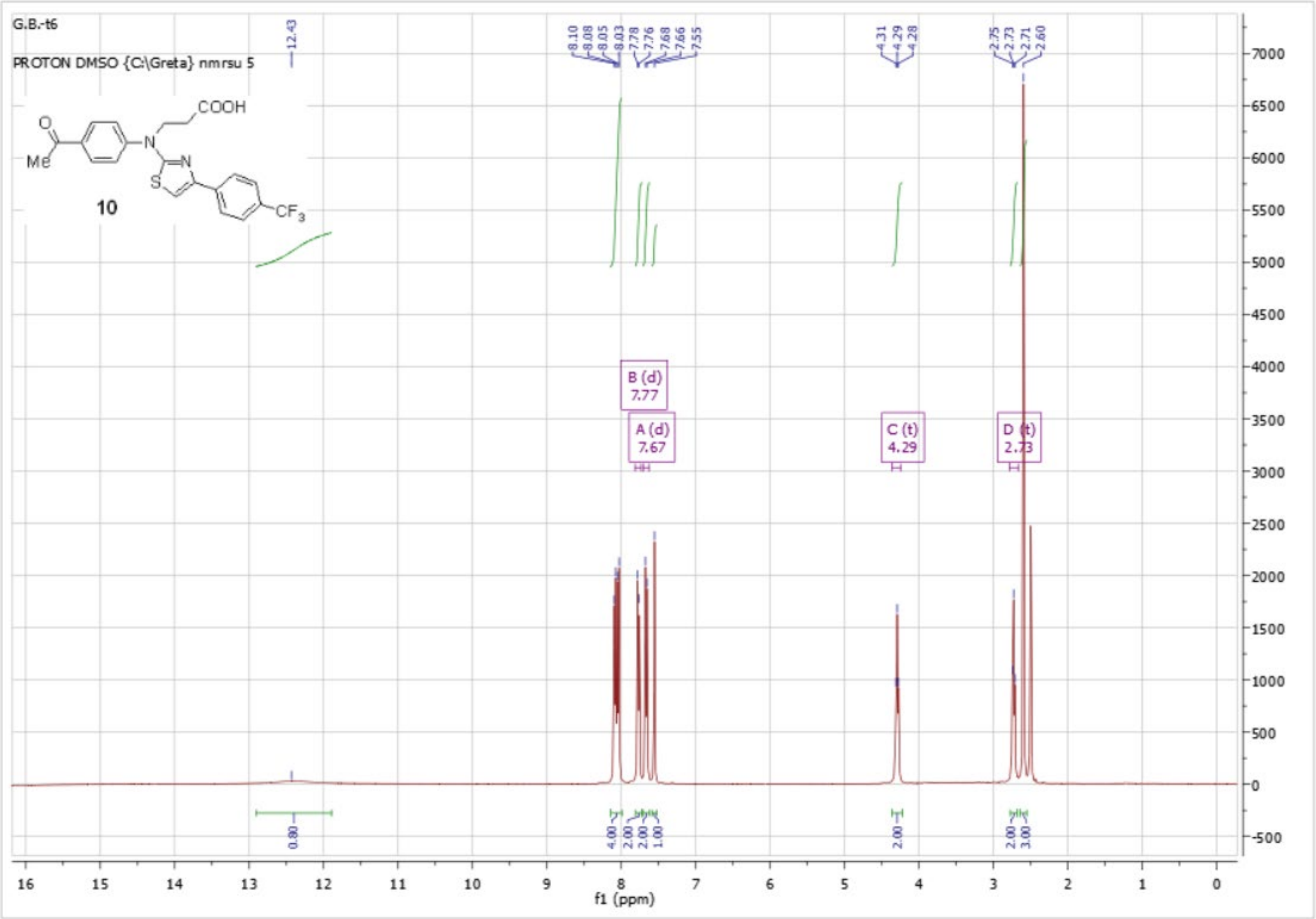

Figure S15. ^{1}H NMR spectrum of compound **10**.

*3-/(4-Acetylphenyl){4-[4-(trifluoromethyl)phenyl]thiazol-2-yl}amino/propanoic acid (**10**)*

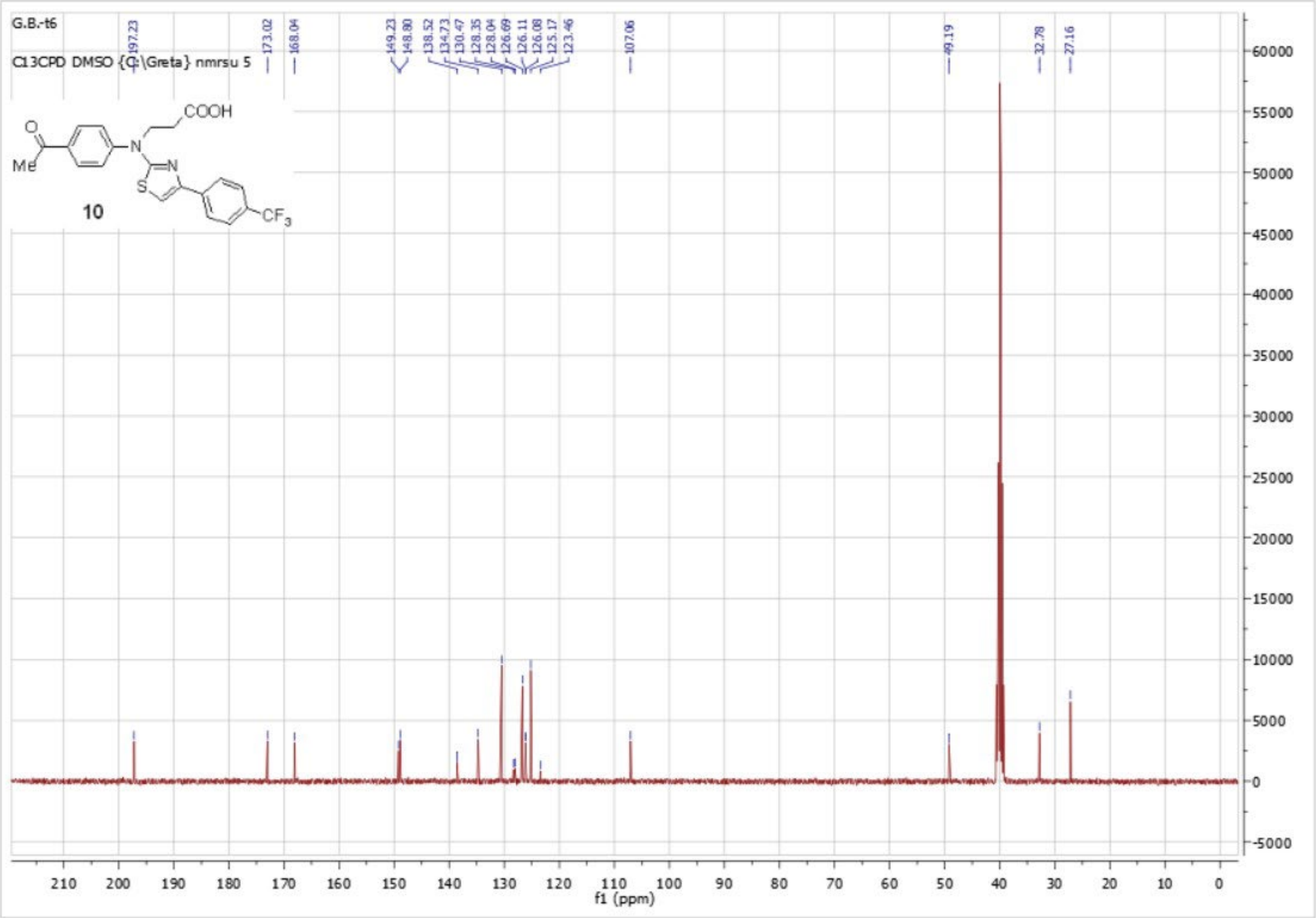

Figure S16. ^{13}C NMR spectrum of compound **10**.

*3-{(4-Acetylphenyl)[4-(4-hydroxyphenyl)thiazol-2-yl]amino}propanoic acid (**11**)*

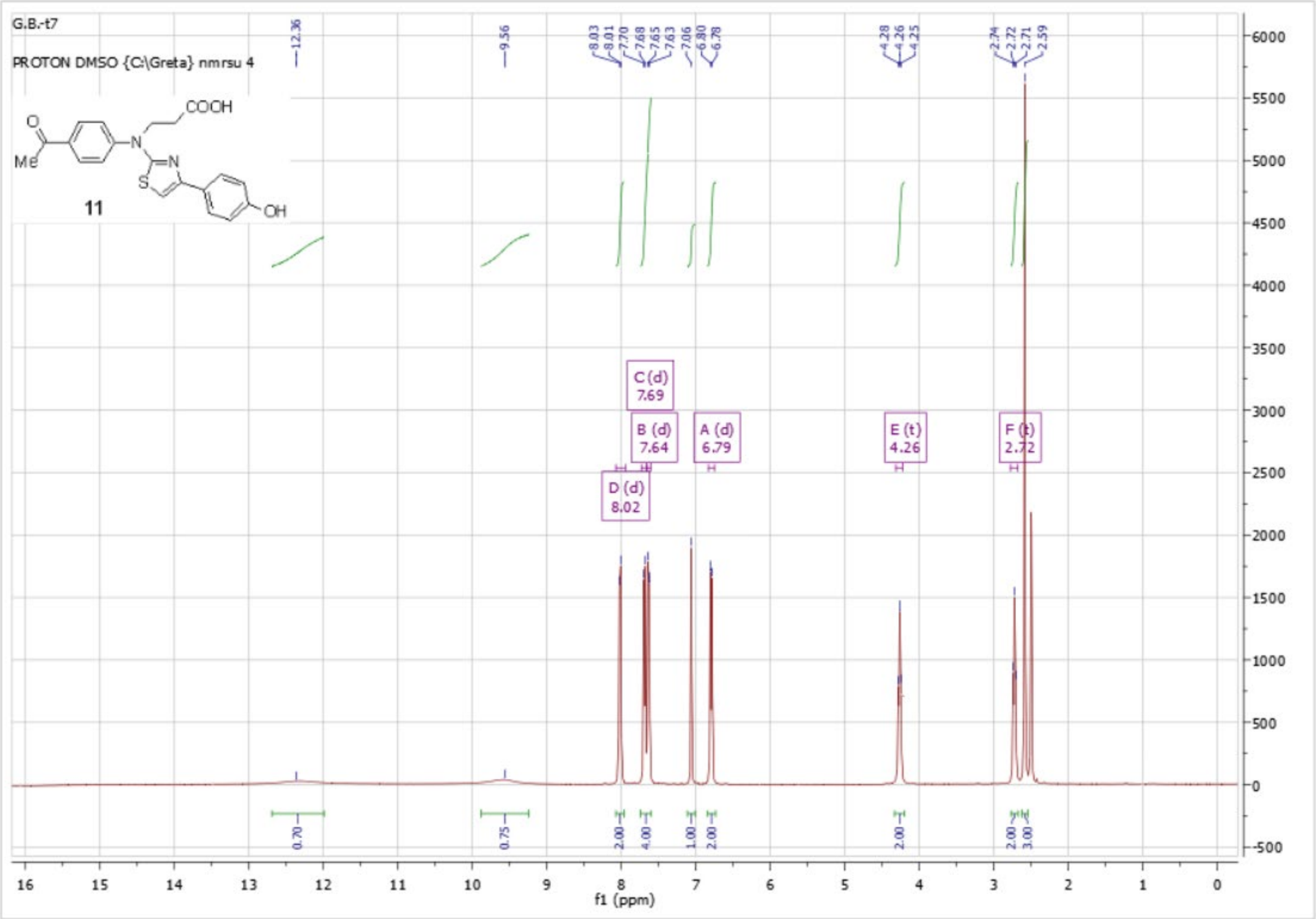

Figure S17. ¹H NMR spectrum of compound **11**.

*3-{(4-Acetylphenyl)[4-(4-hydroxyphenyl)thiazol-2-yl]amino}propanoic acid (**11**)*

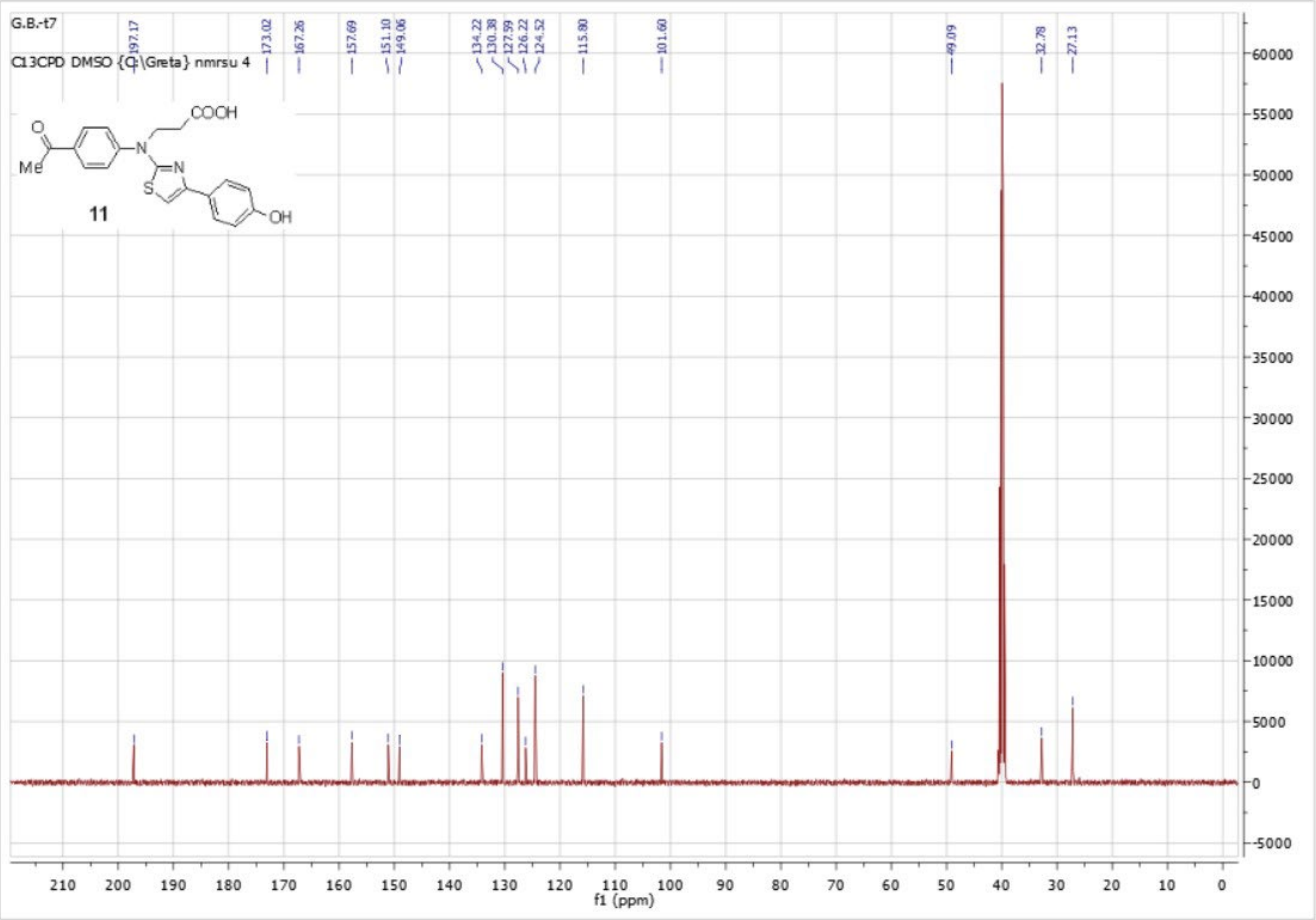

Figure S18. ^{13}C NMR spectrum of compound **11**.

*3-/(4-Acetylphenyl){4-[4-(trifluoromethoxy)phenyl]thiazol-2-yl}amino/propanoic acid (**12**)*

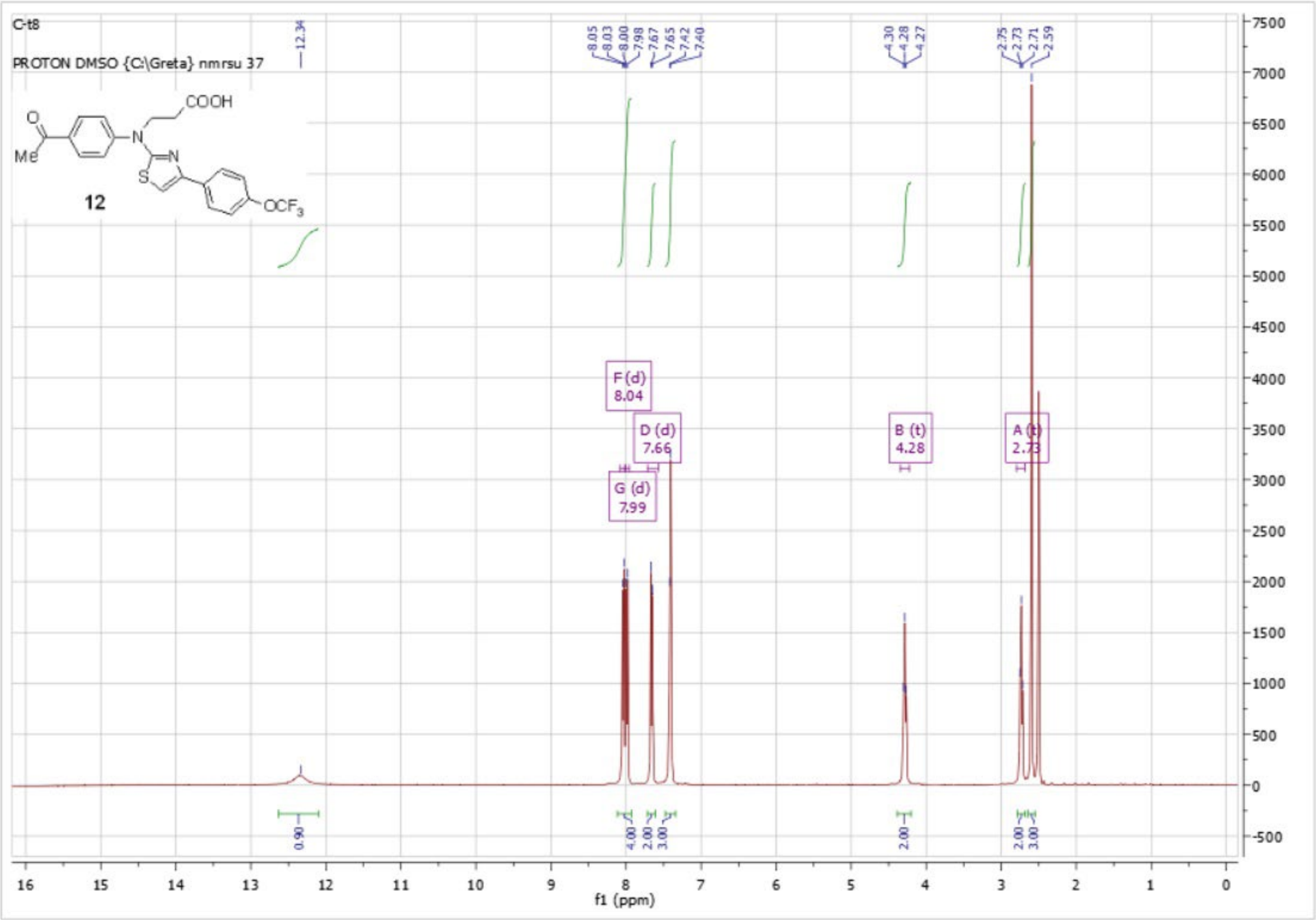

Figure S19. ^{1}H NMR spectrum of compound **12**.

*3-/(4-Acetylphenyl){4-[4-(trifluoromethoxy)phenyl]thiazol-2-yl}amino/propanoic acid (**12**)*

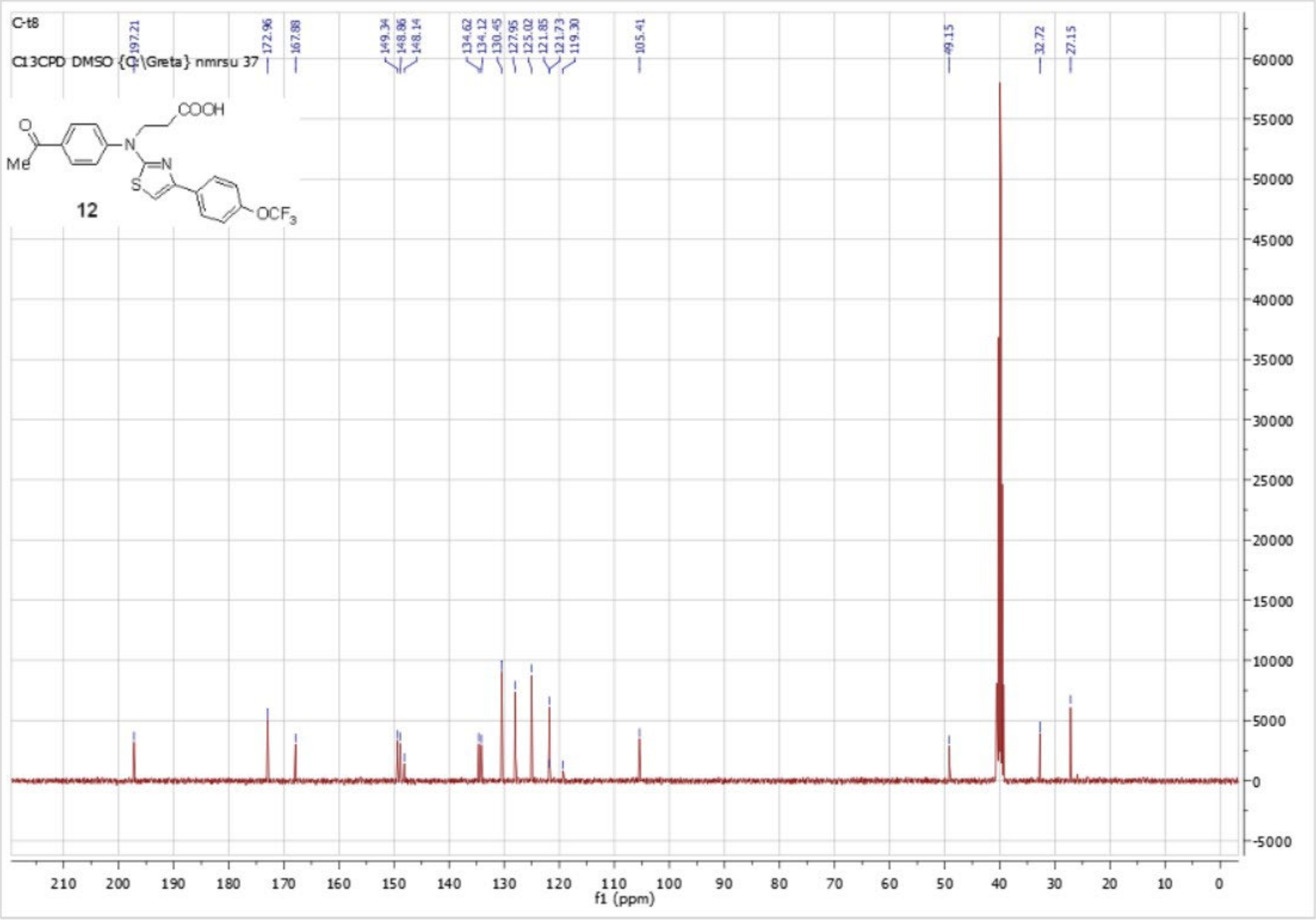

Figure S20. ^{13}C NMR spectrum of compound **12**.

*3,3′-{[(Phenylmethylene)bis(4-phenylthiazole-2,5-diyl)]bis[(4-acetylphenyl)azanediyl]}dipropionic acid (**13**)*

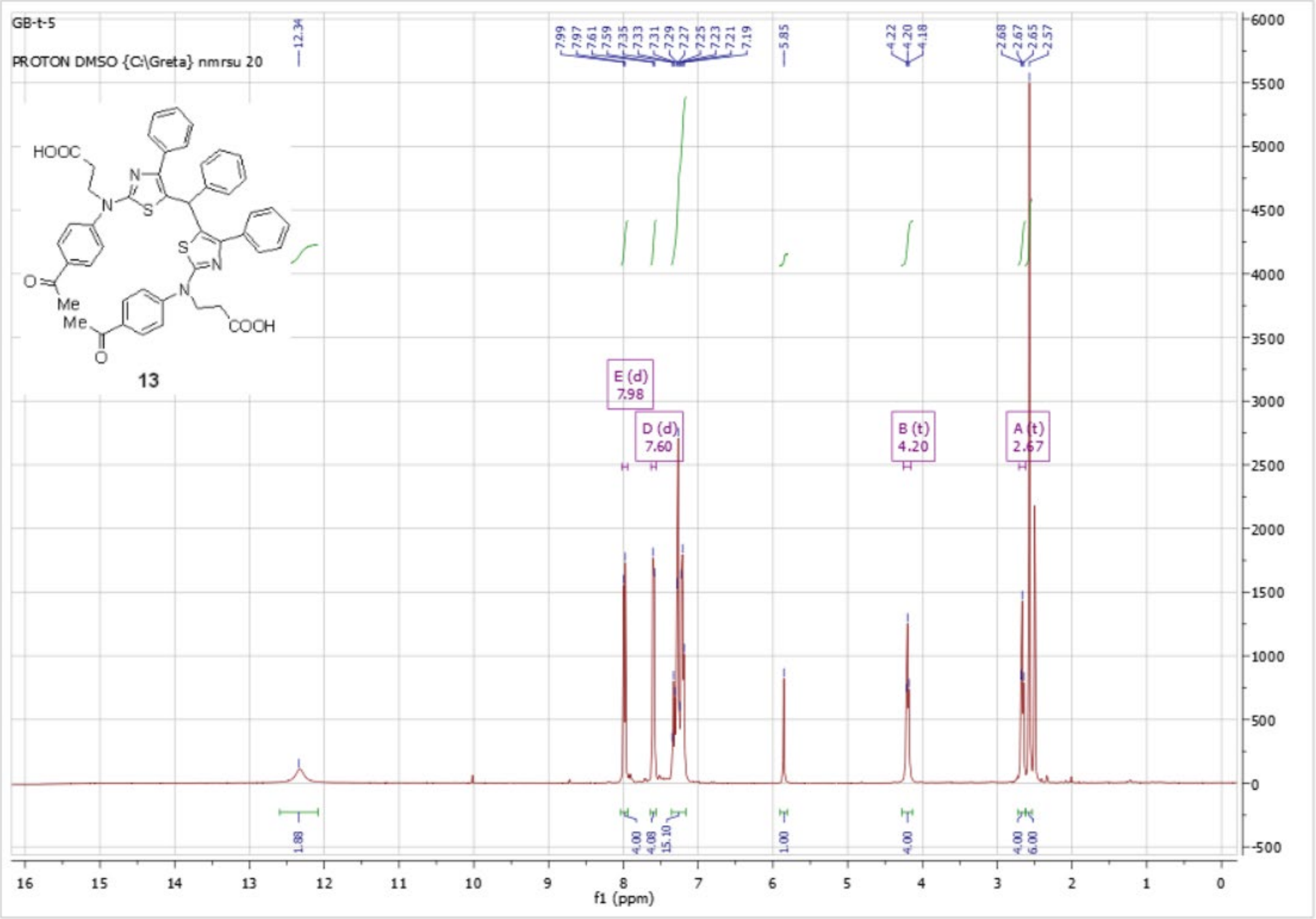

Figure S21. ^1H NMR spectrum of compound **13**.

*3,3′-{[(Phenylmethylene)bis(4-phenylthiazole-2,5-diyl)]bis[(4-acetylphenyl)azanediyl]}dipropionic acid (**13**)*

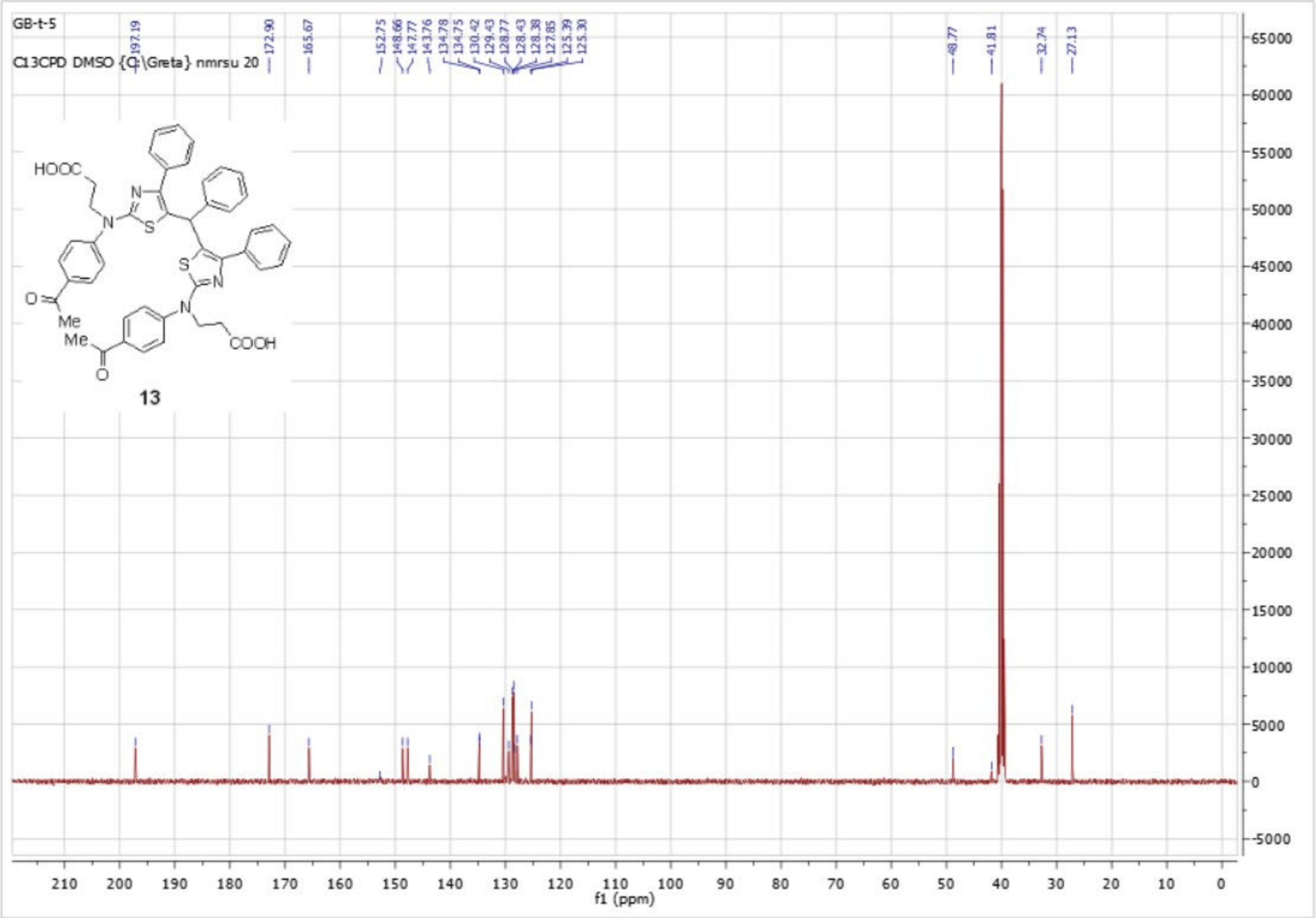

Figure S22. ^{13}C NMR spectrum of compound **13**.

*3,3′-/{[(4-Fluorophenyl)methylene]bis(4-phenylthiazole-5,2-diyl)}bis[(4-acetylphenyl)azanediyl]/dipropionic acid (**14**)*

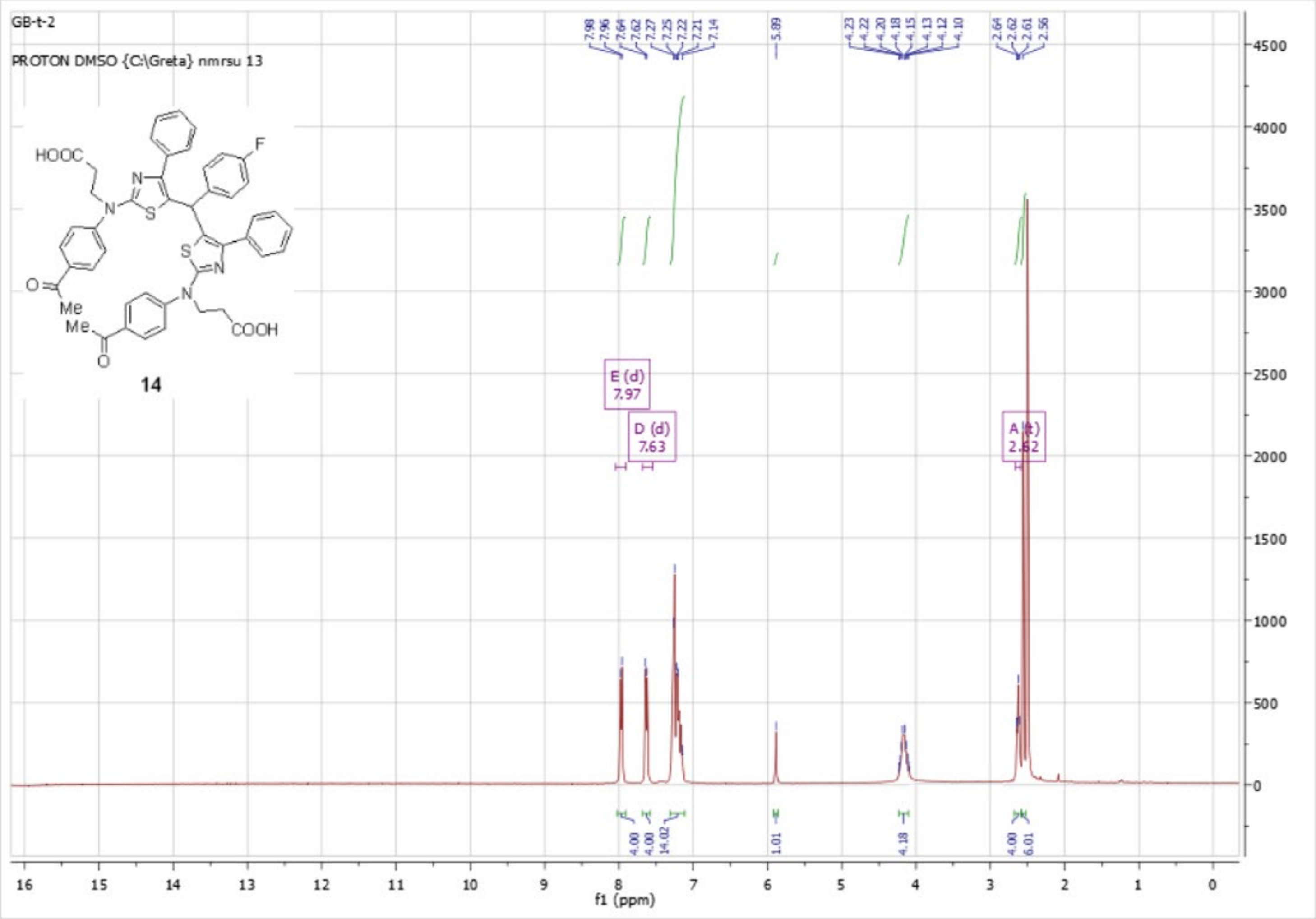

Figure S23. ^{1}H NMR spectrum of compound **14**.

*3,3′-/{[(4-Fluorophenyl)methylene]bis(4-phenylthiazole-5,2-diyl)}bis[(4-acetylphenyl)azanediyl]/dipropionic acid (**14**)*

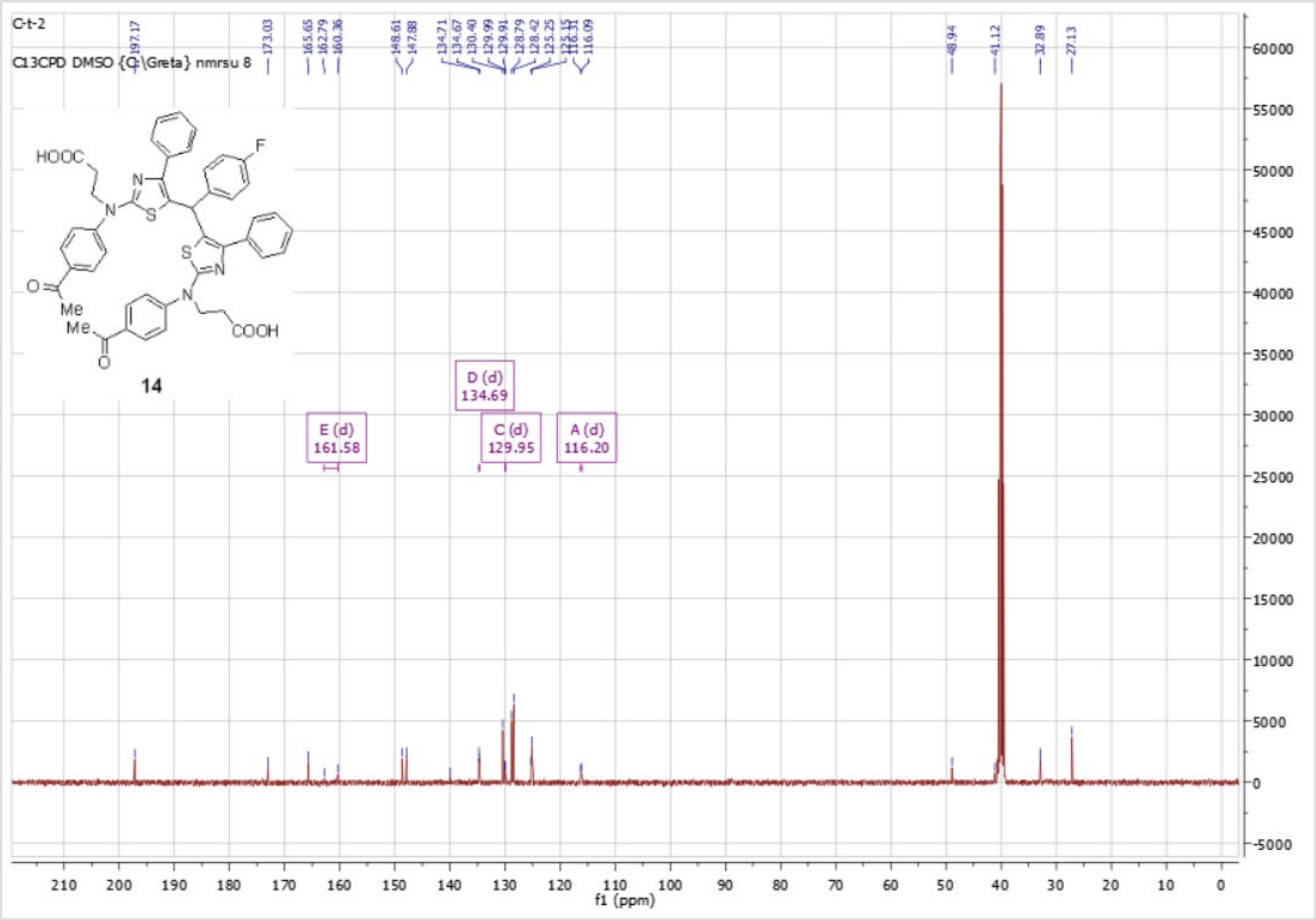

Figure S24. ^{13}C NMR spectrum of compound **14**.

*3,3′-/{[(4-Chlorophenyl)methylene]bis(4-phenylthiazole-5,2-diyl)}bis[(4-acetylphenyl)azanediyl]/dipropionic acid (**15**)*

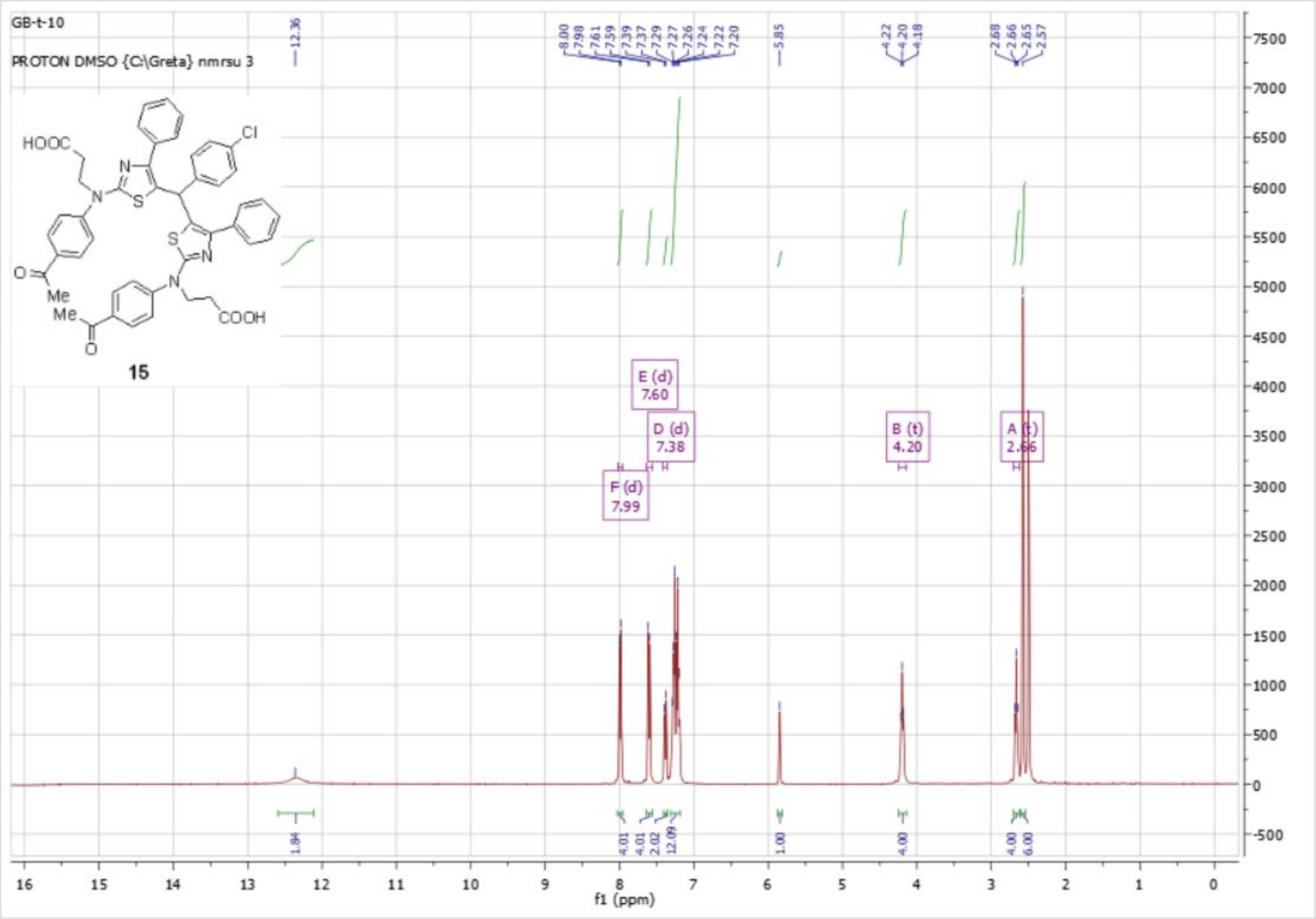

Figure S25. ^{1}H NMR spectrumof compound **15**.

*3,3′-/{[(4-Chlorophenyl)methylene]bis(4-phenylthiazole-5,2-diyl)}bis[(4-acetylphenyl)azanediyl]/dipropionic acid (**15**)*

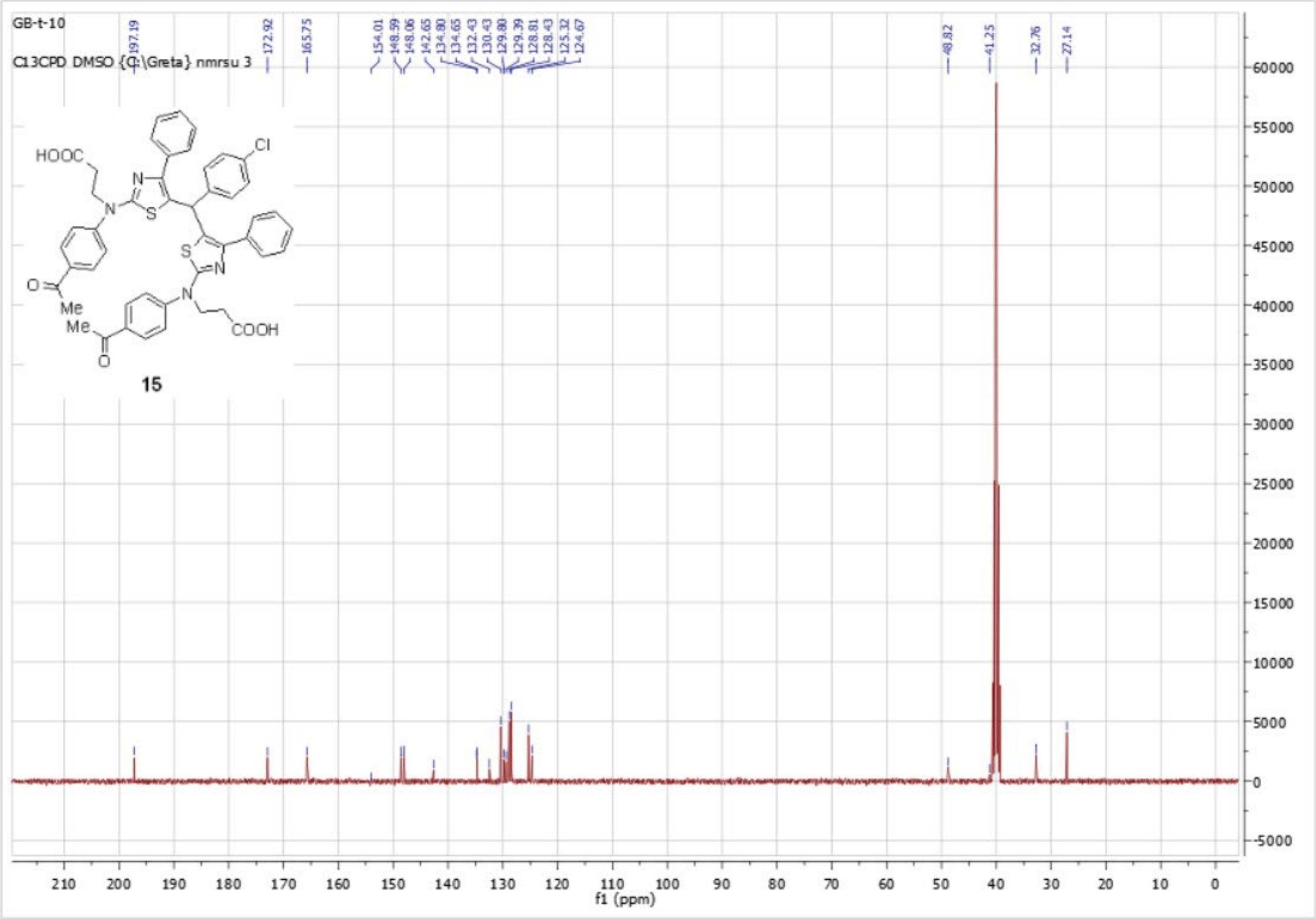

Figure S26. ^{13}C NMR spectrumof compound **15**.

*3,3′-/{[(2,4-Dimethoxyphenyl)methylene]bis(4-phenylthiazole-5,2-diyl)}bis[(4-acetylphenyl)azanediyl]/dipropionic acid (**16**)*

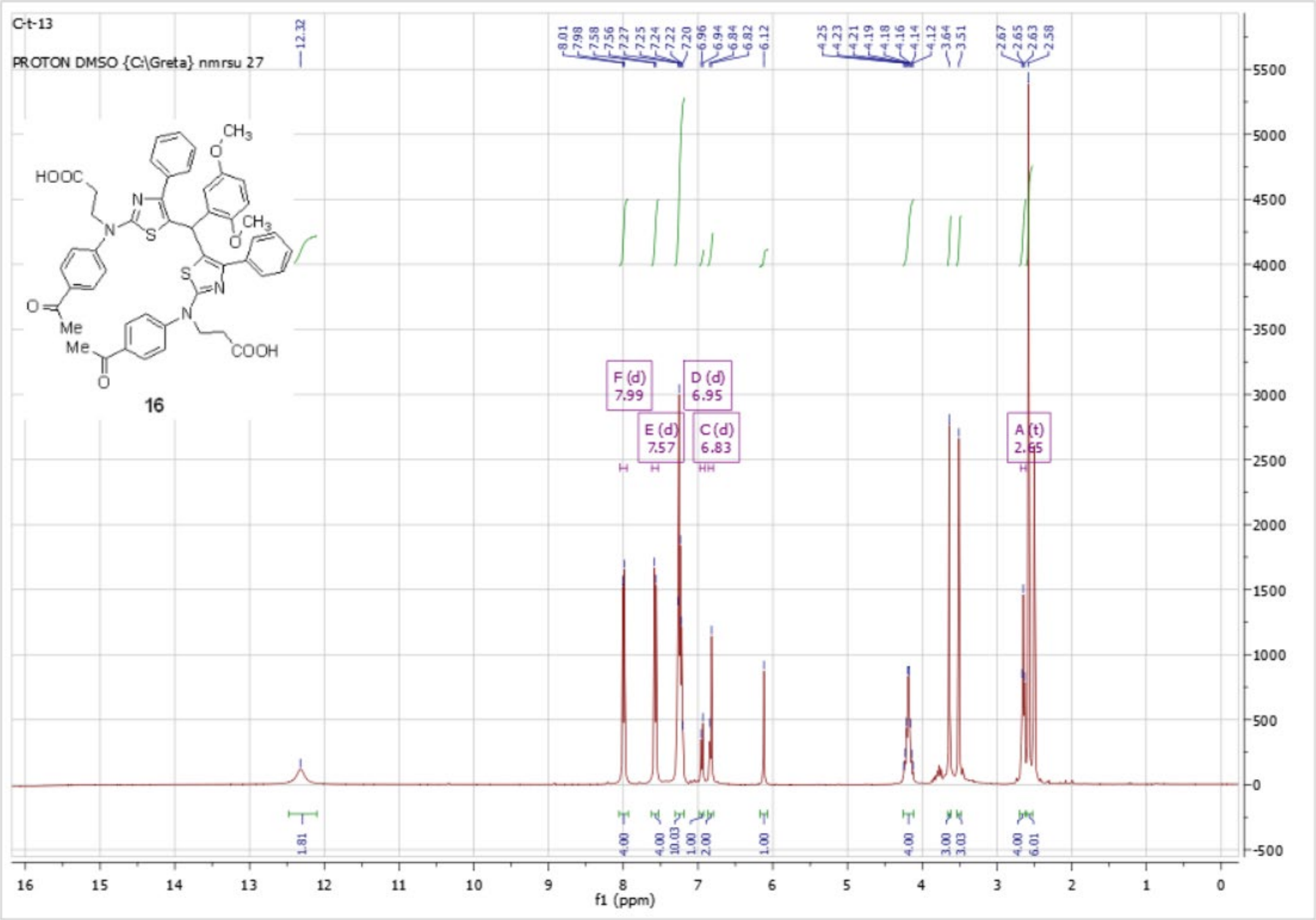

Figure S27. ^{1}H NMR spectrumof compound **16**.

*3,3′-/{[(2,4-Dimethoxyphenyl)methylene]bis(4-phenylthiazole-5,2-diyl)}bis[(4-acetylphenyl)azanediyl]/dipropionic acid (**16**)*

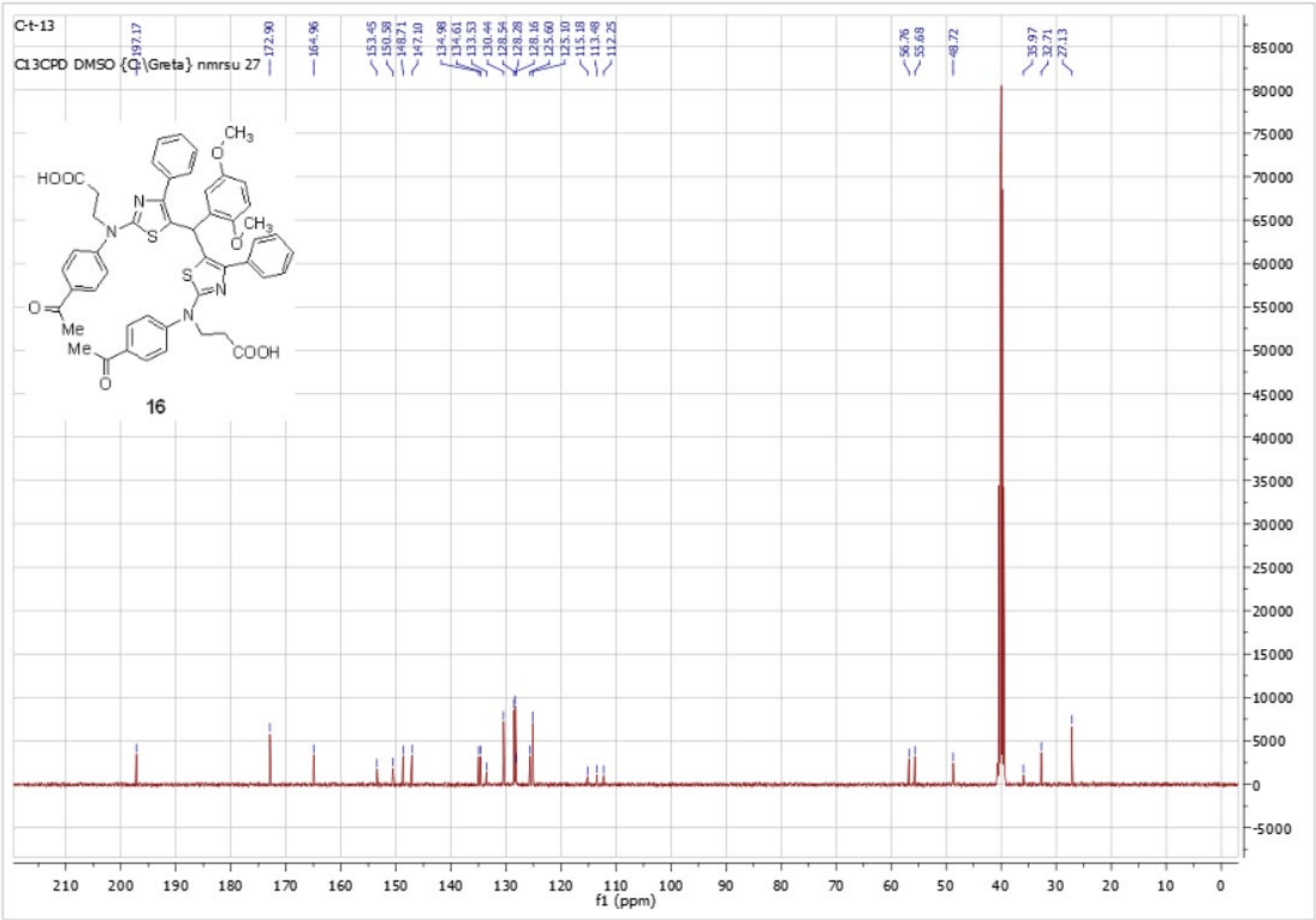

Figure S28. ^{13}C NMR spectrumof compound **16**.

*3,3′-/{[(4-Fluorophenyl)methylene]bis[4-(4-chlorophenyl)thiazole-5,2-diyl]}bis](4-acetylphenyl)azanediyl]/dipropionic acid (**17**)*

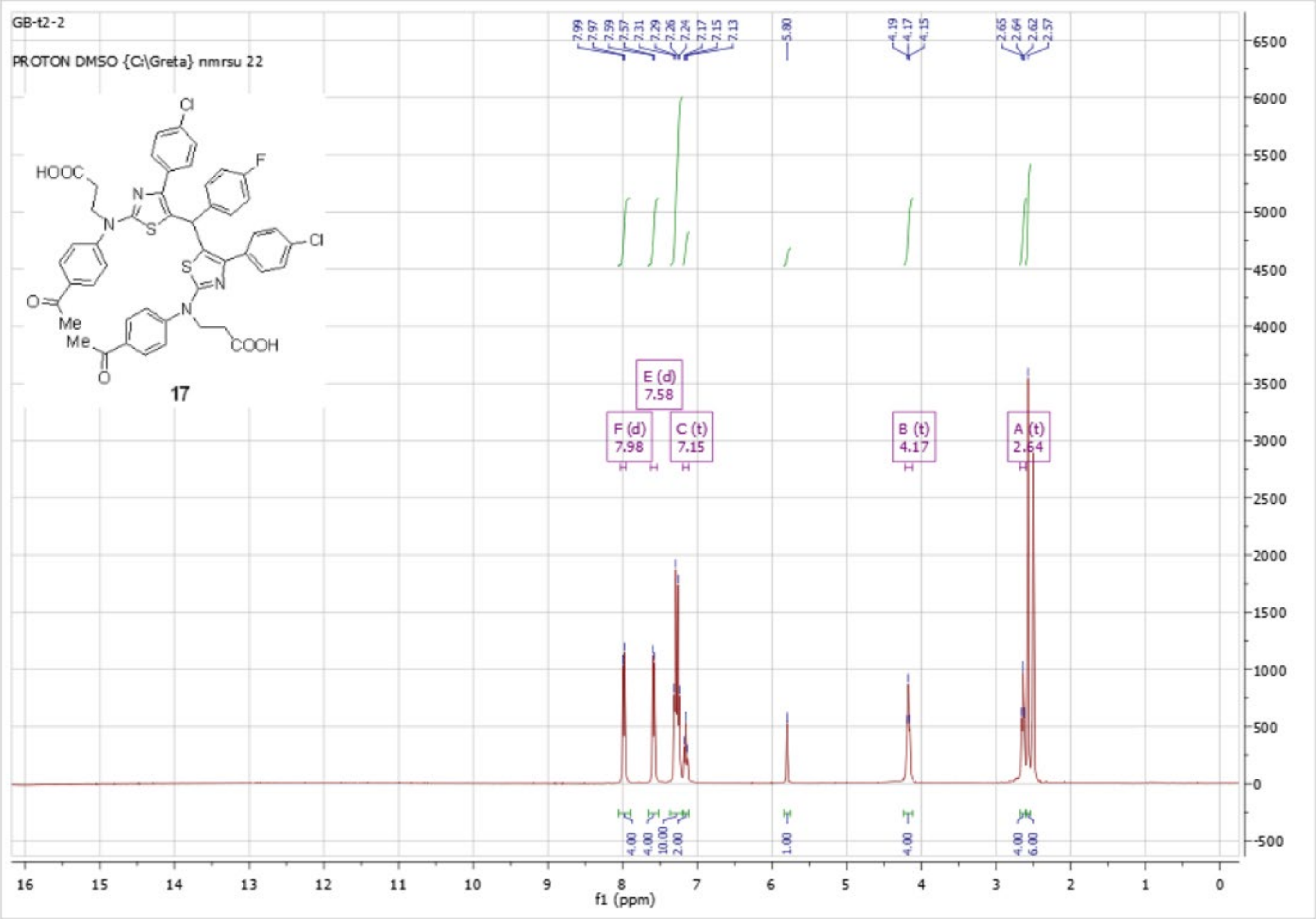

Figure S29. ^{1}H NMRspectrum of compound **17.**

*3,3′-/{[(4-Fluorophenyl)methylene]bis[4-(4-chlorophenyl)thiazole-5,2-diyl]}bis](4-acetylphenyl)azanediyl]/dipropionic acid (**17**)*

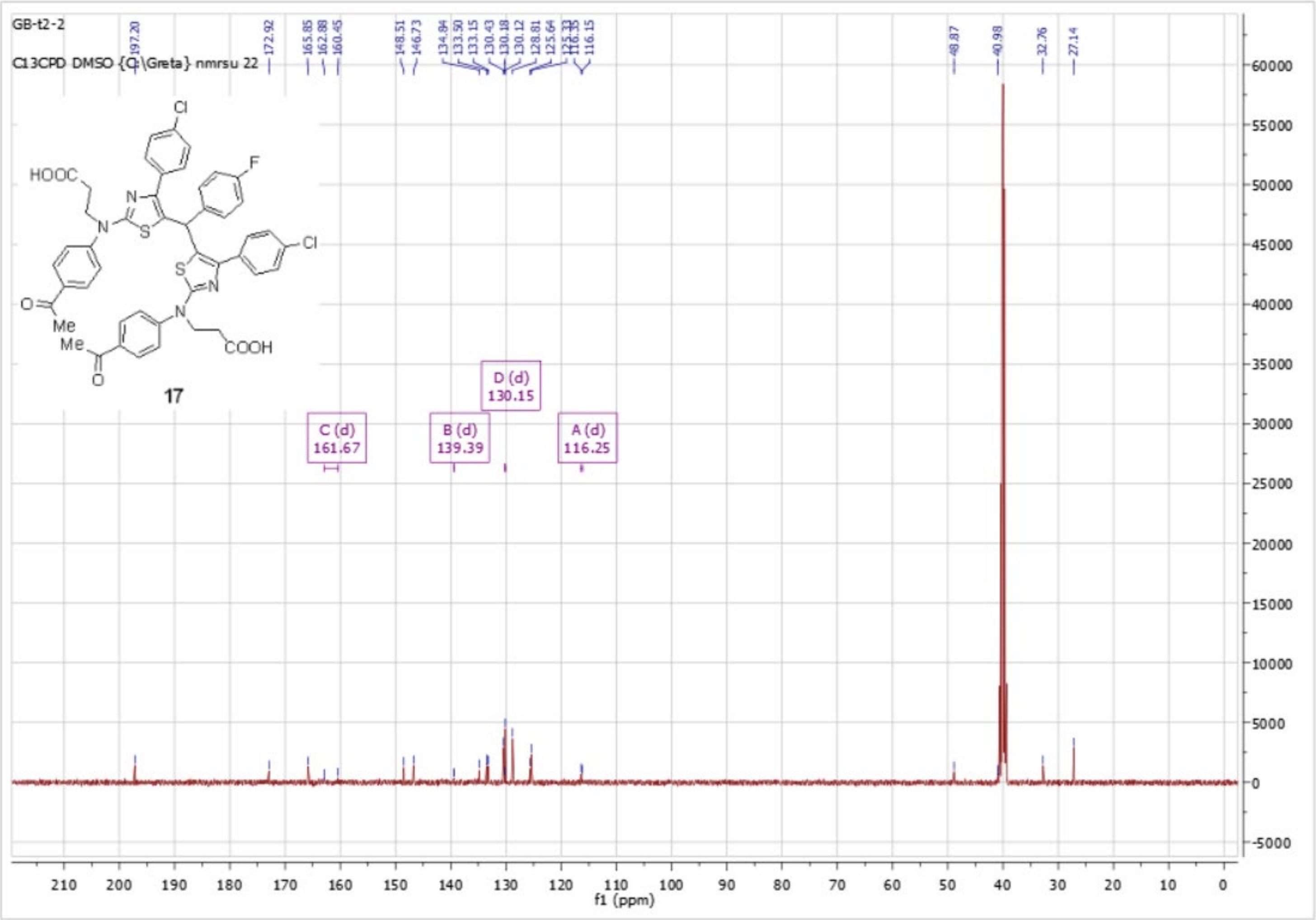

Figure S30. ^{13}C NMRspectrum of compound **17.**

*3-/{4-[3-(4-Fluorophenyl)acryloyl]phenyl}(4-phenylthiazol-2-yl)amino/propanoic acid (**18**)*

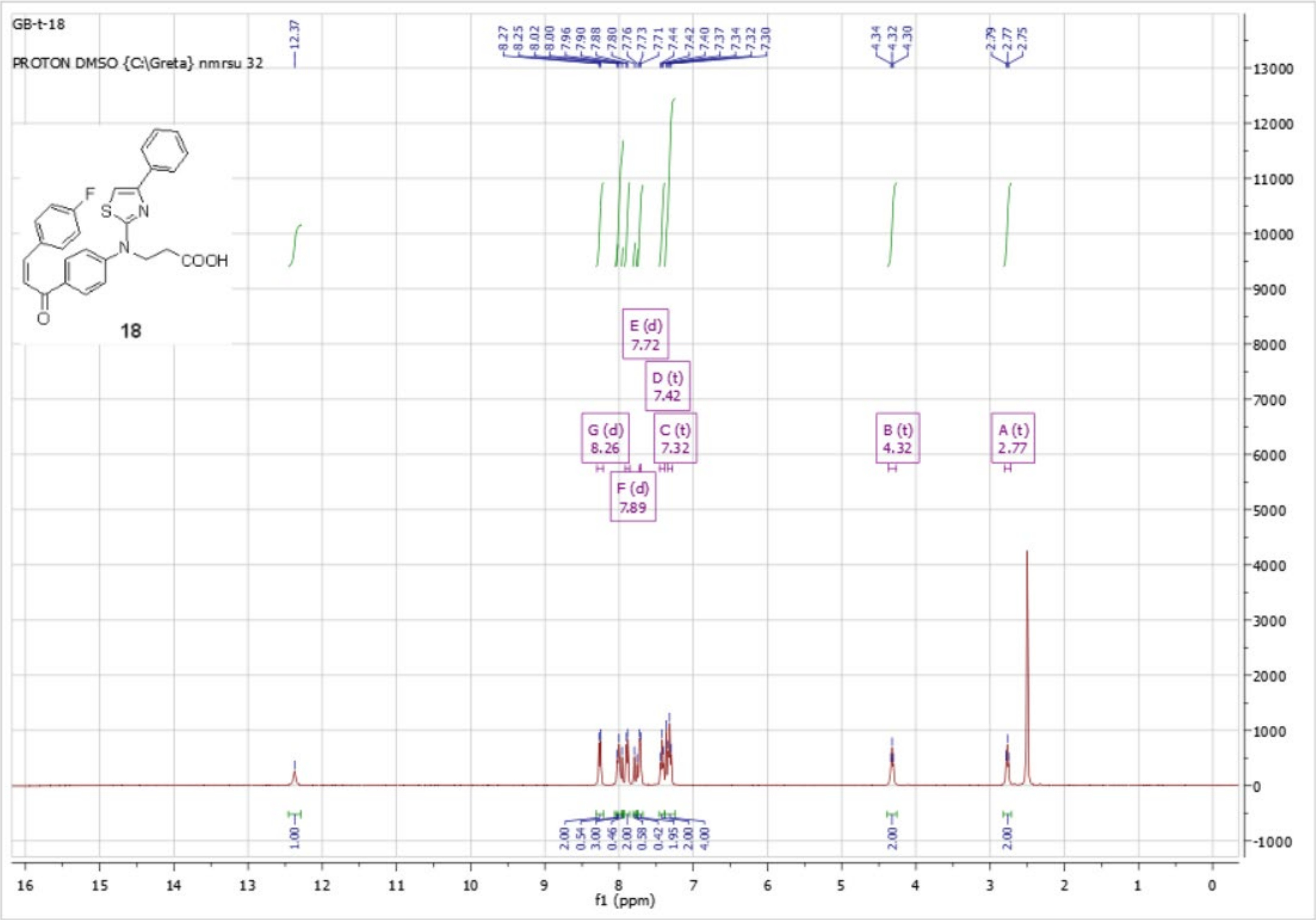

Figure S31. ^{1}H NMR spectrumof compound **18.**

*3-/{4-[3-(4-Fluorophenyl)(4-phenylthiazol-2-yl)amino/propanoic acid (**18**)*

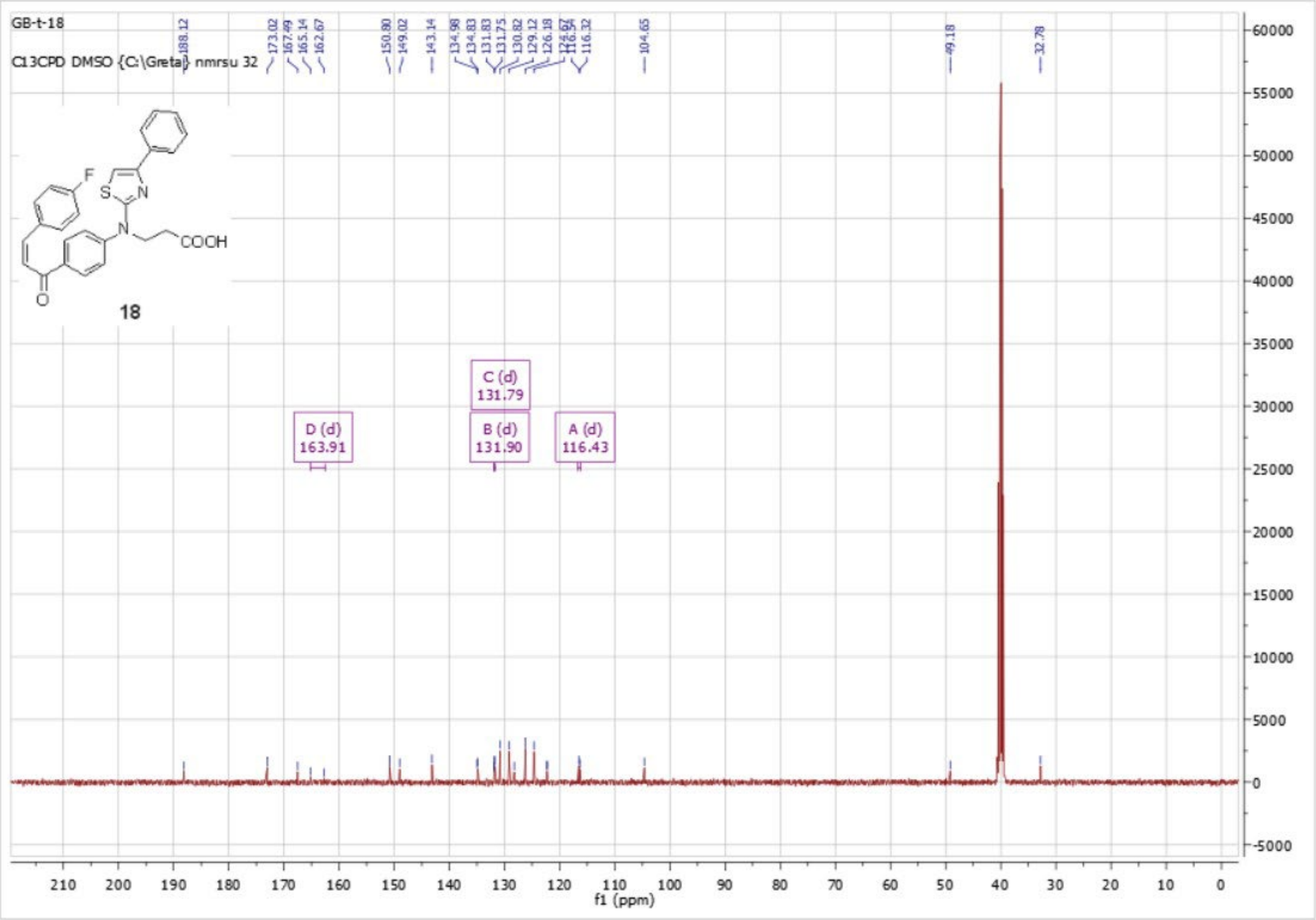

Figure S32. ^{13}C NMR spectrumof compound **18**.

*3-/[4-(4-Chlorophenyl)thiazol-2-yl] {4-[3-(4-fluorophenyl)acryloyl]phenyl}amino/propanoic acid (**19**)*

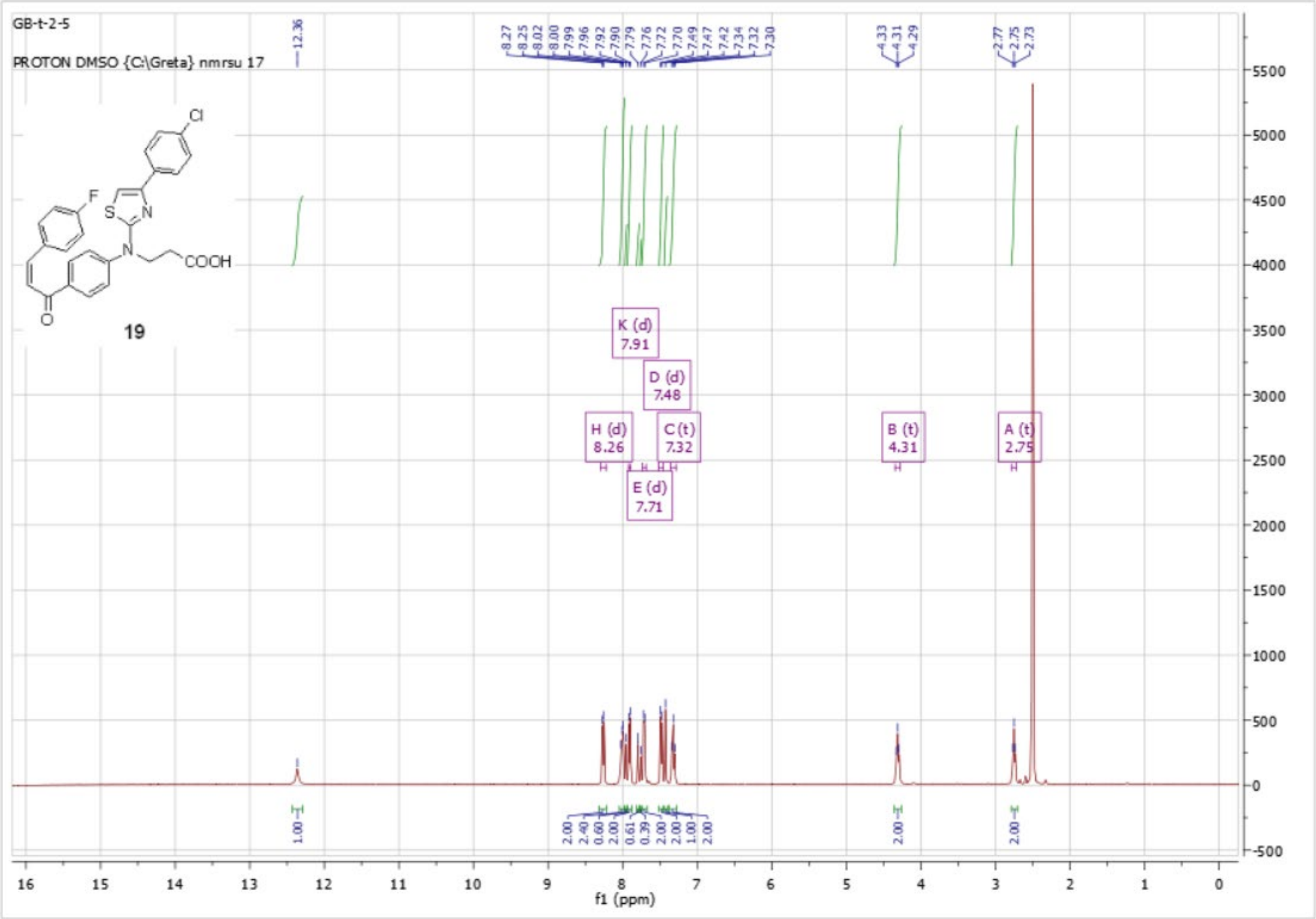

Figure S33. ¹H NMR spectrumof compound **19**.

*3-/[4-(4-Chlorophenyl)thiazol-2-yl] {4-[3-(4-fluorophenyl)acryloyl]phenyl}amino/propanoic acid (**19**)*

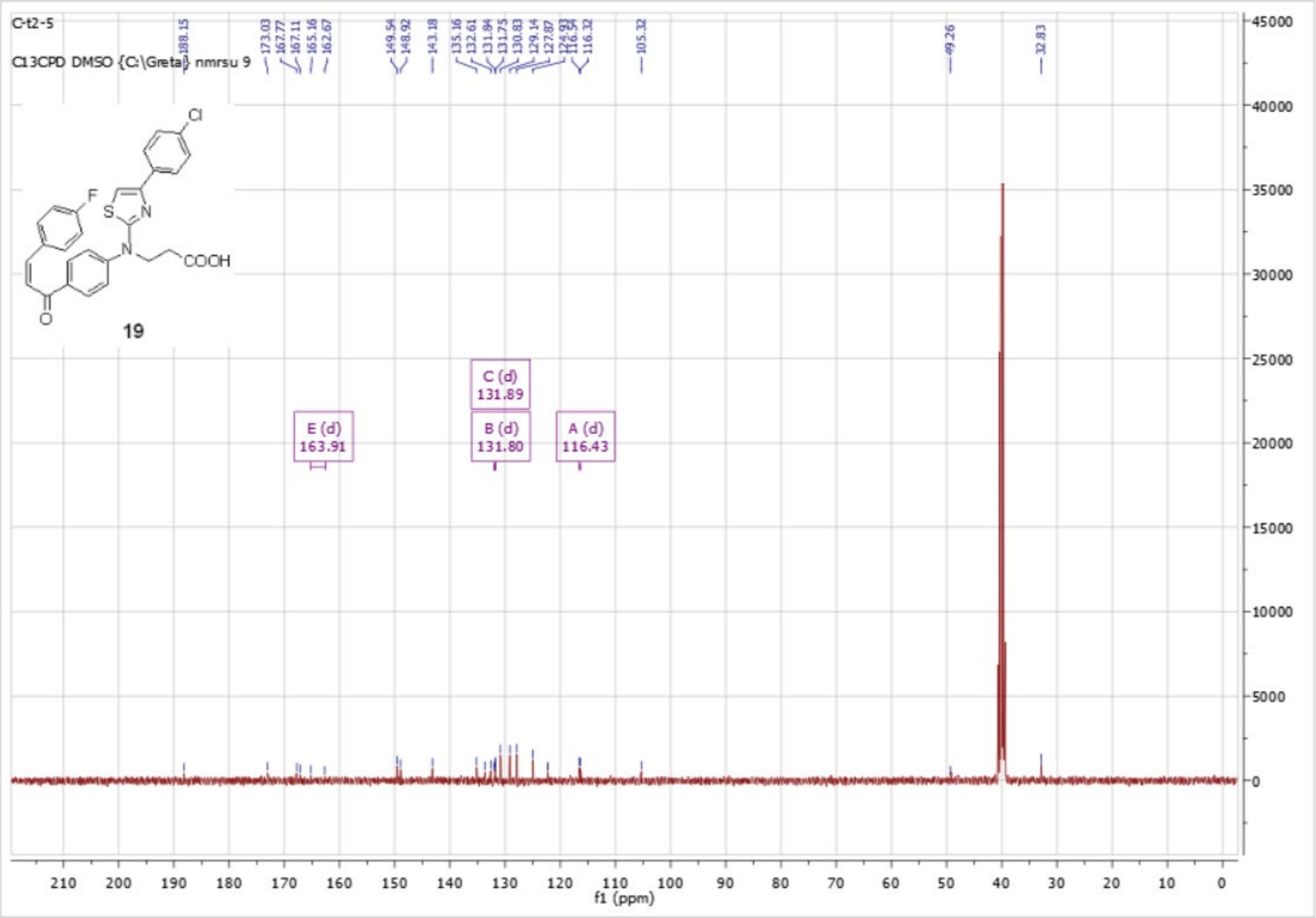

Figure S34. ^{13}C NMR spectrumof compound **19**.

*3-/{4-[3-(4-Fluorophenyl)acryloyl]phenyl}{4-[4-(trifluoromethoxy)phenyl]thiazol-2-yl}amino/propanoic acid (**20**)*

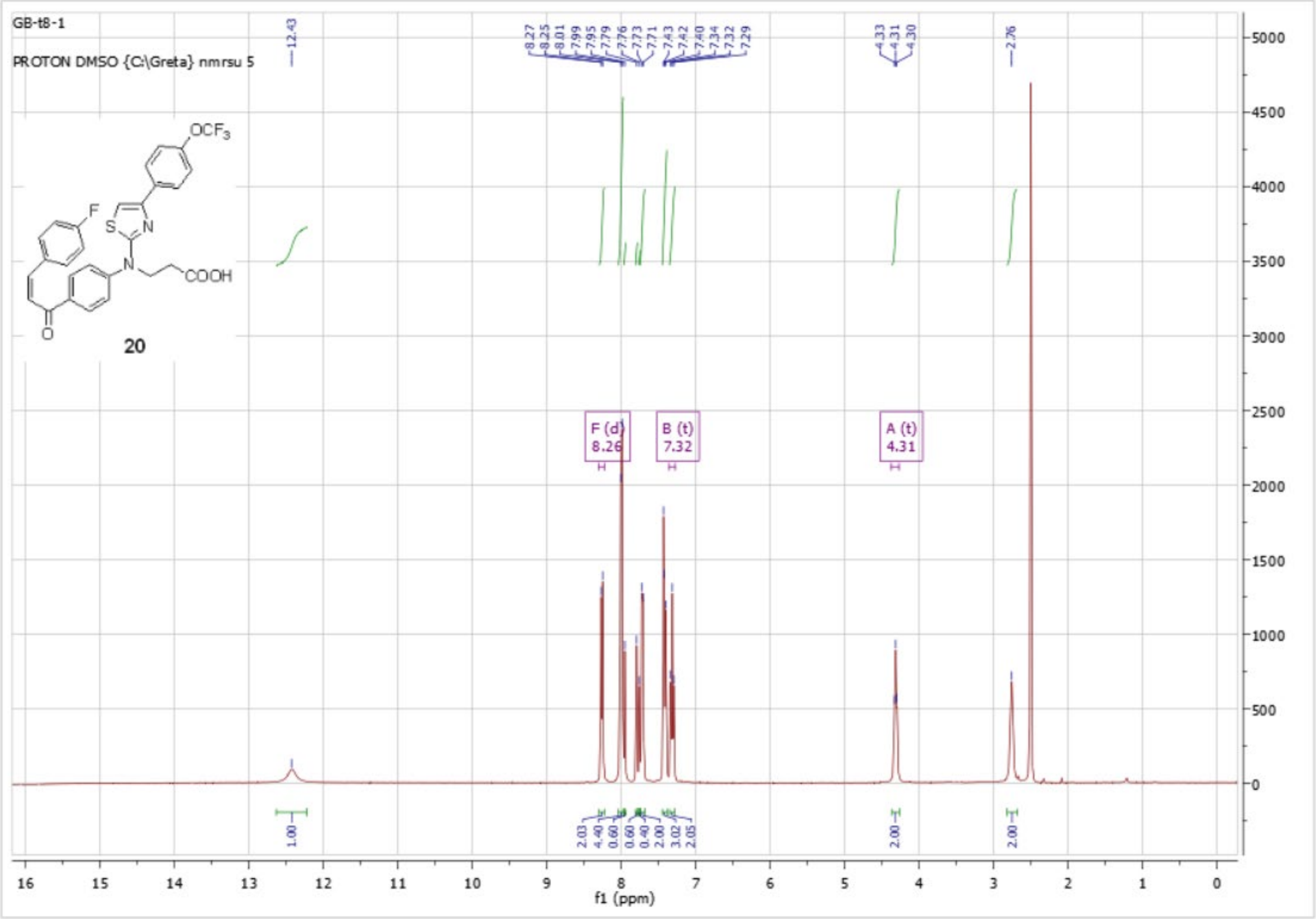

Figure S35. ¹H NMR spectrumof compound **20**.

*3-/{4-[3-(4-Fluorophenyl)acryloyl]phenyl}{4-[4-(trifluoromethoxy)phenyl]thiazol-2-yl}amino/propanoic acid (**20**)*

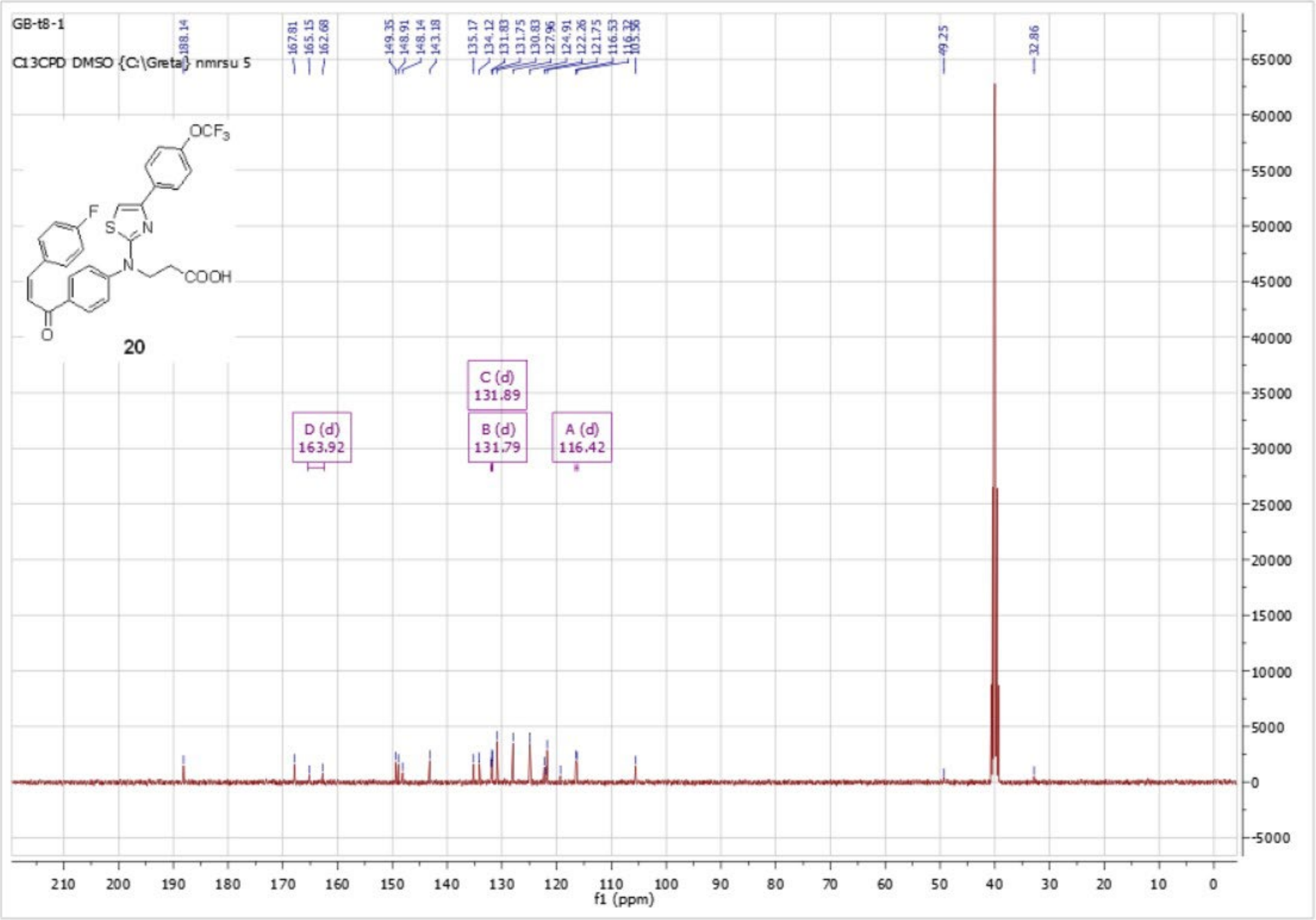

Figure S36. ^{13}C NMR spectrumof compound **20**.

*3-/{4-[1-(Hydroxyimino)ethyl]phenyl}(4-phenylthiazol-2-yl)amino/propanoic acid (**21**)*

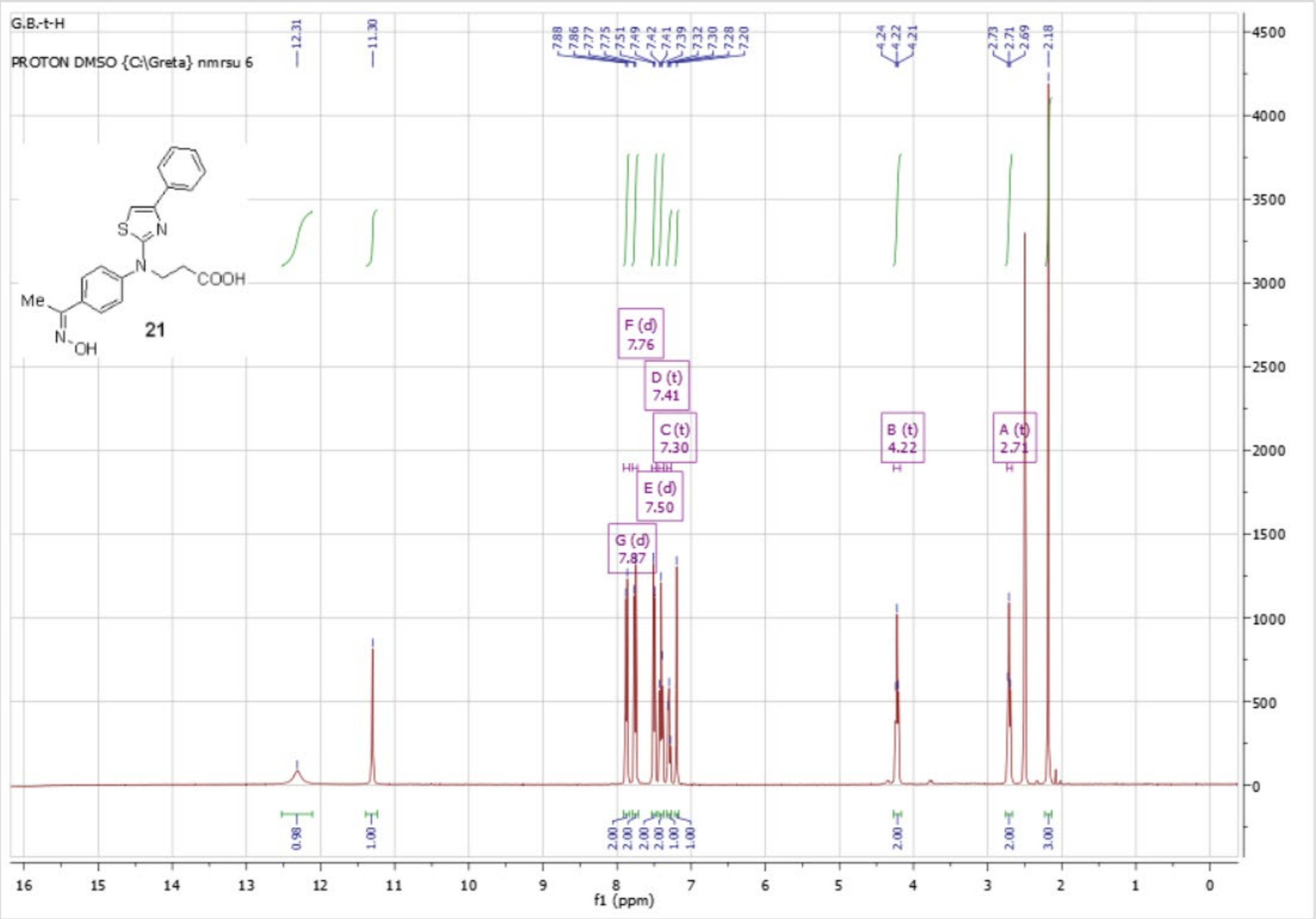

Figure S37.¹H NMR spectrumof compound **21**.

*3-/{4-[1-(Hydroxyimino)ethyl]phenyl}(4-phenylthiazol-2-yl)amino/propanoic acid (**21**)*

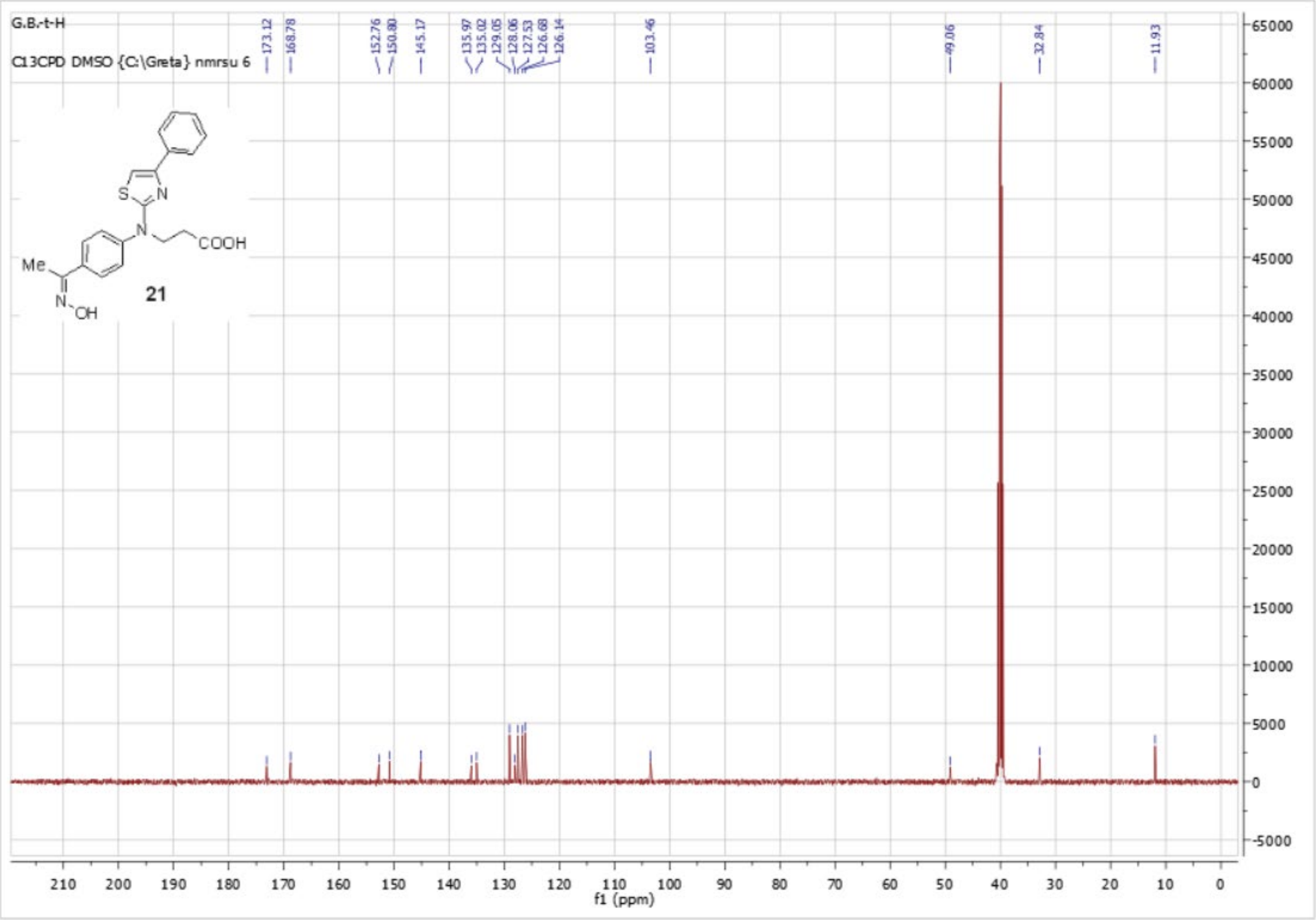

Figure S38. ^{13}C NMR spectrumof compound **21**.

*3-/[4-(4-Chlorophenyl)thiazol-2-yl]{4-[1-(hydroxyimino)ethyl]phenyl}amino/propanoic acid (**22**)*

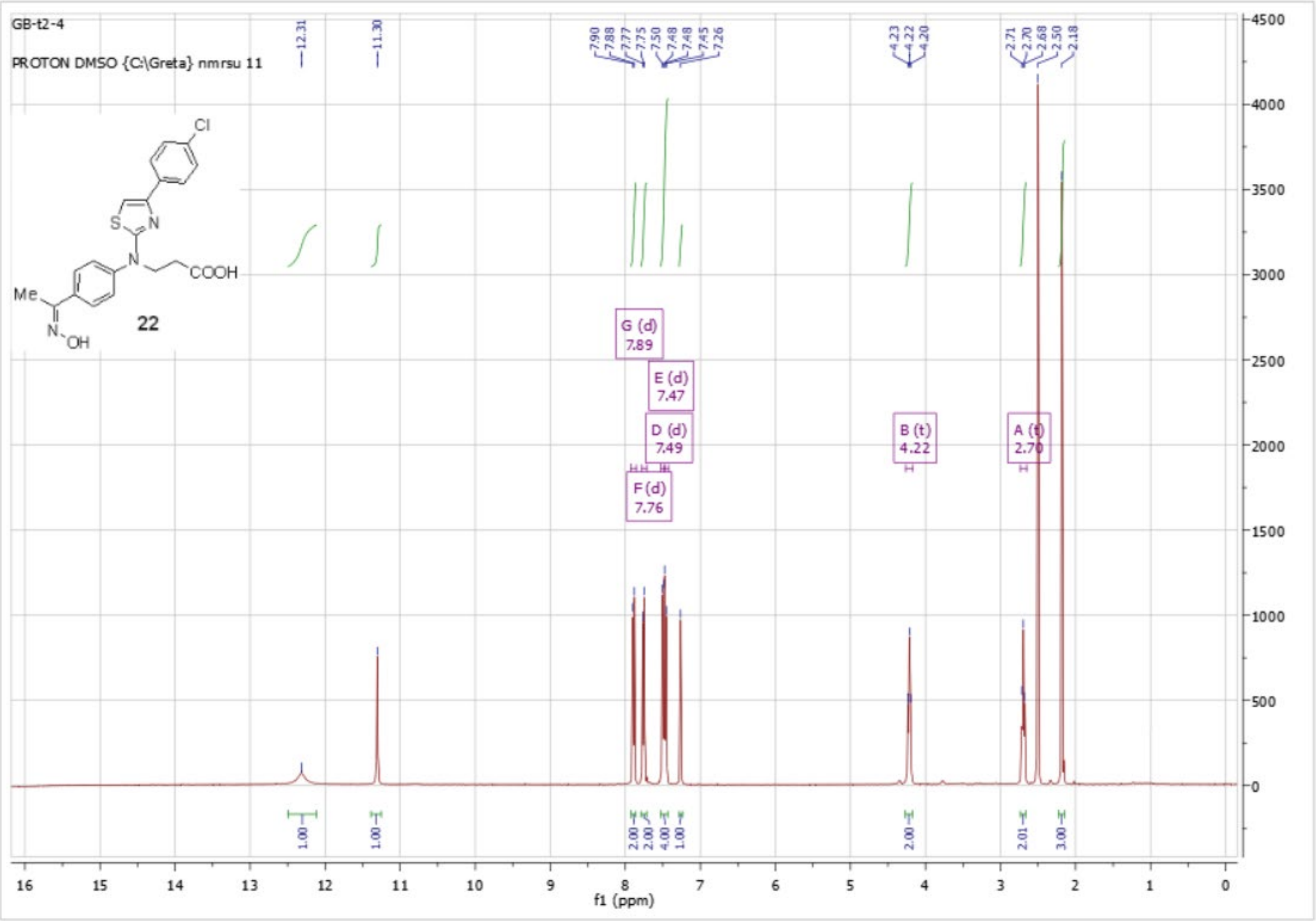

Figure S39. ^{1}H NMR spectrum of compound **22**.

*3-/[4-(4-Chlorophenyl)thiazol-2-yl]{4-[1-(hydroxyimino)ethyl]phenyl}amino/propanoic acid (**22**)*

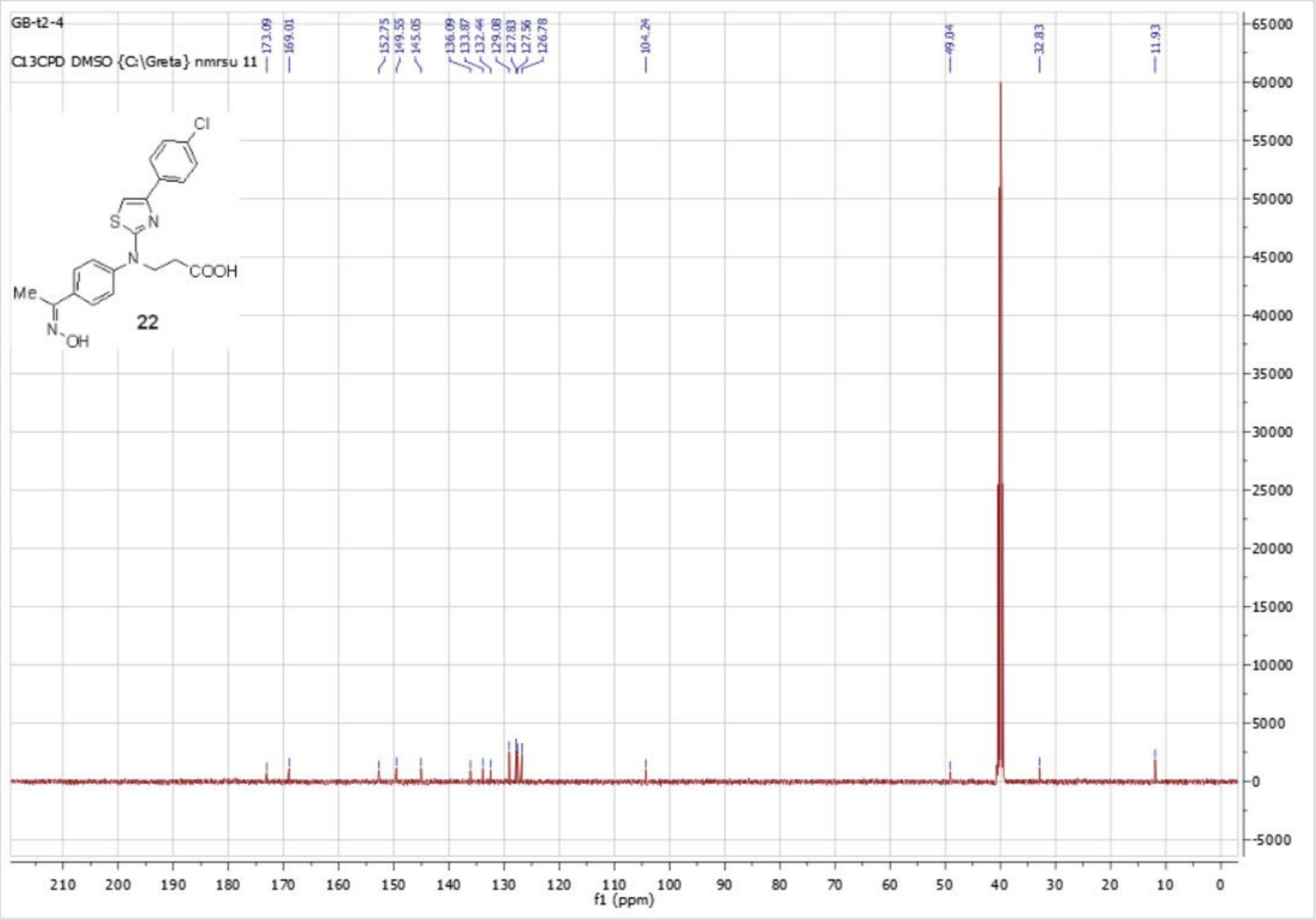

Figure S40. ^{13}C NMR spectrum of compound **22**.

*Methyl 3-/{4-[1-(hydroxyimino)ethyl]phenyl}(4-phenylthiazol-2-yl)amino/propanoate (**23**)*

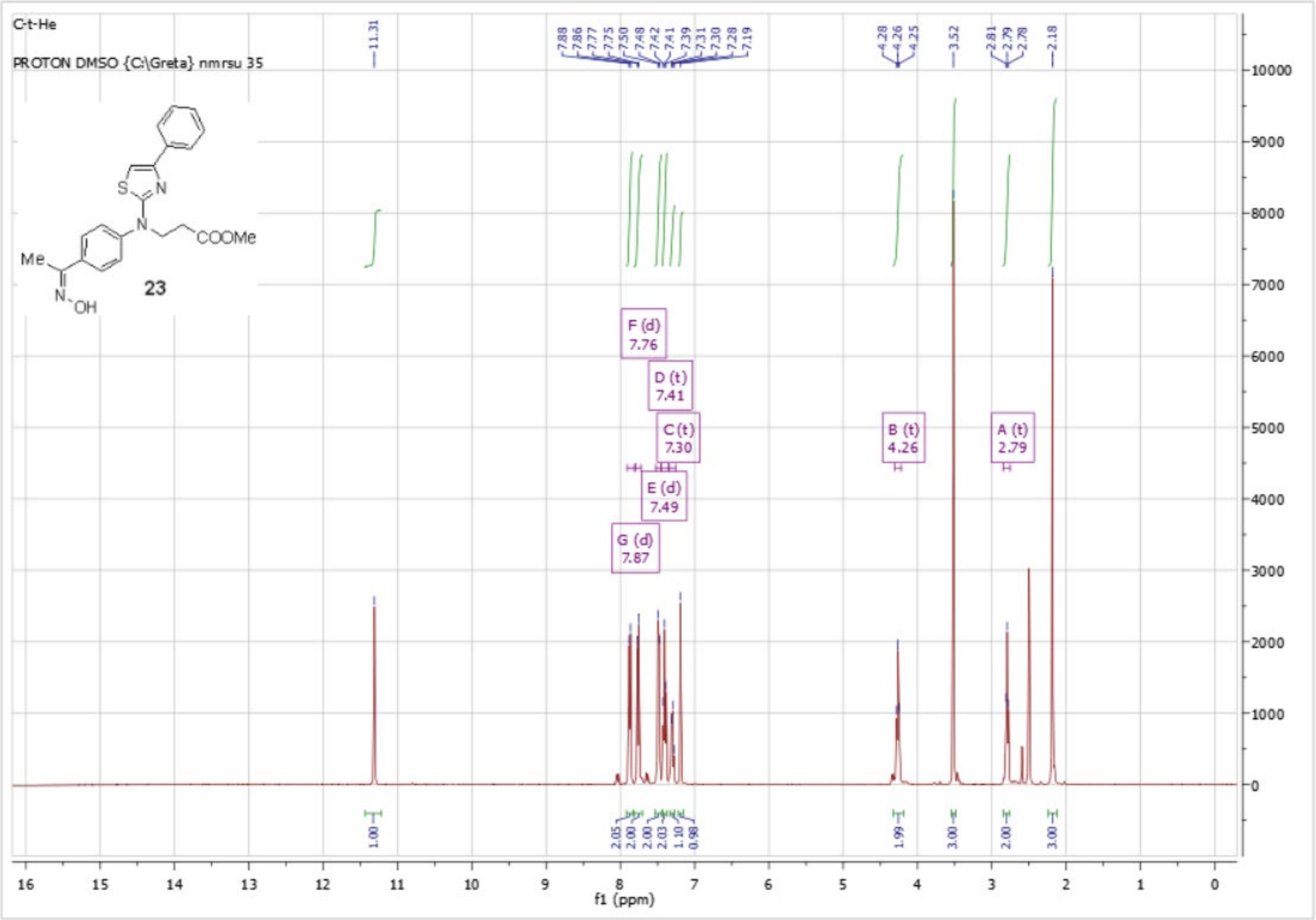

Figure S41. ^{1}H NMRspectrum of compound **23**.

*Methyl 3-/{4-[1-(hydroxyimino)ethyl]phenyl}(4-phenylthiazol-2-yl)amino/propanoate (**23**)*

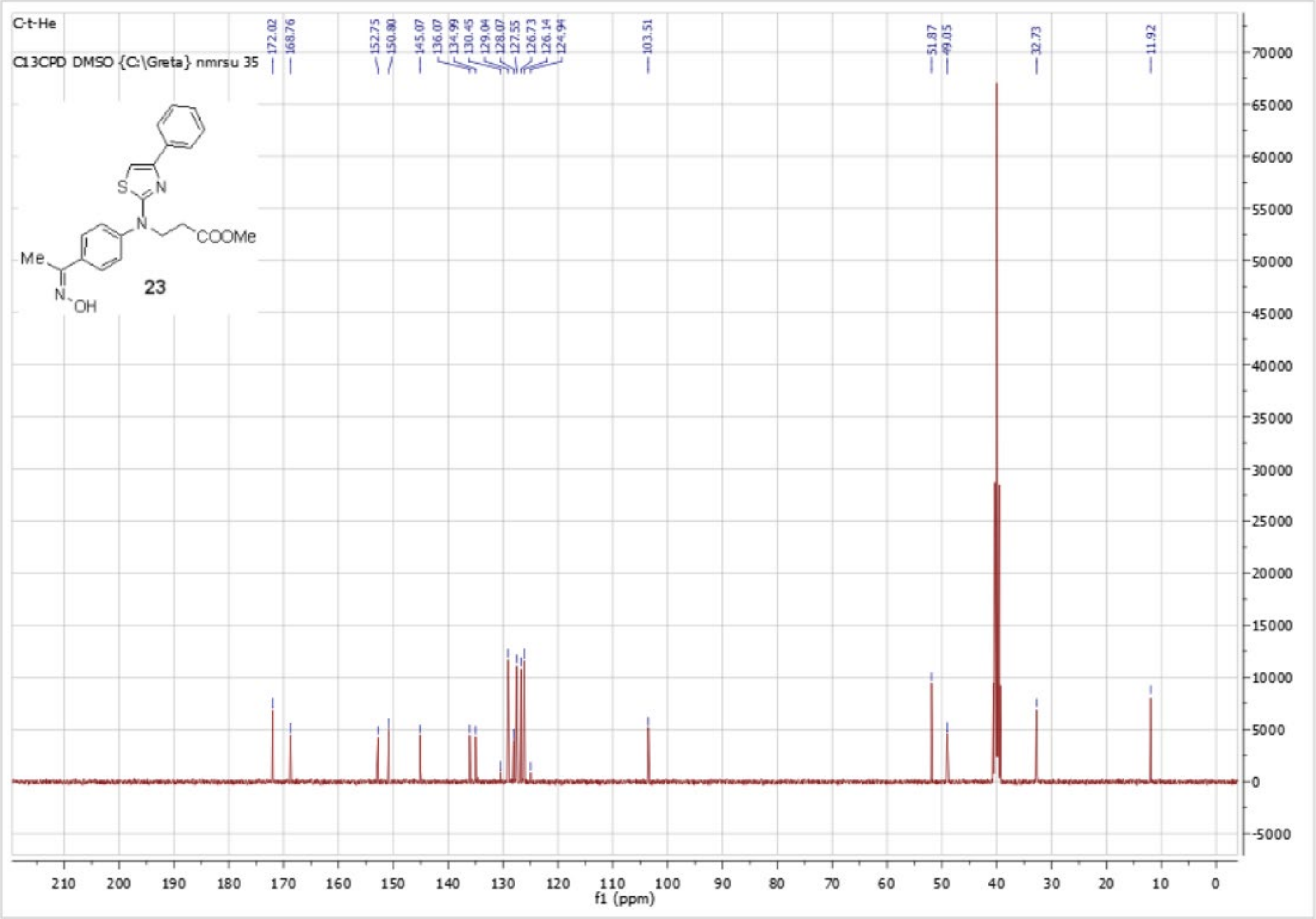

Figure S42. ^{13}C NMRspectrum of compound **23**.

*Methyl 3-/[4-(4-chlorophenyl)thiazol-2-yl]{4-[1-(hydroxyimino)ethyl]phenyl}amino/propanoate (**24**)*

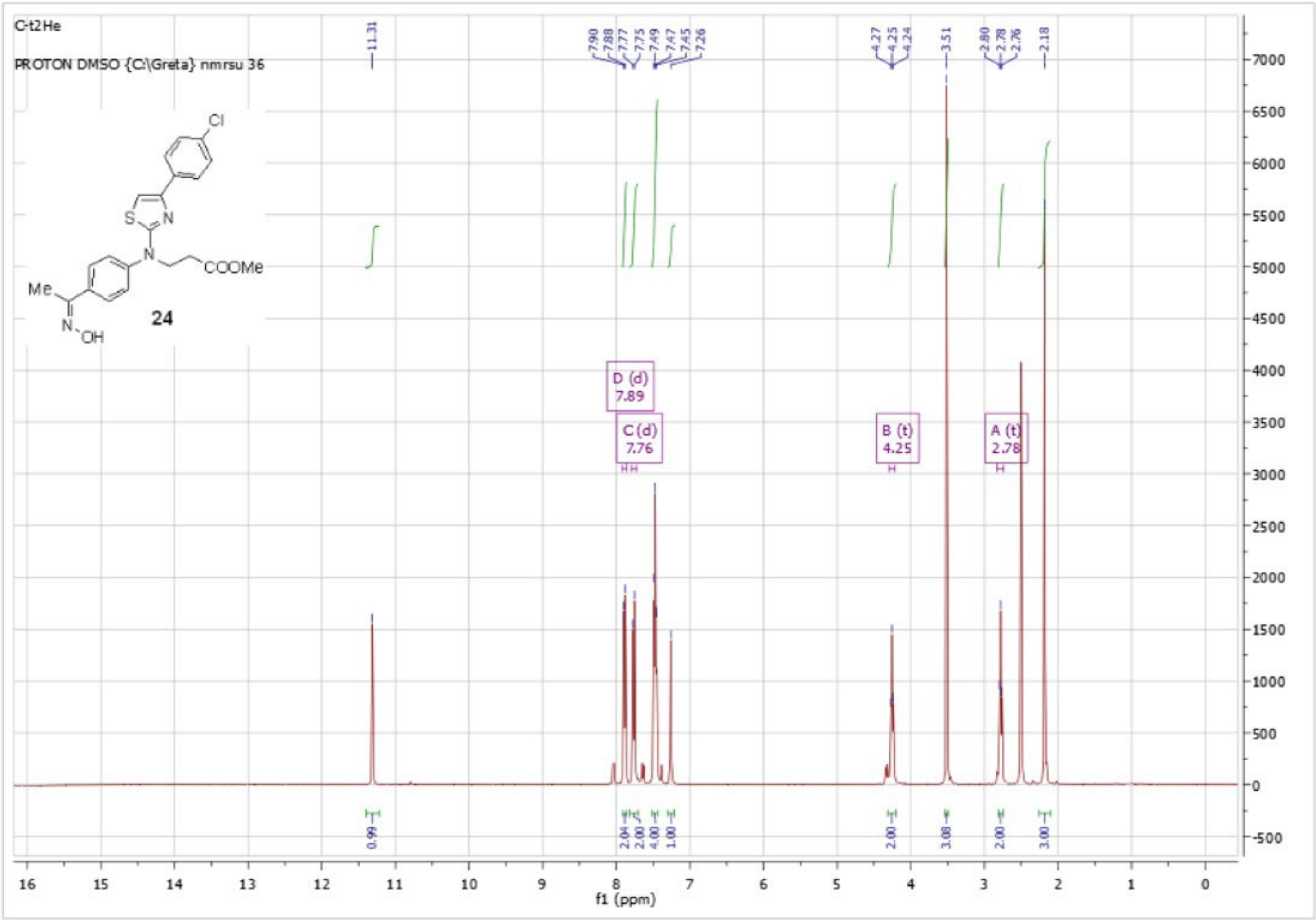

Figure S43. ^{1}H NMRspectrum of compound **24**.

*Methyl 3-/[4-(4-chlorophenyl)thiazol-2-yl]{4-[1-(hydroxyimino)ethyl]phenyl}amino/propanoate (**24**)*

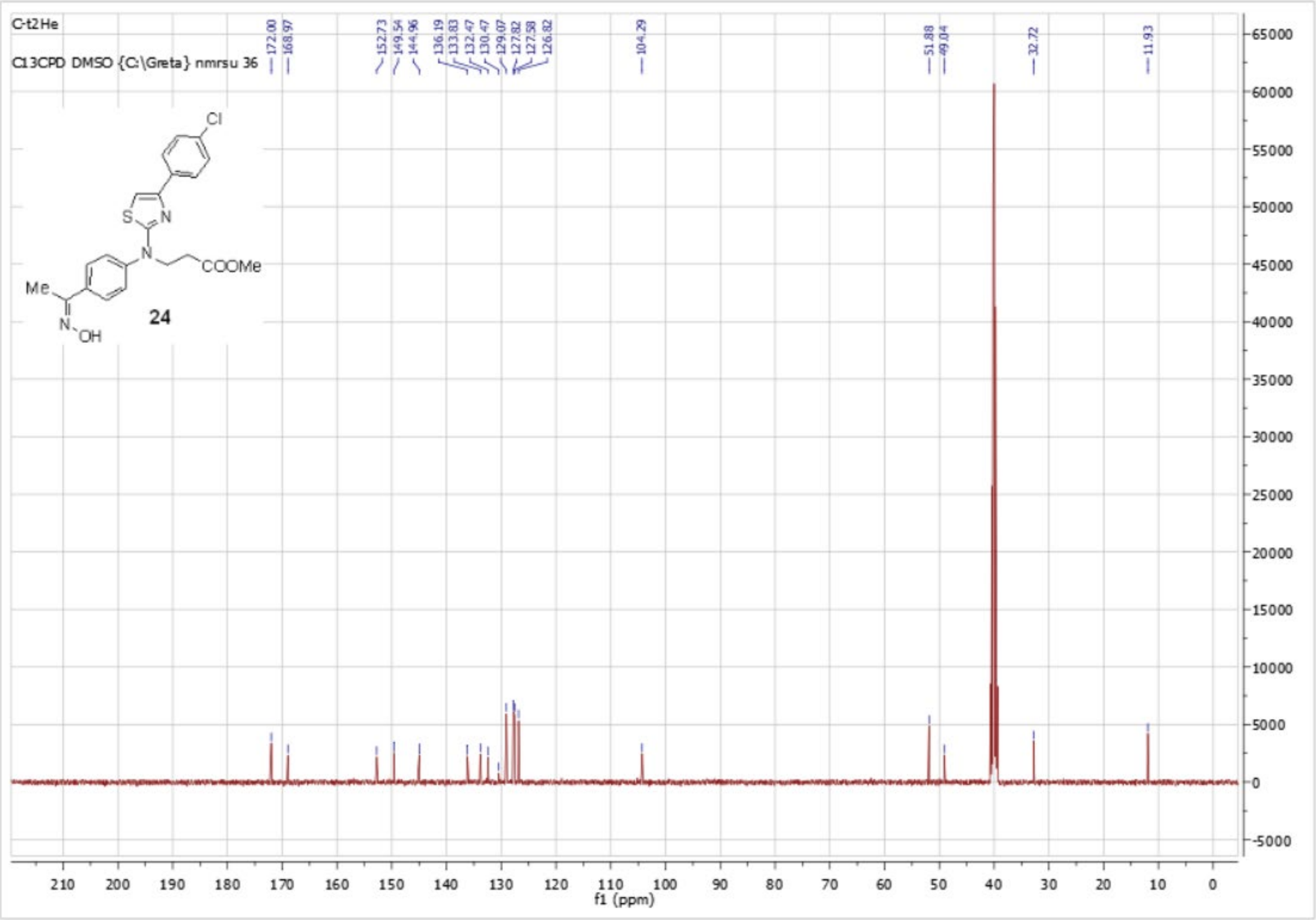

Figure S44. ^{13}C NMR spectrumof compound **24**.

*3-/{4-[1-(Hydroxyimino)ethyl]phenyl}(4-phenylthiazol-2-yl)amino/propanehydrazide (**25**)*

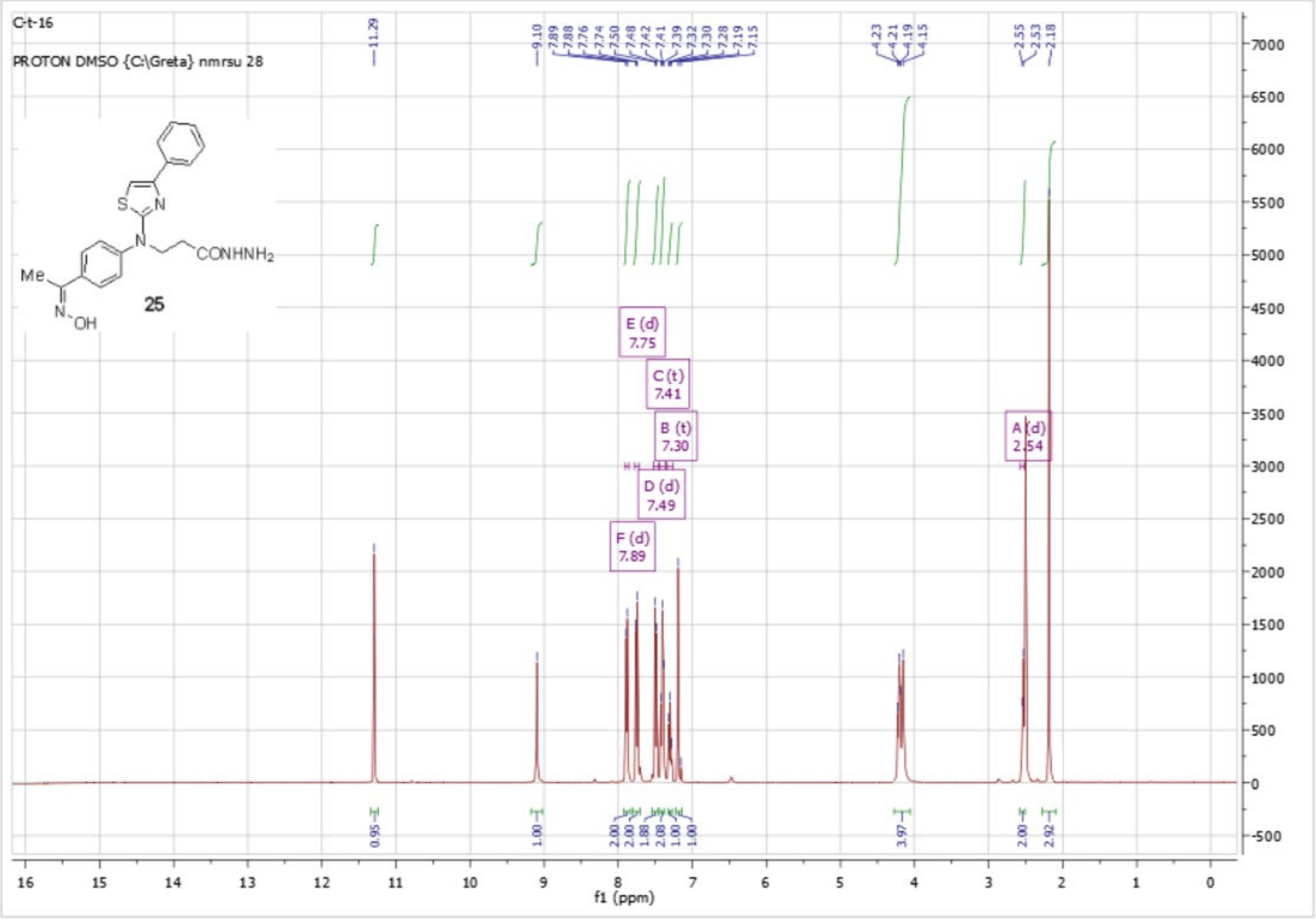

Figure S45. ¹H NMRspectrum of compound **25**.

*3-/{4-[1-(Hydroxyimino)ethyl]phenyl}(4-phenylthiazol-2-yl)amino/propanehydrazide (**25**)*

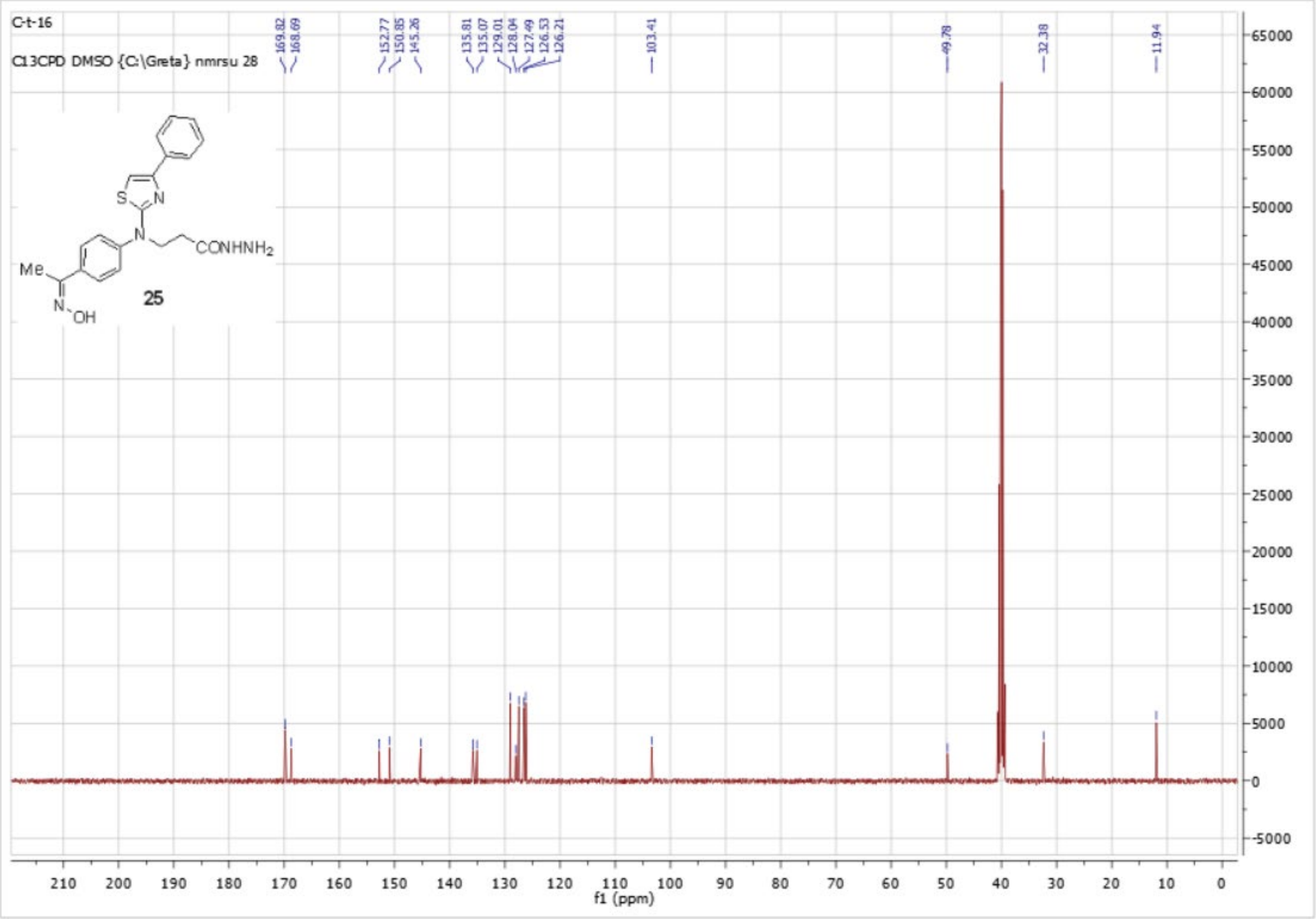

Figure S46. ^{13}C NMRspectrum of compound **25**.

*3-/[4-(4-Chlorophenyl)thiazol-2-yl]{4-[1-(hydroxyimino)ethyl]phenyl}amino/propanehydrazide (**26**)*

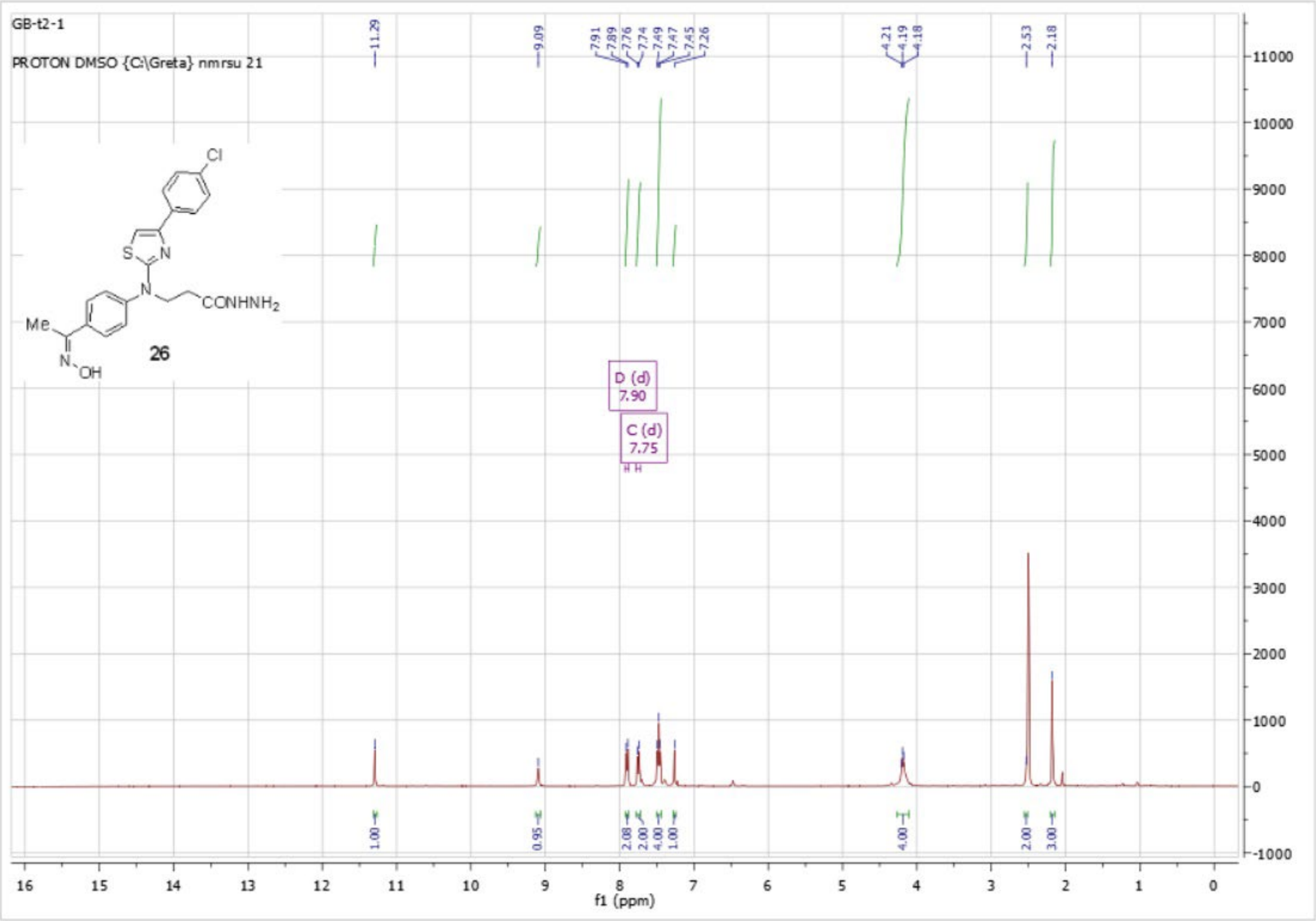

Figure S47. ^{1}H NMR spectrumof compound **26**.

*3-/[4-(4-Chlorophenyl)thiazol-2-yl]{4-[1-(hydroxyimino)ethyl]phenyl}amino/propanehydrazide (**26**)*

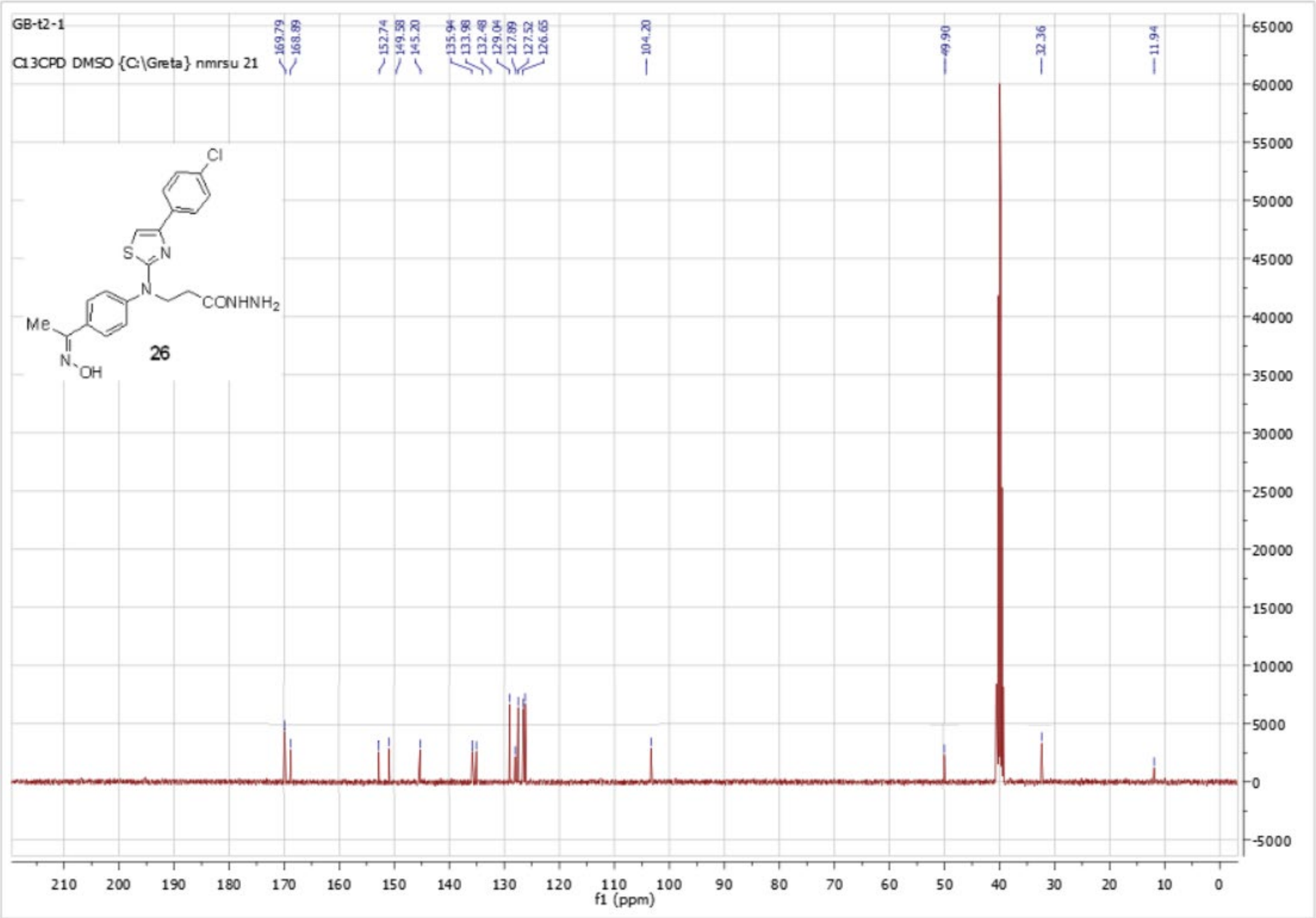

Figure S48. ^{13}C NMR spectrumof compound **26**.

*3-/{4-[1-(2-Phenylhydrazineylidene)ethyl]phenyl}(4-phenylthiazol-2-yl)amino/propanoic acid (**27**)*

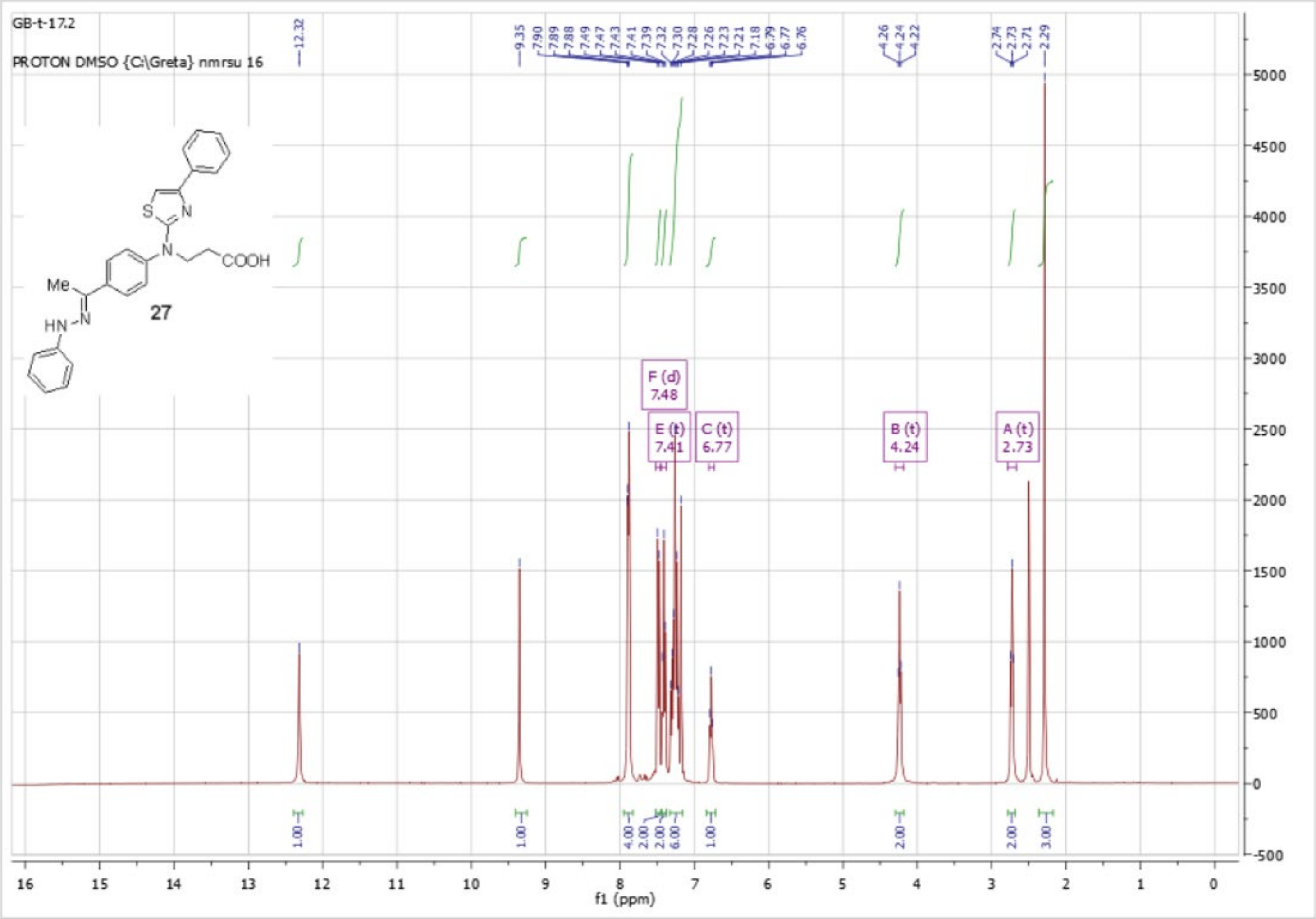

Figure S49. ^{1}H NMRspectrum of compound **27**.

*3-/{4-[1-(2-Phenylhydrazineylidene)ethyl]phenyl}(4-phenylthiazol-2-yl)amino/propanoic acid (**27**)*

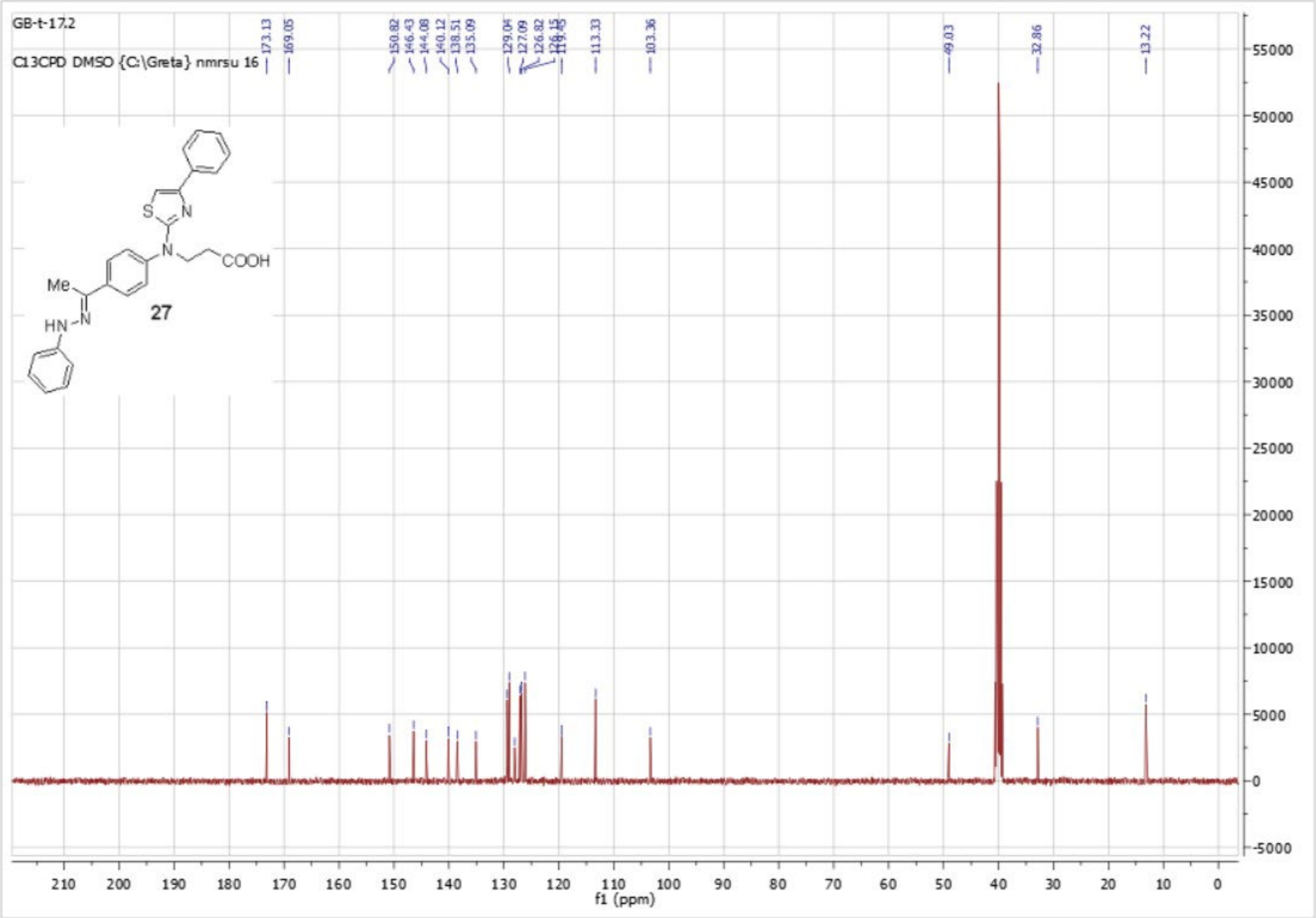

Figure S50. ^{13}C NMRspectrum of compound **27.**

*3-/[4-(4-Chlorophenyl)thiazol-2-yl]{4-[1-(2-phenylhydrazineylidene)ethyl]phenyl}amino/propanoic acid (**28**)*

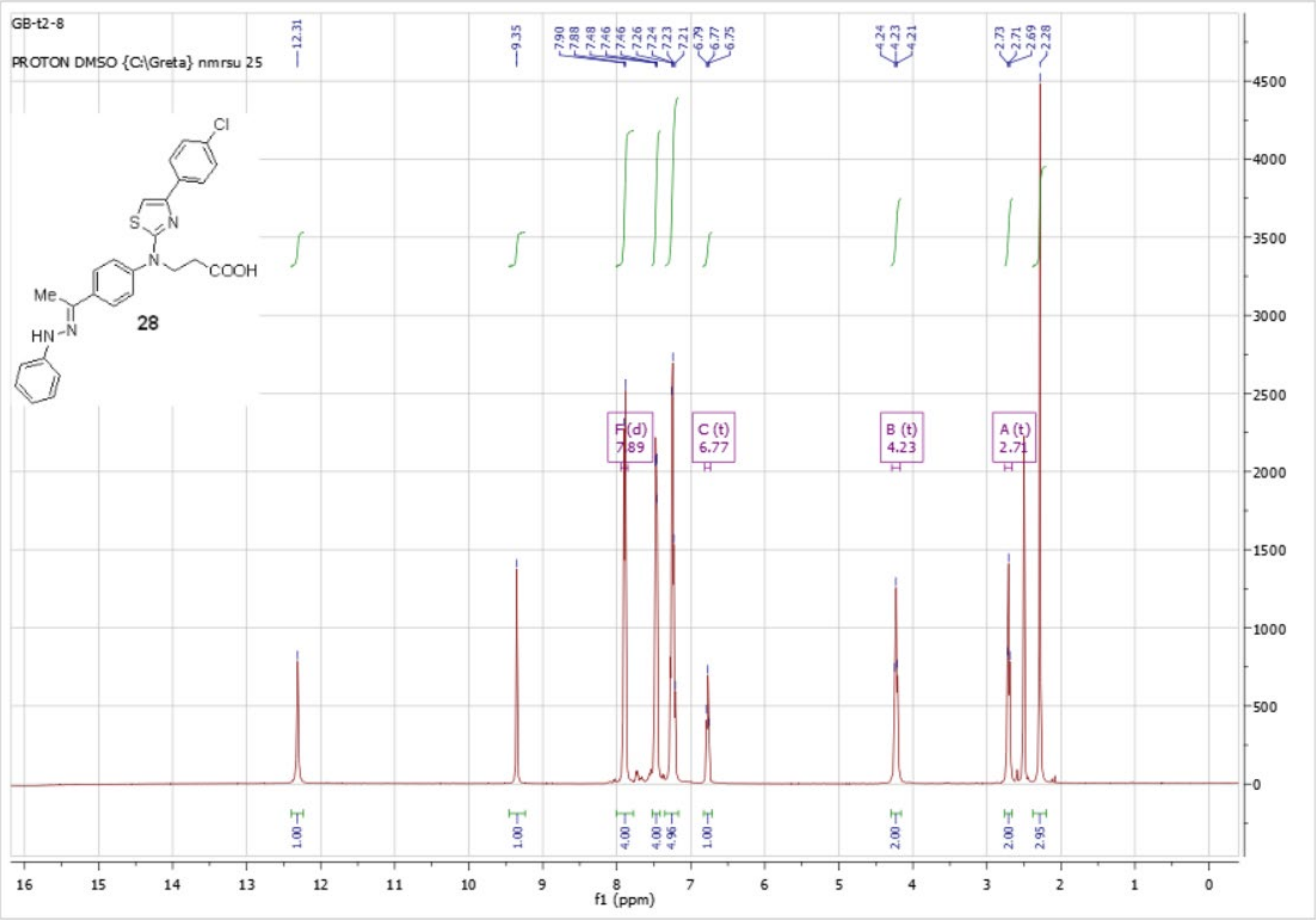

Figure S51. ^{1}H NMR spectrumof compound **28.**

*3-/[4-(4-Chlorophenyl)thiazol-2-yl]{4-[1-(2-phenylhydrazineylidene)ethyl]phenyl}amino/propanoic acid (**28**)*

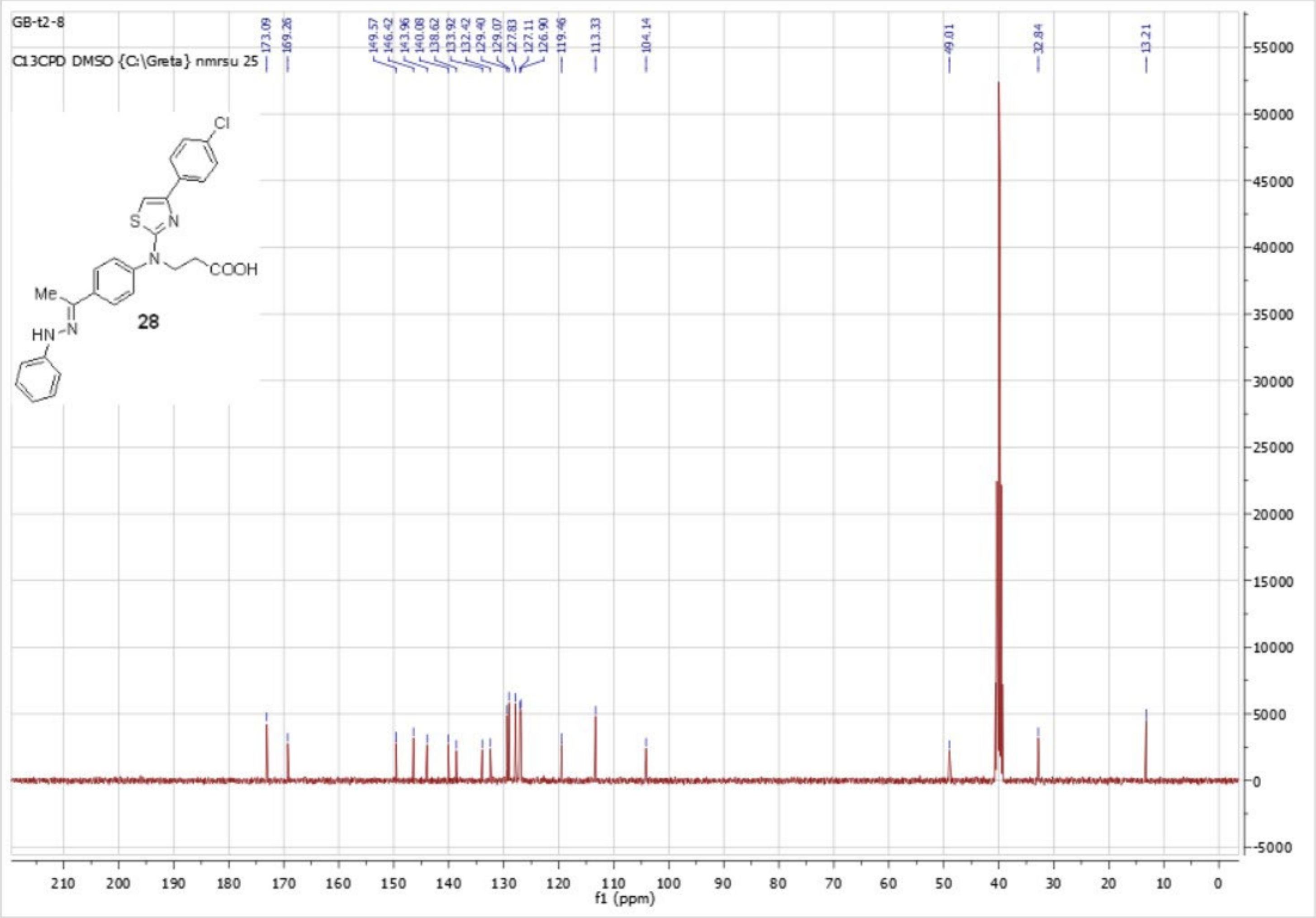

Figure S52. ^{13}C NMR spectrumof compound **28**.

*Methyl 3-[(4-acetylphenyl)(4-phenylthiazol-2-yl)amino]propanoate (**29**)*

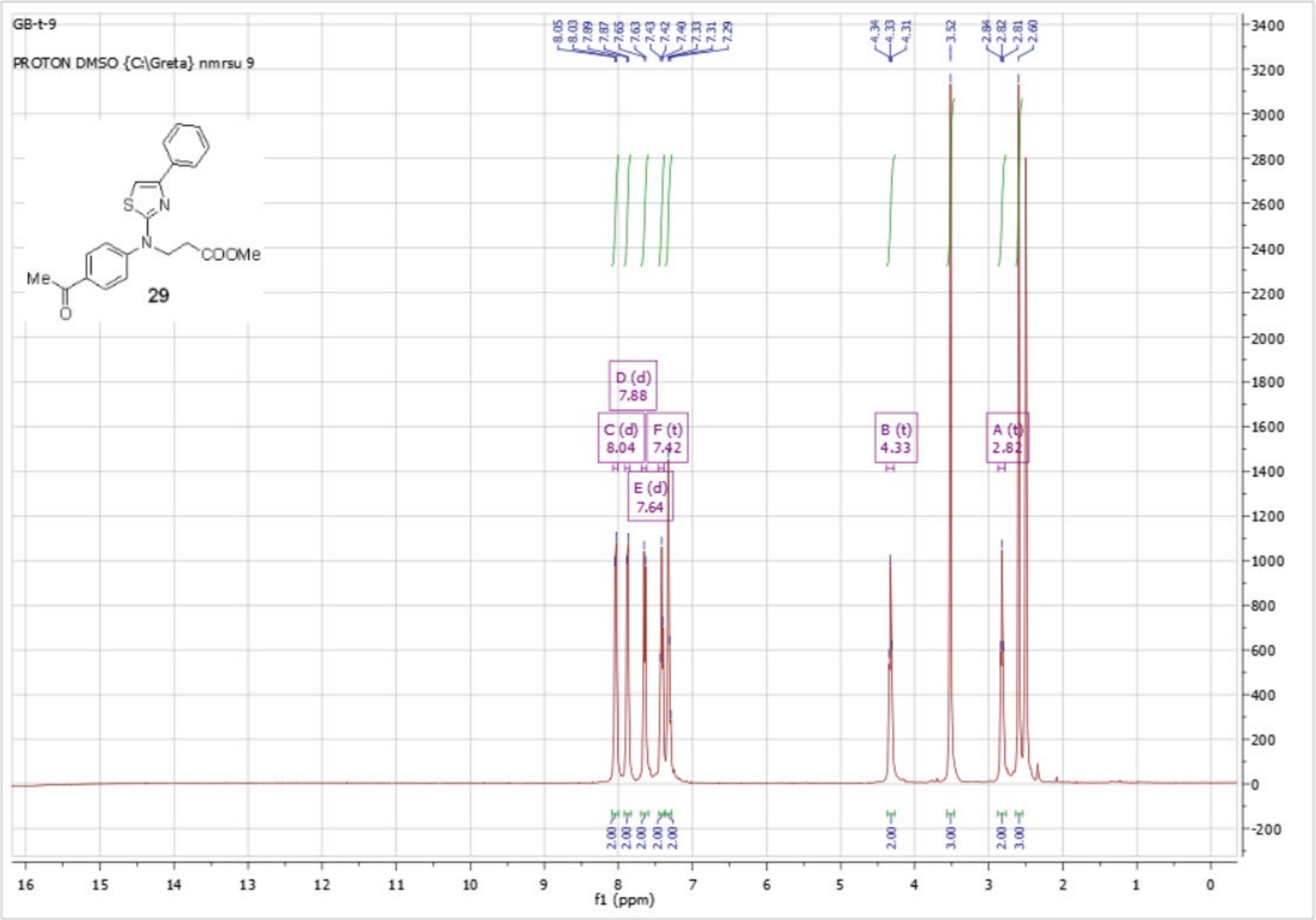

Figure S53. ¹H NMR spectrumof compound **29.**

*Methyl 3-[(4-acetylphenyl)(4-phenylthiazol-2-yl)amino]propanoate (**29**)*

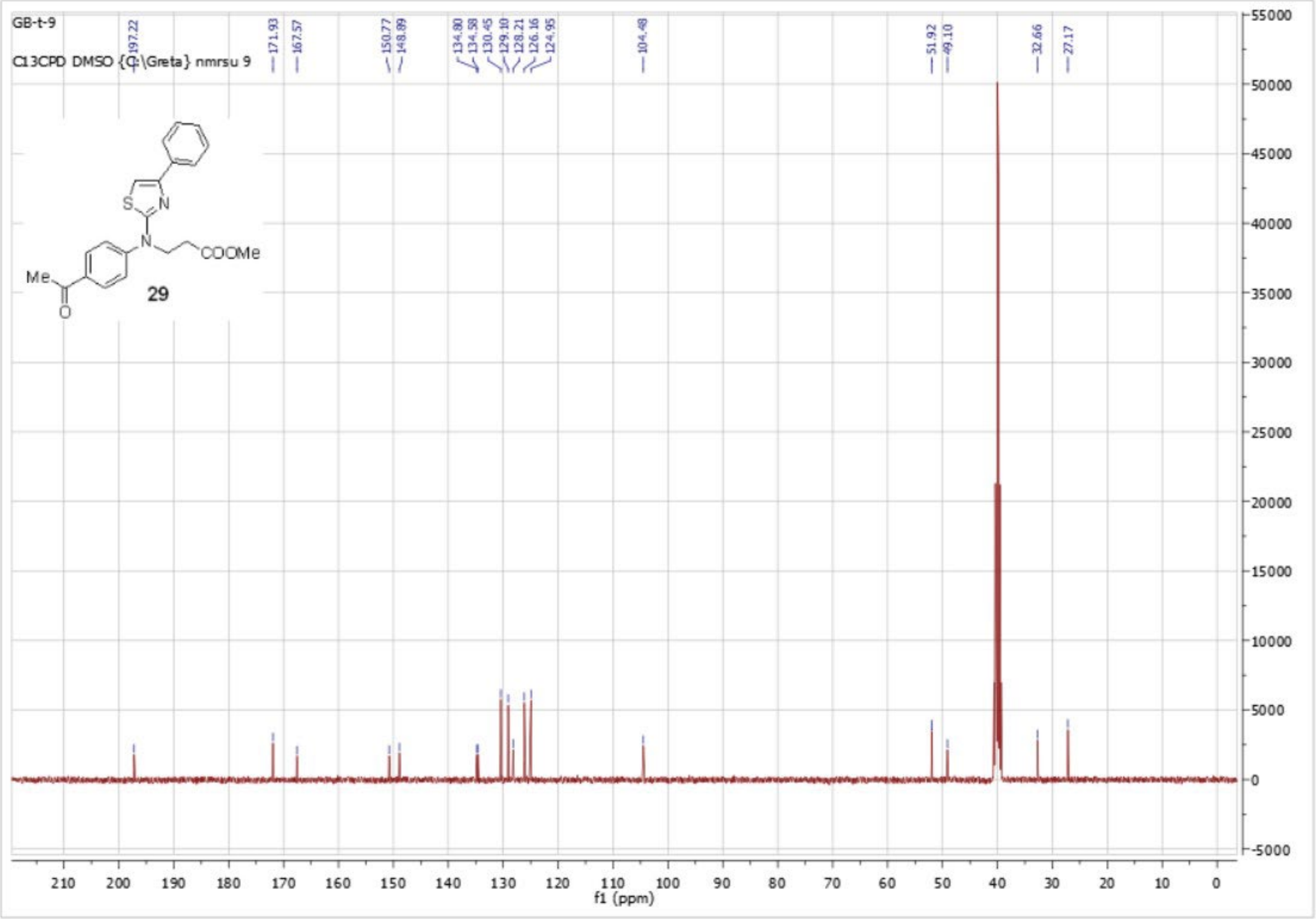

Figure S54. ^{13}C NMR spectrumof compound **29.**

*Methyl 3-{(4-acetylphenyl)[4-(4-chlorophenyl)thiazol-2-yl]amino}propanoate (**30**)*

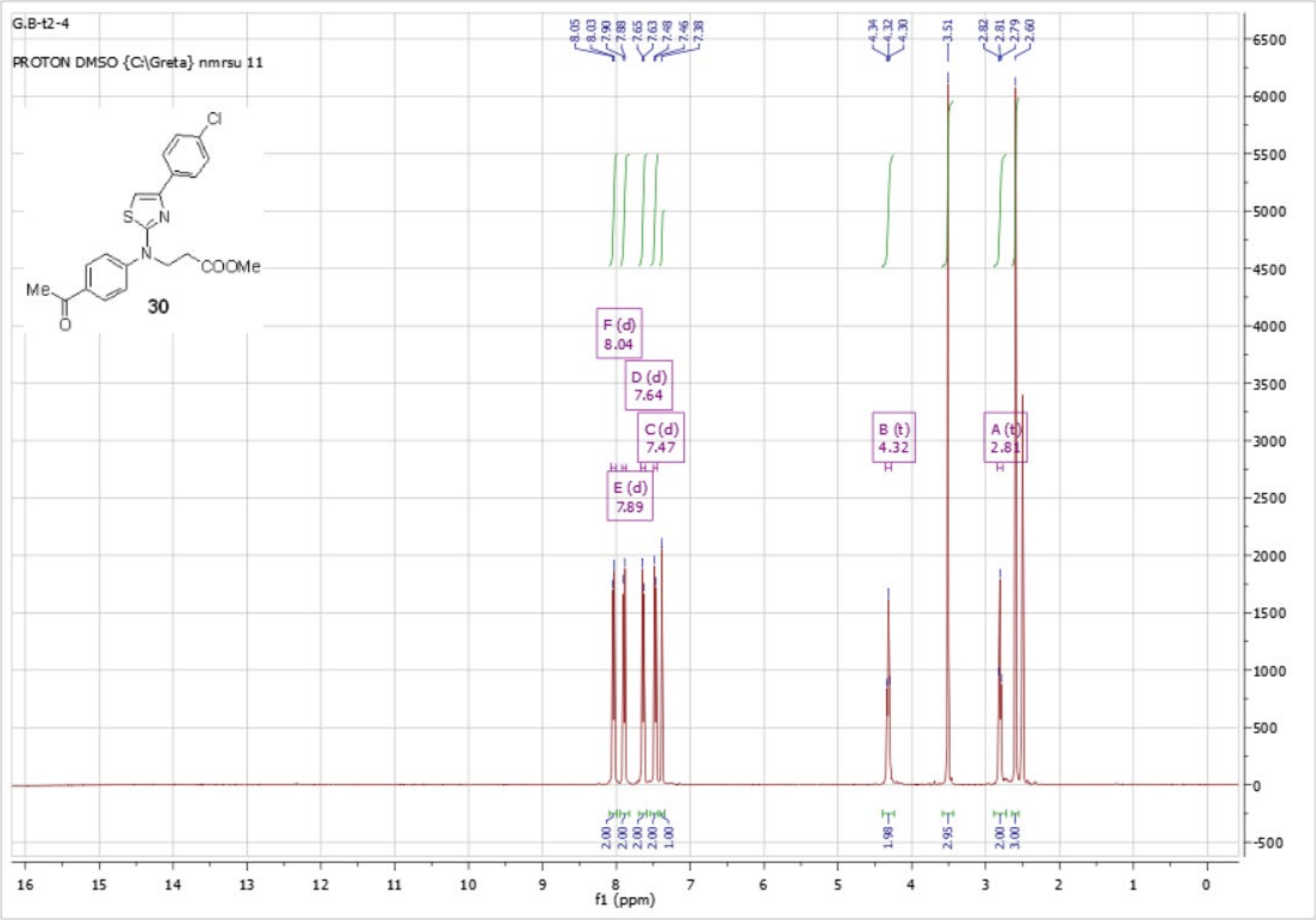

Figure S55. ^{1}H NMR spectrum of compound **30**.

*Methyl 3-{(4-acetylphenyl)[4-(4-chlorophenyl)thiazol-2-yl]amino}propanoate (**30**)*

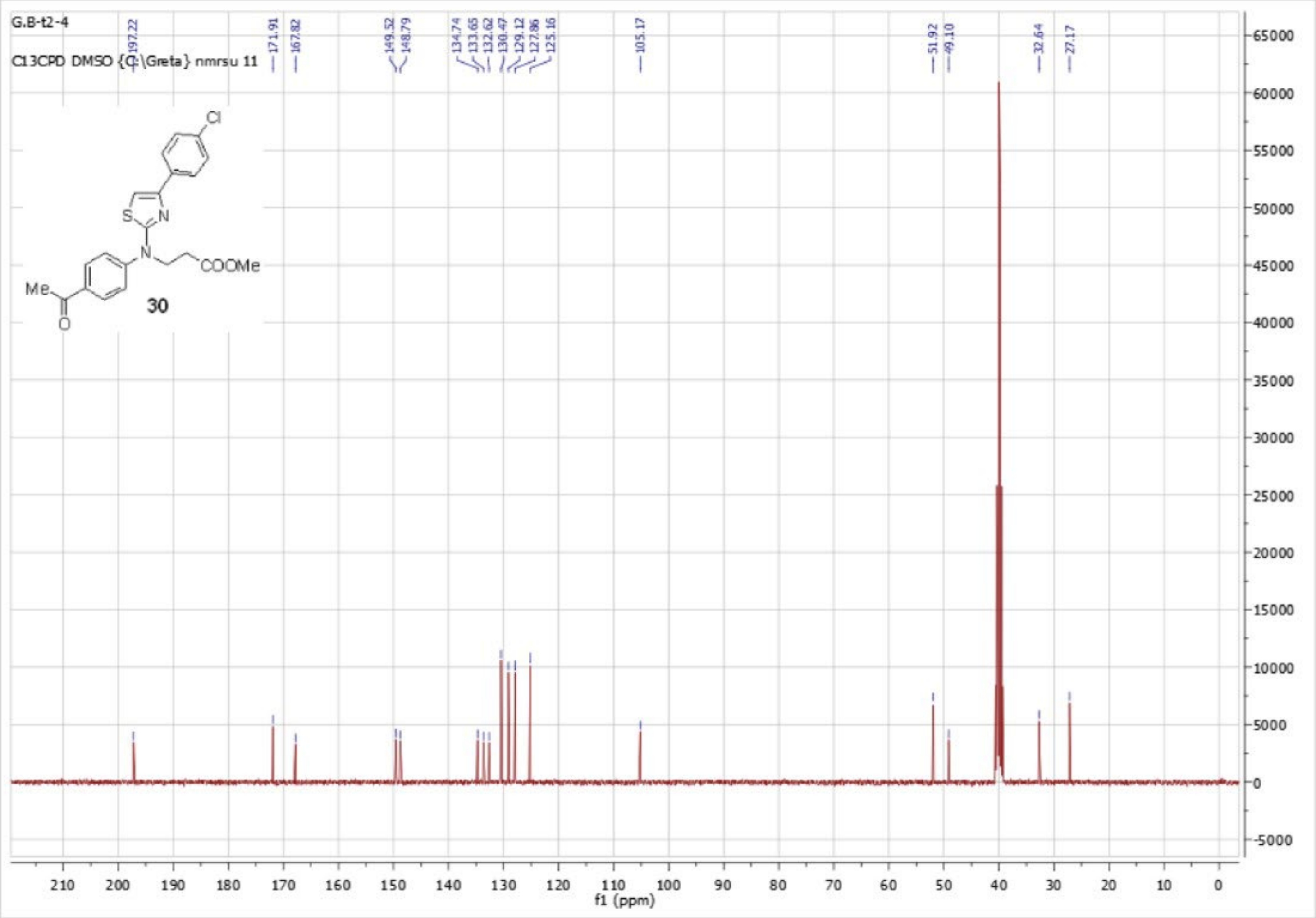

Figure S56. ^{13}C NMR spectrum of compound **30**.

*3-{[4-(1-Hydrazineylideneethyl)phenyl](4-phenylthiazol-2-yl)amino}propanehydrazide (**31**)*

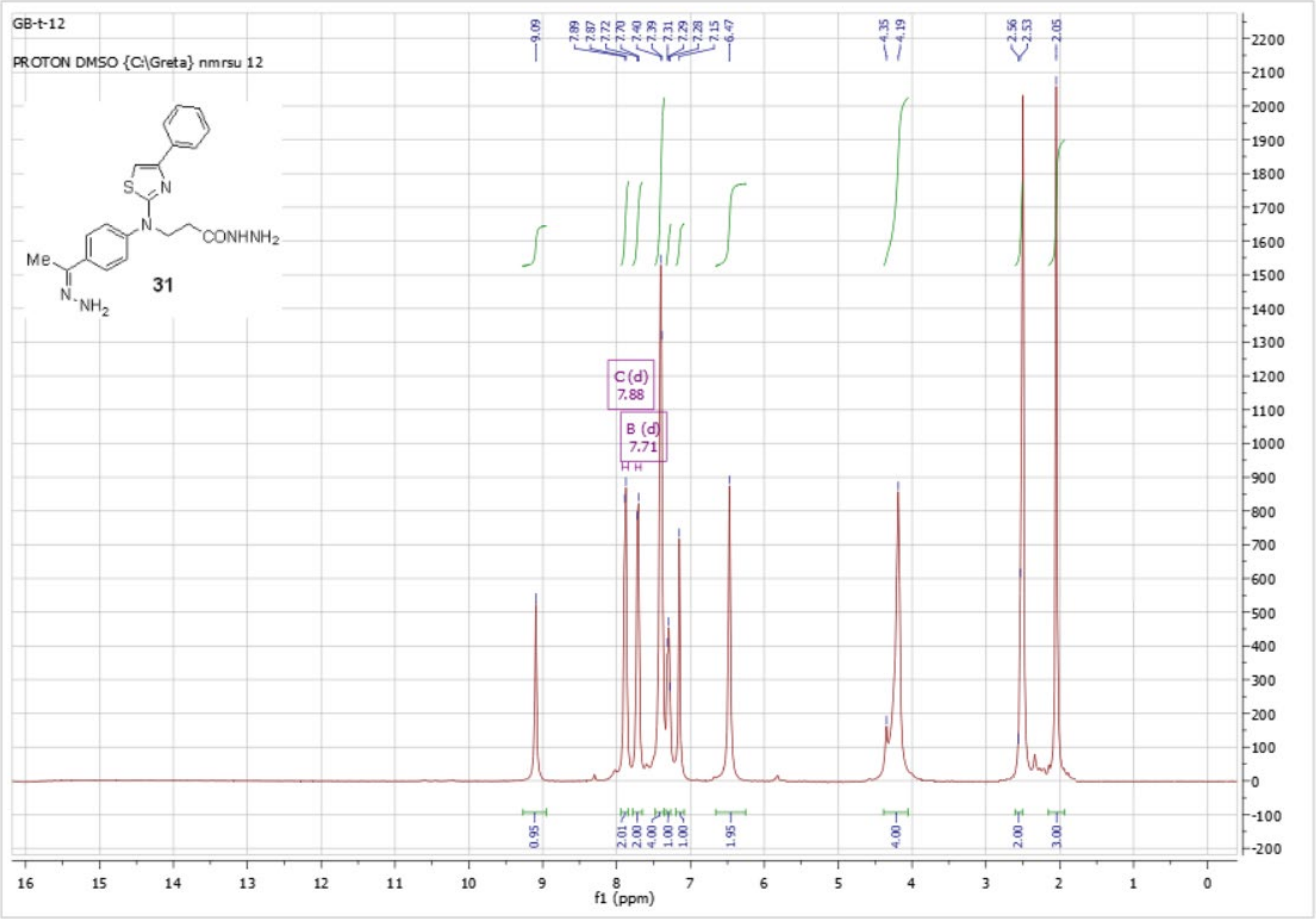

Figure S57. ^{1}H NMR spectrum of compound **31**.

*3-{[4-(1-Hydrazineylideneethyl)phenyl](4-phenylthiazol-2-yl)amino}propanehydrazide (**31**)*

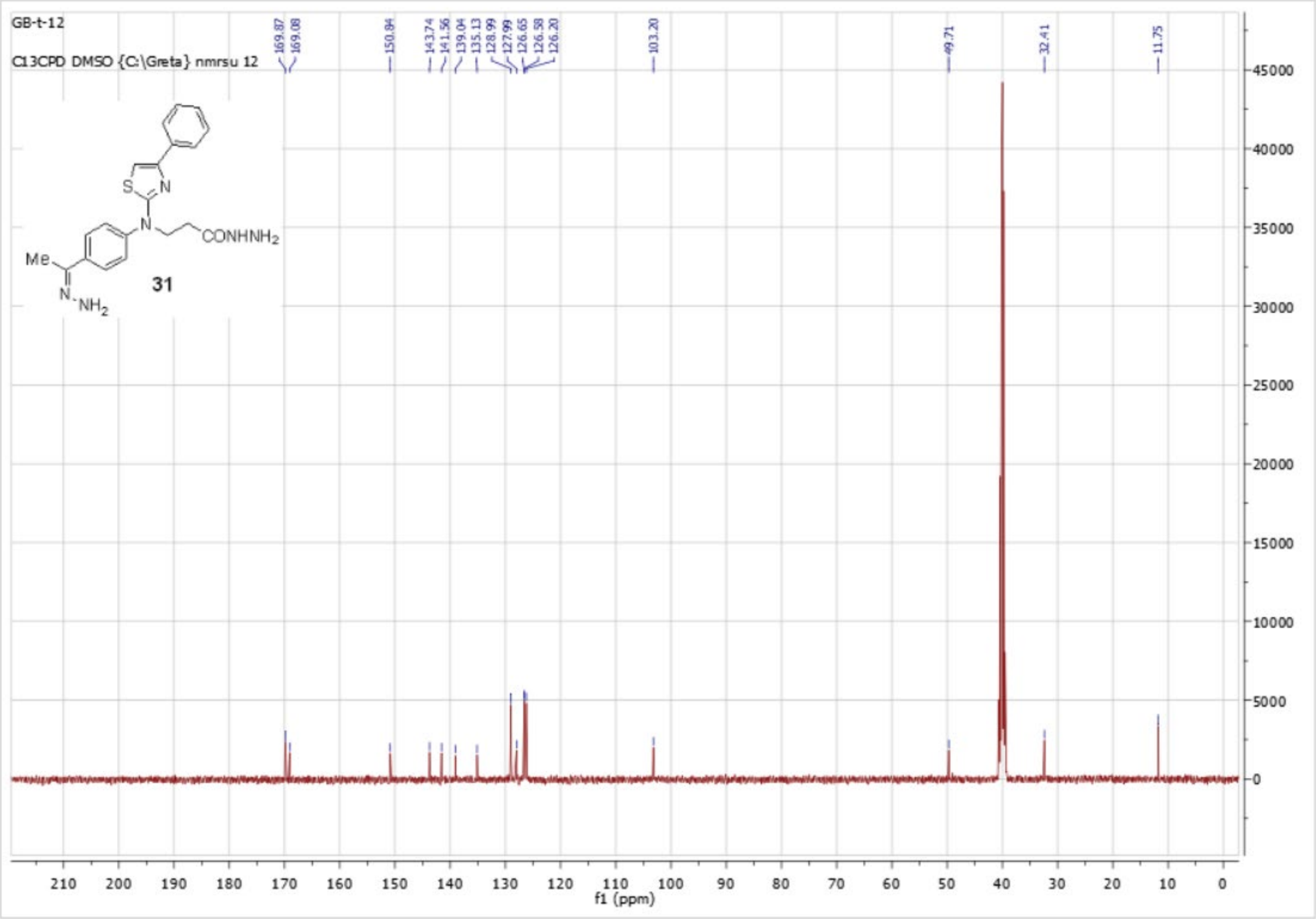

Figure S58. ^{13}C NMR spectrum of compound **31**.

*3-{[4-(4-Chlorophenyl)thiazol-2-yl][4-(1-hydrazineylideneethyl)phenyl]amino}propanehydrazide (**32**)*

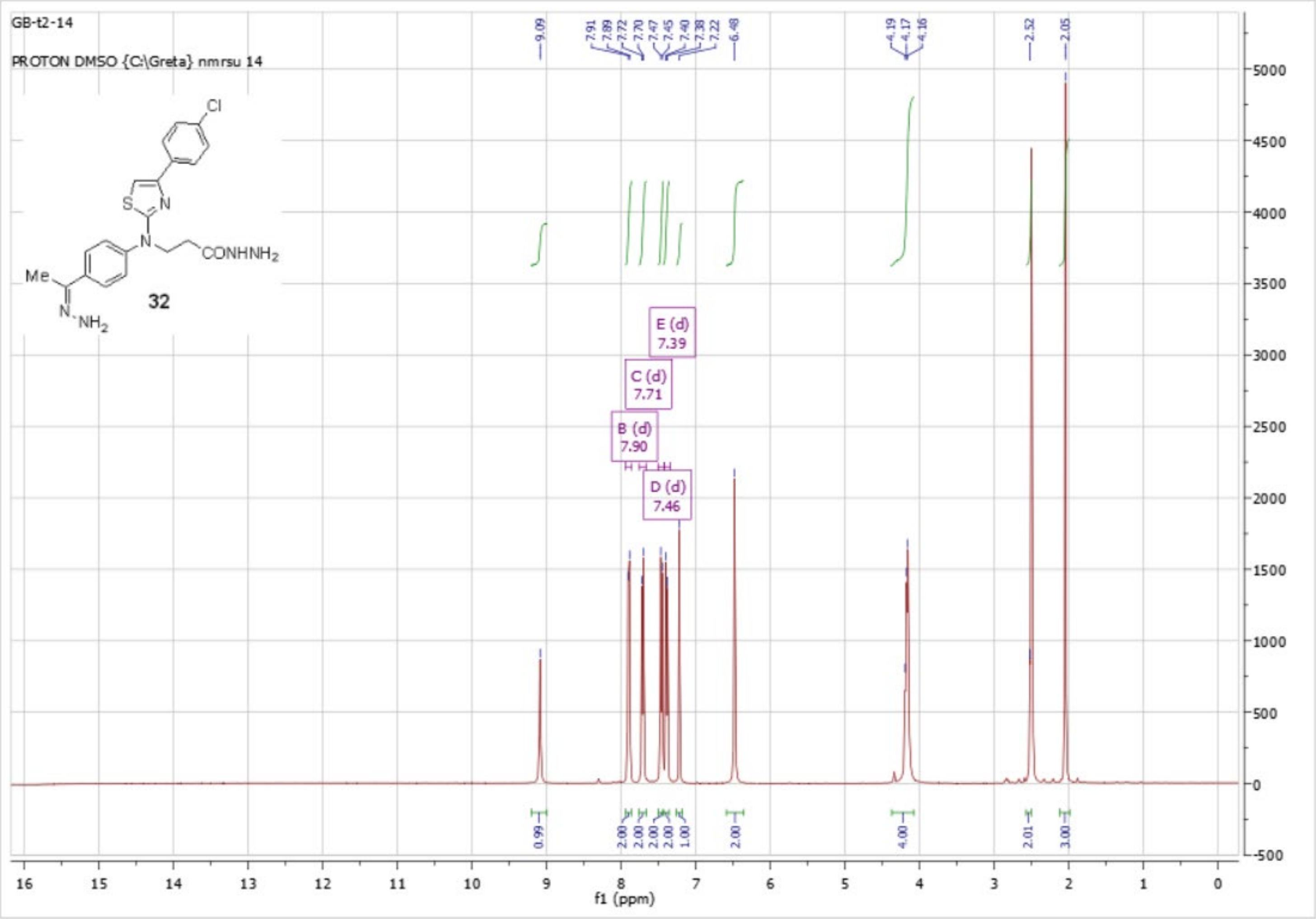

Figure S59. ^{1}H NMR spectrum of compound **32**.

*3-{[4-(4-Chlorophenyl)thiazol-2-yl][4-(1-hydrazineylideneethyl)phenyl]amino}propanehydrazide (**32**)*

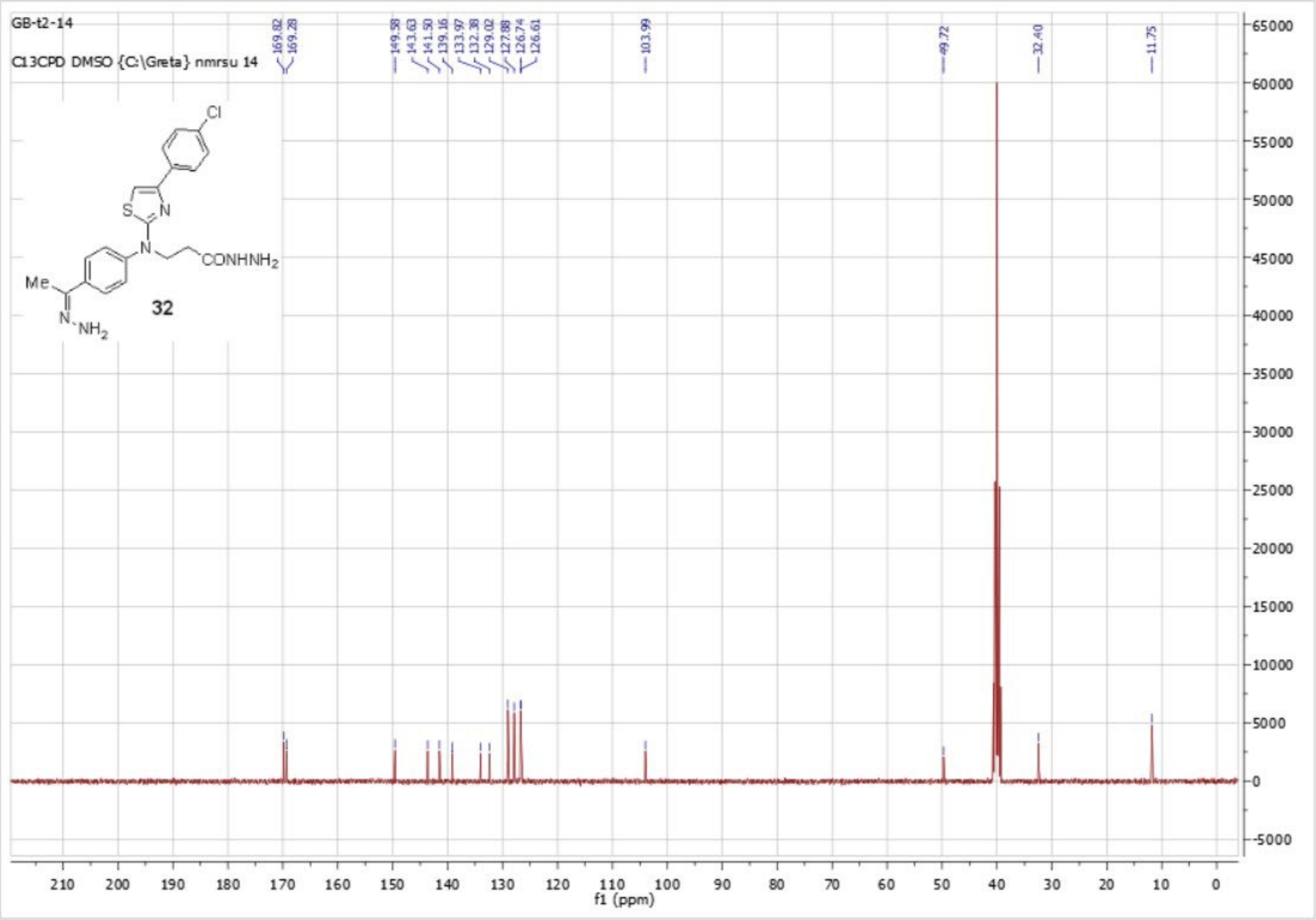

Figure S60. ^{13}C NMR spectrum of compound **32**.

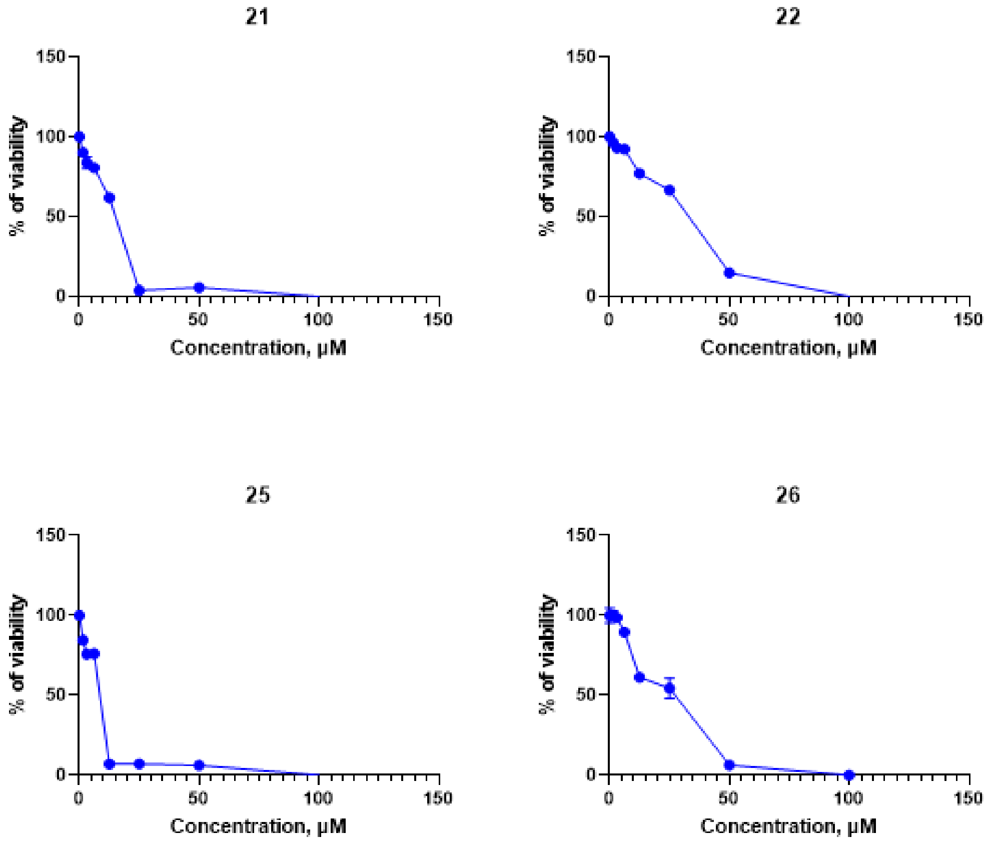

Figure S61. The dose-response kinetics of selected compounds **21**,**22** and **25**,**26**, the most promising candidates, in A549 human lung carcinoma cells. The A549 cells were exposed to increasing concentrations of the compounds for 24 hours, and viability was measured using the MTT assay. Data shown are mean ± SD values from three independent experiments for each group.

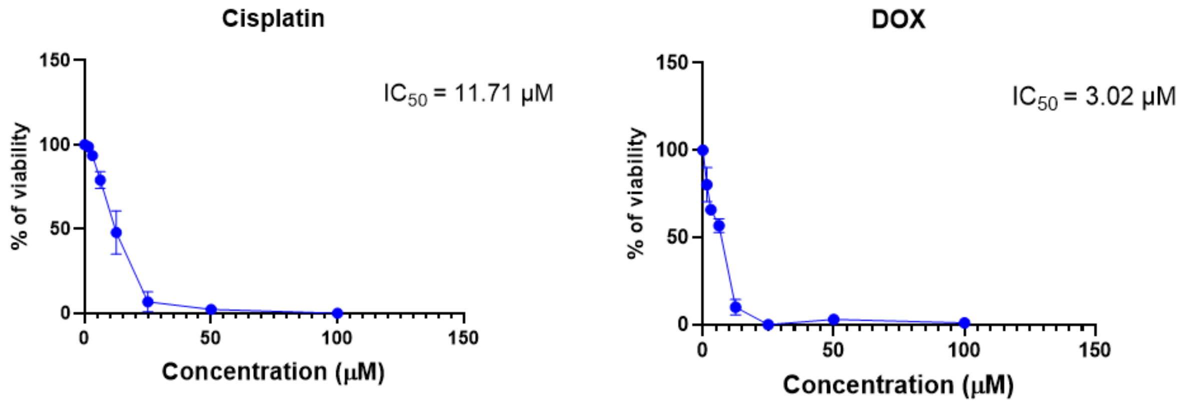

Figure S62. The dose-response kinetics and IC50 values of cisplatin and doxorubicin (DOX) in A549 cells. The cells were exposed with cisplatin (CP) and doxorubicin (DOX) for 24 hours and the viability was determined using MTT assay.

Table S1. Predicted binding free energy values (ΔG_{bin} kcal/mol) of synthesized cytotoxic hybrids with selected proteins overexpressed in cancer cells.

Target proteins					
**VEGRF-2**	**NR3A1**	**NR3A2**	**CK4**	**TopoII**	**HDAC2**
-7.6	-8.3	-8.4	-7.1	-7.1	-6.8
-7.9	-7.7	-8.5	-7.2	-7.5	-6.8
-7.8	-7.3	-8.1	-7.4	-7.6	-6.9
-8.1	-8.2	-7.7	-7.5	-7.3	-6.6
**-7.85**	**-7.88**	**-8.18**	**-7.30**	**-7.38**	**-6.78**

Proteins with their respective (PDB) entries: **15-LOX:** Arachidonate 15-lipoxygenase (3V99); **DHFR**: Dihydrofolate reductase (1DLS); **VEGRF-2**: Vascular endothelial growth factor receptor 2 (3VHE); **NR3A1**: Estrogen receptor beta (3ERT); **NR3A2**: Estrogen receptor beta (2QTU); **CK4**: Cyclin-dependent kinase 4 (1G3N); **TopoII:** Topoisomerase II (5GWK); **HDAC2:** histone deacetylase 2 (3MAX);. P avge.: Protein average. mean of the ΔG_{bin} values for the interactions of each protein with all the hybrids.
